# Fluostatins M–Q Featuring a 6-5-6-6 Ring Skeleton and High Oxidized A-Rings from Marine *Streptomyces* sp. PKU-MA00045

**DOI:** 10.3390/md16030087

**Published:** 2018-03-09

**Authors:** Jing Jin, Xiaoyan Yang, Tan Liu, Hua Xiao, Guiyang Wang, Mengjie Zhou, Fawang Liu, Yingtao Zhang, Dong Liu, Minghua Chen, Wei Cheng, Donghui Yang, Ming Ma

**Affiliations:** 1 State Key Laboratory of Natural and Biomimetic Drugs, Department of Natural Medicines, School of Pharmaceutical Sciences, Peking University, 38 Xueyuan Road, Haidian District, Beijing 100191, China; jinjing@bjmu.edu.cn (J.J.); yaner1888@163.com (X.Y.); lt900915@126.com (T.L.); pkuxh08@tom.com (H.X.); wangguiyang09@163.com (G.W.); zmj216@bjmu.edu.cn (M.Z.); fawang90@126.com (F.L.); zytao1988@163.com (Y.Z.); liudong_1982@126.com (D.L.); chengwei@bjmu.edu.cn (W.C.); 2 Institute of Medicinal Biotechnology, Chinese Academy of Medical Sciences and Peking Union Medical College, 1 Tiantanxili, Beijing 100050, China; mingsunlight@sina.com

**Keywords:** aromatic polyketides, fluostatins, gene cluster, genome mining, marine actinomycetes

## Abstract

Aromatic polyketides from marine actinomycetes have received increasing attention due to their unusual structures and potent bioactivities. Compared to their terrestrial counterparts, marine aromatic polyketides have been less discovered and their structural and biological diversities are far from being fully investigated. In this study, we employed a PCR-based genome mining method to discover aromatic polyketides in our marine bacteria collection. Five new atypical angucyclinones, fluostatins M–Q (**1**–**5**) featuring a unique 6-5-6-6 ring skeleton, were discovered from one “positive” *Streptomyces* sp. PKU-MA00045. The structures of fluostatins M–Q (**1**–**5**) were elucidated based on comprehensive spectroscopic analyses and the crystallographic structure of fluostatin P (**4**), which contains the most oxidized A-ring, was solved by X-ray diffraction analysis with Cu Kα radiation. Compared to the published 16 fluostatin analogues, fluostatins M–Q (**1**–**5**) contained a different methoxy group attached at C-7 and hydroxy group attached at C-4, enriching the structural diversity of aromatic polyketides from marine actinomycetes. Genome sequencing of *Streptomyces* sp. PKU-MA00045 revealed the biosynthetic gene cluster of fluostatins M–Q (**1**–**5**), which contained different genes and gene organizations compared to known fluostatin gene clusters, facilitating the investigation of the biosynthesis of the unique 6-5-6-6 ring skeleton in all fluostatins.

## 1. Introduction

Marine actinomycetes distributed in various habitats including sea deposits, sponges, corals, molluscs, seagrasses and mangroves are emerging as important producers of structurally complex and bioactive natural products [1,2,3,4]. Since the first marine actinomycete, *Rhodococcus marinonascens*, was taxonomically described [5], new genera and species of marine actinomycetes have been extensively isolated and identified [2,6,7]. The new genus *Salinispora*, which is the first marine actinomycete characterized by seawater or sea salts-obligate, has become a model organism for natural products discovery [4]. Varied families of natural products from marine actinomycetes with diverse bioactivities have been recently reviewed [2,3,4,8]. In the natural products produced by marine actinomycetes, aromatic polyketides have received increasing attention especially in the last two decades. These natural products have been less discovered compared to their terrestrial counterparts; however, most of them possess unusual structures or potent bioactivities. Lomaiviticins A and B, which were discovered from *Salinispora pacifica*, contain unique dimeric diazobenzofluorene glycoside structures and show potent cytotoxicities against human cancer cell lines [4,9]; trioxacarcin D–F, which were discovered from marine *Streptomyces* sp. B8652, contain high oxidized polycyclic structures and show cytotoxicities, antibacterial and antimalarial activities [10]; and komodoquinone A, which was discovered from marine *Streptomyces* sp. KS3, is a new anthracycline polyketide and shows neuritogenic activity [11] (Figure S1). Representative new aromatic polyketides that were discovered from marine actinomycetes are summarized in Figure S1, highlighting marine actinomycetes as the important producers of this family of natural products.

**Figure 1:**
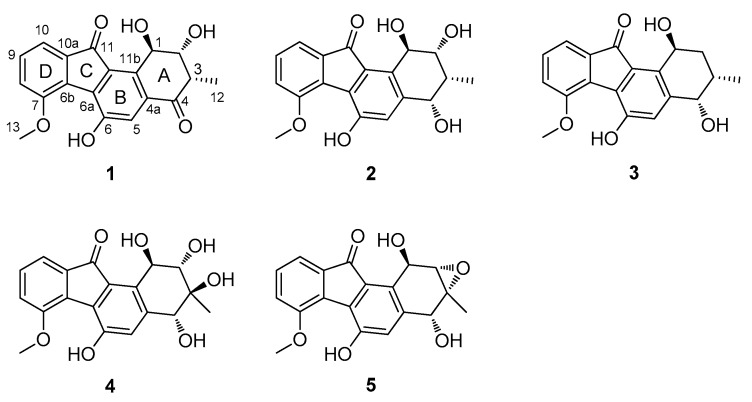
The structures of fluostatins M–Q (**1**–**5**) from *Streptomyces* sp. PKU-MA00045.

Aromatic polyketides from actinomycetes are mainly produced by type II polyketide synthases (PKS) [12,13]. The type II PKS contains three core enzymes: the ketosynthase (KS_α_), the chain length factor (KS_β_) and the acyl carrier protein (ACP). The KS_α_ and KS_β_ form a heterodimer catalyzing the ACP-templated decarboxylative condensations of acyl-CoA starter unit and malonyl-CoA extender units to produce the linear polyketide intermediate, then other enzymes in the biosynthetic pathway such as cyclases, aromatases, reductases, oxidases convert the linear polyketide intermediate into final aromatic polyketides [12,13,14]. The biosynthetic gene clusters of aromatic polyketides, containing the encoding genes of type II PKSs and other proteins, can be predicted based on bioinformatics analysis such as antiSMASH (antibiotics & Secondary Metabolite Analysis Shell) analysis [15] after genome sequencing, and the advanced methods based on searching and analyzing distributed domains within proteins have made the functional prediction more feasible and accurate [16,17,18]. In this study, we report the discovery of five new fluostatin analogues (**1**–**5**) from marine *Streptomyces* sp. PKU-MA00045, based on a PCR screening using the degenerate primers designed from the conserved sequences of KS_α_s and KS_β_s in the biosynthesis of four aromatic polyketides. Fluostatins M–Q (**1**–**5**) are atypical angucyclinones featuring a 6-5-6-6 ring skeleton and high oxidized A-rings (Figure 1), enriching the structural diversity of aromatic polyketides from marine actinomycetes. The biosynthetic gene cluster of fluostatins M–Q (**1**–**5**) was identified based on bioinformatics analysis after genome sequencing of *Streptomyces* sp. PKU-MA00045 and comparison with published gene clusters, setting the stage for future investigation of the biosynthesis of the unique 6-5-6-6 ring skeleton and high oxidized A-rings.

## 2. Results and Discussion

### 2.1. Genome Mining, Fermentation, Isolation and Phylogenetic Analysis

We have constructed a marine bacteria library containing strains isolated from sponges, corals and deposits collected from South China Sea [19]. To discover more aromatic polyketides from this library, we carried out a PCR screening targeting type II PKSs with genomic DNAs of strains in the library as the templates, using the degenerate primers designed from the conserved sequences of KS_α_s and KS_β_s in tetracenomycin, daunorubicin, actinorhodin and fredericamycin biosynthesis [20]. In total, 167 strains in the library were screened and 12 strains were identified as the “positive hits”, whose PCR products were analyzed by the agarose gel electrophoresis (Figure S2). All 12 PCR products were sequenced and confirmed to encode type II PKSs with high sequence identities to known homologues (Table S1).

It is well known that the metabolite profile of a given strain is medium dependent. To investigate the chemotypes of the 12 potential aromatic polyketides producers, we fermented the 12 strains in small-scale (50 mL) using four different media (see Section 3), and analyzed the crude extracts after the small-scale fermentation by HPLC. The HPLC analysis showed that the crude extract of strain PKU-MA00045 in medium M4 provided abundant natural products under the UV detection at 254 nm. Thus, a large-scale fermentation (15 L) of strain PKU-MA00045 in medium M4 was carried out. Five new fluostatin analogues (**1**–**5**) were isolated with a combination of chromatographic methods, and their structures were elucidated with comprehensive spectroscopic analyses. Strain PKU-MA00045 was identified as one *Streptomyces* species based on the phylogenetic analysis by the sequence alignments of 16S rRNAs with different *Streptomyces* homologues (Figure S3), adding more evidence to the conclusion that angucyclines and angucyclinones are exclusively discovered from actinomycetes [21].

### 2.2. Structural Elucidation of Compounds ***1***–***5*** and Biological Activity Assays

**Figure 2:**
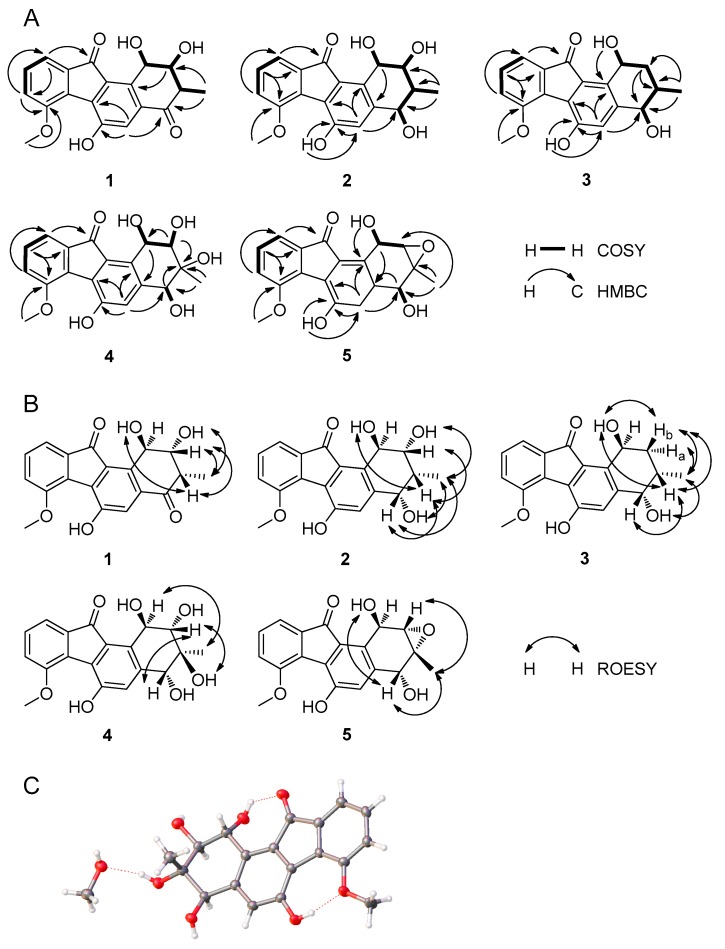
The key COSY, HMBC and ROESY correlations of fluostatins M–Q (**1**–**5**) and the crystallographic structure of fluostatin P (**4**). (**A**) The key COSY and HMBC correlations of fluostatins M–Q (**1**–**5**). (**B**) The key ROESY correlations of fluostatins M–Q (**1**–**5**). (**C**) The crystallographic structure of fluostatin P (**4**).

**Figure 3:**
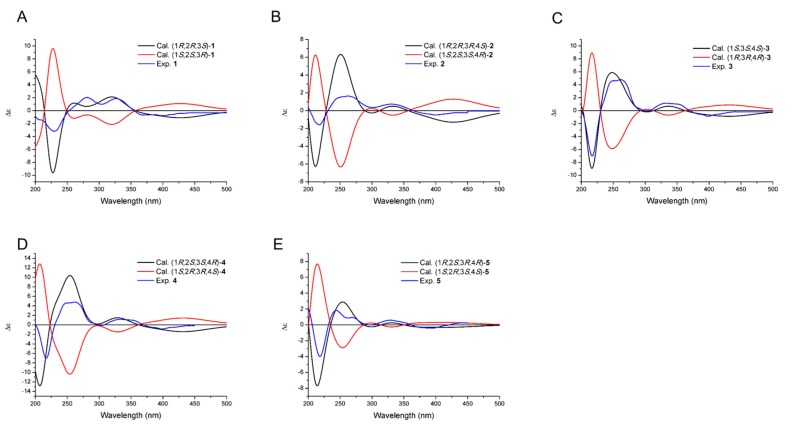
The calculated and experimental electronic circular dichroism (ECD) spectra of fluostatins M–Q (**1**–**5**). (**A**) The ECD spectra of fluostatin M (**1**). (**B**) The ECD spectra of fluostatin N (**2**). (**C**) The ECD spectra of fluostatin O (**3**). (**D**) The ECD spectra of fluostatin P (**4**). (**E**) The ECD spectra of fluostatin Q (**5**).

Compound **1** was obtained as orange solid. HRESIMS analysis afforded an [M − H]**^−^** ion at *m*/*z* 339.0870, giving the molecular formula of **1** as C_19_H_16_O_6_. The ^1^H-NMR and ^13^C-NMR spectra of **1** resembled that of fluostatin B [22] except that **1** gave additional resonances at *δ*_H_ 4.11 (s, H_3_-13) and *δ*_C_ 57.5 (C-13) attributing to one methyl group (Table 1 and Table 2), suggesting that **1** only contains one more methyl group than fluostatin B. The correlation between the resonances at *δ*_H_ 4.11 (s, H_3_-13) and *δ*_C_ 57.5 (C-13) in the HSQC spectrum in **1** confirmed the occurrence of the methyl group, and the correlation between H-13 and C-7 in the HMBC spectrum in **1** confirmed the methylation of the hydroxy group at C-7 (Figure 2A). Therefore, the planar structure of **1** was identified as 7-*O*-methyl-fluostatin B. In the ROESY spectrum of **1**, the correlations of H-12 with H-2, H-12 with 2-OH suggested that the methyl group attached at C-3, which is a larger moiety than H-3, took a more stable equatorial conformation; the correlation of H-3 with H-2 and the absence of correlation of H-3 with 2-OH suggested that the axial H-3 and equatorial H-2 were on the same side of A-ring; the correlation of axial H-3 with 1-OH instead of H-1 (the signal of 1-OH overlapped with that of H-1), which was confirmed by the absence of the correlation in the ROESY spectrum with D_2_O added (Figure S11), suggested that H-3 and 1-OH were on the same side of A-ring (Figure 2B). Therefore, the absolute configuration of **1** could be either (1*R*, 2*R*, 3*S*) or (1*S*, 2*S*, 3*R*). The experimental electronic circular dichroism (ECD) spectrum of **1** matched that of the calculated ECD spectrum of (1*R*, 2*R*, 3*S*)-**1** instead of that of (1*S*, 2*S*, 3*R*)-**1** (Figure 3A), identifying the absolute configuration of **1** as (1*R*, 2*R*, 3*S*). Thus, compound **1** was identified as (1*R*, 2*R*, 3*S*)-7-*O*-methyl-fluostatin B and named as fluostatin M.

Compound **2** was obtained as orange solid. HRESIMS analysis afforded an [M − H]**^−^** ion at *m*/*z* 341.1023, giving the molecular formula of **2** as C_19_H_18_O_6_. The ^1^H-NMR spectrum of **2** resembled that of **1** except that two additional resonances at *δ*_H_ 4.41 (dd, *J* = 7.8, 4.0 Hz, H-4) and *δ*_H_ 4.81 (d, *J* = 8.0 Hz, 4-OH) occurred in **2**, and the resonances at *δ*_H_ 3.17 (qd, *J* = 6.8, 2.3 Hz, H-3), *δ*_H_ 7.40 (s, H-5), *δ*_H_ 4.01 (dd, *J* = 3.5, 2.3 Hz, H-2) and *δ*_H_ 5.46 (d, *J* = 3.5 Hz, H-1) in **1** shifted upfield to *δ*_H_ 2.24 (m, H-3), *δ*_H_ 7.00 (s, H-5), *δ*_H_ 3.75 (td, *J* = 4.5, 2.3 Hz, H-2) and *δ*_H_ 5.12 (t, *J* = 4.3 Hz, H-1) in **2**, respectively (Table 1). The COSY spectrum of **2** showed that the resonances at *δ*_H_ 4.41 (dd, *J* = 7.8, 4.0 Hz, H-4), *δ*_H_ 4.81 (d, *J* = 8.0 Hz, 4-OH), *δ*_H_ 2.24 (m, H-3), *δ*_H_ 3.75 (td, *J* = 4.5, 2.3 Hz, H-2) and *δ*_H_ 5.12 (t, *J* = 4.3 Hz, H-1) formed one coupling system (Figure 2A). These different chemical shifts between the ^1^H-NMR spectra of **1** and **2** and the coupling system in the COSY spectrum of **2**, in combination with the molecular formula of **2**, suggested that the only difference between **1** and **2** was that the carbonyl group at C-4 in **1** was replaced by a hydroxy group in **2**. This was confirmed by that the resonance at *δ*_C_ 197.7 (C-4) in **1** was replaced by *δ*_C_ 69.5 (C-4) in **2**, the resonances at *δ*_C_ 41.6 (C-3) in **1** shifted upfield to *δ*_C_ 33.3 (C-3) in **2** and the resonances at *δ*_C_ 134.0 (C-4a) in **1** shifted downfield to *δ*_C_ 143.9 (C-4a) in **2**, in the ^13^C-NMR spectra of **1** and **2** (Table 2). The correlations of H-4 with C-5, H-4 with C-12, H-4 with C-2, 4-OH with C-4 and 4-OH with C-4a in the HMBC spectrum of **2** further confirmed the hydroxy group attached at C-4 (Figure 2A). Thus, the planar structure of **2** was identified. In the ROESY spectrum of **2**, the strong correlations of H-12 with H-4, H-12 with 4-OH suggested that the methyl group attached at C-3 took an equatorial conformation; the correlations of the axial H-3 with H-4, H-2 and 1-OH suggested that H-3, H-4, H-2 and 1-OH were on the same side of A-ring (Figure 2B). Therefore, the absolute configuration of **2** could be either (1*R*, 2*R*, 3*R*, 4*S*) or (1*S*, 2*S*, 3*S*, 4*R*). The experimental electronic circular dichroism (ECD) spectrum of **2** matched that of the calculated ECD spectrum of (1*R*, 2*R*, 3*R*, 4*S*)-**2** instead of that of (1*S*, 2*S*, 3*S*, 4*R*)-**2** (Figure 3B). Thus, compound **2**’s absolute configuration was identified as (1*R*, 2*R*, 3*R*, 4*S*) and named as fluostatin N.

Compound **3** was obtained as orange solid. HRESIMS analysis afforded an [M − H]**^−^** ion at *m*/*z* 325.1084, giving the molecular formula of **3** as C_19_H_18_O_5_. The ^1^H-NMR spectrum of **3** resembled that of **2** except that the resonance at *δ*_H_ 5.19 (d, *J* = 4.8 Hz, 2-OH) in **2** disappeared in **3**, the resonance at *δ*_H_ 3.75 (td, *J* = 4.5, 2.3 Hz, H-2) in **2** was replaced by *δ*_H_ 1.86 (td, *J* = 13.6, 4.0 Hz, H-2a) and *δ*_H_ 1.52 (dt, *J* = 13.6, 2.5 Hz, H-2b) in **3**, and the resonance at *δ*_H_ 5.12 (t, *J* = 4.3 Hz, H-1) in **2** shifted downfield to *δ*_H_ 5.30 (m, H-1) in **3** (Table 1). The ^13^C-NMR spectrum of **3** resembled that of **2** except that the resonance at *δ*_C_ 74.0 (C-2) in **2** was replaced by *δ*_C_ 33.0 (C-2) in **3**, the resonance at *δ*_C_ 66.2 (C-1) and *δ*_C_ 33.3 (C-3) in **2** shifted upfield to *δ*_C_ 60.4 (C-1) and *δ*_C_ 28.1 (C-3) in **3**, respectively (Table 2). These different chemical shifts and coupling patterns suggested that the only difference between **2** and **3** was that the hydroxy group attached at C-2 in **2** was replaced by one hydrogen in **3**. This was confirmed by the correlations from the COSY, HSQC and HMBC spectra of **3** (Figure 2A). In the ROESY spectrum of **3**, the correlations of H-12 with H-4, 4-OH, H-2a and H-2b suggested that the methyl group attached at C-3 took an equatorial conformation; the correlations of the axial H-3 with H-4, H-2b and 1-OH suggested that H-3, H-4, H-2 and 1-OH were on the same side of A-ring; the absence of the correlation of H-2a with 1-OH and the large coupling constants (*J* = 13.6 Hz) between H-2a and H-3 further confirmed that H-2a was on the different side of A-ring with H-3 and 1-OH (Figure 2B). Therefore, the absolute configuration of **3** could be either (1*S*, 3*S*, 4*S*) or (1*R*, 3*R*, 4*R*). The experimental electronic circular dichroism (ECD) spectrum of **3** matched that of the calculated ECD spectrum of (1*S*, 3*S*, 4*S*)-**3** instead of that of (1*R*, 3*R*, 4*R*)-**3** (Figure 3C). Thus, compound **3**’s absolute configuration was identified as (1*S*, 3*S*, 4*S*) and named as fluostation O.

Compound **4** was obtained as orange solid. HRESIMS analysis afforded an [M − H]**^−^** ion at *m*/*z* 357.0975, giving the molecular formula of **4** as C_19_H_18_O_7_. The comparison of ^1^H-NMR spectra of **2** and **4** showed that the resonance at *δ*_H_ 2.24 (m, H-3) in **2** disappeared in **4**, and the resonance at *δ*_H_ 1.04 (d, *J* = 7.1 Hz, H_3_-12) in **2** was replaced by *δ*_H_ 0.85 (s, H_3_-12) in **4** (Table 1). The ^13^C-NMR spectrum of **4** resembled that of **2** except that the resonance at *δ*_C_ 33.3 (C-3) in **2** was replaced by *δ*_C_ 74.2 (C-3) in **4**, the resonance at *δ*_C_ 74.0 (C-2), *δ*_C_ 69.5 (C-4), *δ*_C_ 66.2 (C-1) and *δ*_C_ 12.2 (C-12) in **2** shifted downfield to *δ*_C_ 77.4 (C-2), *δ*_C_ 73.5 (C-4), *δ*_C_ 70.2 (C-1) and *δ*_C_ 15.4 (C-12) in **4**, respectively (Table 2). These different chemical shifts and coupling patterns suggested that the only difference between **2** and **4** was that **4** contained one more hydroxy group attached at C-3. This was confirmed by the correlations from the COSY, HSQC and HMBC spectra of **4** (Figure 2A). In the ROESY spectrum of **4**, the correlation of H-12 with H-1 suggested that both H-12 and H-1 took axial conformations and were on the same side of A-ring; the correlation of H-2 with H-4 suggested that both H-2 and H-4 took axial conformations and were on the same side of A-ring; the correlation of axial H-2 with 3-OH suggested that H-2 and 3-OH were on the same side of A-ring, and H-2 and H-12 were on the different side of A-ring (Figure 2B). Therefore, the absolute configuration of **4** could be either (1*R*, 2*S*, 3*S*, 4*R*) or (1*S*, 2*R*, 3*R*, 4*S*). The experimental electronic circular dichroism (ECD) spectrum of **4** matched that of the calculated ECD spectrum of (1*R*, 2*S*, 3*S*, 4*R*)-**4** instead of that of (1*S*, 2*R*, 3*R*, 4*S*)-**4** (Figure 3D), identifying the absolute configuration of **4** as (1*R*, 2*S*, 3*S*, 4*R*). Compound **4** was successfully crystallized from the solvent of CHCl_3_:MeOH (9:1) and the crystal structure was solved based on the analysis of diffraction data obtained from Cu Kα radiation (Flack parameter = 0.00(13)), unambiguously identifying the absolute configuration of **4** as (1*R*, 2*S*, 3*S*, 4*R*) (Figure 2C). The crystal structure of **4** also confirmed that our calculated ECD experiments were reliable, and **4** was named as fluostatin P.

Compound **5** was obtained as orange solid. HRESIMS analysis afforded an [M − H]**^−^** ion at *m*/*z* 339.0865, giving the molecular formula of **5** as C_19_H_16_O_6_. The ^1^H-NMR spectrum of **5** resembled that of **4** except that the resonance at *δ*_H_ 4.77 (br s, 2-OH) and *δ*_H_ 5.10 (br s, 3-OH) in **4** disappeared in **5**, the resonance at *δ*_H_ 3.59 (dd, *J* = 6.7, 3.8 Hz, H-2) in **4** shifted upfield to *δ*_H_ 3.28 (d, *J* = 3.1 Hz, H-2) in **5**, and the resonance at *δ*_H_ 4.74 (dd, *J* = 6.7, 4.8 Hz, H-1), *δ*_H_ 4.31 (br d, *J* = 5.2 Hz, H-4), and *δ*_H_ 0.85 (s, H_3_-12) in **4** shifted downfield to *δ*_H_ 5.82 (dd, *J* = 6.0, 3.1 Hz, H-1), *δ*_H_ 4.80 (d, *J* = 8.0 Hz, H-4), and *δ*_H_ 1.47 (s, H_3_-12) in **5** (Table 1). The ^13^C-NMR spectrum of **5** resembled that of **4** except that the resonance at *δ*_C_ 77.4 (C-2), *δ*_C_ 74.2 (C-3), *δ*_C_ 70.2 (C-1) and *δ*_C_ 73.5 (C-4) in **4** shifted upfield to *δ*_C_ 59.2 (C-2), *δ*_C_ 58.2 (C-3), *δ*_C_ 59.9 (C-1) and *δ*_C_ 67.7 (C-4) in **5**, respectively, and the resonance at *δ*_C_ 15.4 (C-12) in **4** shifted downfield to *δ*_C_ 18.8 (C-12) in **5** (Table 2). These different chemical shifts and coupling patterns suggested that the only difference between **4** and **5** was that the 2,3-diol moiety in **4** was replaced by one 2,3-epoxide moiety in **5**. This was confirmed by the correlations from the COSY, HSQC and HMBC spectra of **5** (Figure 2A). In the ROESY spectrum of **5**, the correlation of H-4 with 1-OH suggested that both H-4 and 1-OH took axial conformations and were on the same side of A-ring, which should be in a stable “boot” conformation due to the 2,3-epoxide moiety; the correlation of axial H-4 with H-12, H-12 with H-2 suggested that H-4, H-12, H-2 and 1-OH were on the same side of A-ring (Figure 2B). The small coupling constant (*J* = 3.1 Hz) between H-2 and H-1, although they were on the different side of A-ring, was very similar to that (*J* = 2.0 Hz) between H-2 and H-1 in fluostatin D, whose crystallographic structure has been solved with X-ray diffraction analysis [23]. Therefore, the absolute configuration of **5** could be either (1*R*, 2*S*, 3*R*, 4*R*) or (1*S*, 2*R*, 3*S*, 4*S*). The experimental electronic circular dichroism (ECD) spectrum of **5** matched that of the calculated ECD spectrum of (1*R*, 2*S*, 3*R*, 4*R*)-**5** instead of that of (1*S*, 2*R*, 3*S*, 4*S*)-**5** (Figure 3E), identifying the absolute configuration of **5** as (1*R*, 2*S*, 3*R*, 4*R*). The absolute configuration of **5** were consistent with that of crystal structure fluostatin D, and **5** was named as fluostatin Q.

Until now, there have been 16 fluostatin analogues discovered from terrestrial and marine actinomycetes [22,23,24,25,26] or heterologous expression of environmental DNAs [27]. Compared to known fluostatins, compounds **1**–**5** contain different methoxy group attached at C-7; compounds **2**–**5** contain different hydroxy group attached at C-4; and compound **4** contains high oxidized A-ring with four hydroxy groups attached at C-1, C-2, C-3 and C-4. These new structural properties enrich the structural diversity of fluostatins, highlight marine actinomycetes as important sources of new aromatic polyketides and indicate unusual issues in their biosynthesis.

All compounds **1**–**5** were assessed for their biological activities with multiple assays. In the antibacterial activity assays against *Staphylococcus aureus* ATCC 29213, *Escherichia coli* ATCC 25922, *Pseudomonas aeruginosa* PA14 and vancomycin resistant *Enterococci faecalis* A4 (VRE), **1**–**5** all showed no activity with the minimal inhibition concentrations (MIC) larger than 25 µM. In the inhibitory assays against nitric oxide (NO) production induced by lipopolysaccharide (LPS), **1**–**5** all showed no activity with the inhibition rate below 10% at the concentration of 10 µM. In the cytotoxicity assays against human liver cancer cell line HepG2, **1**–**5** all showed no activity with the inhibition rate below 10% at the concentration of 10 µM. More biological assays are needed to reveal a potential of medical use for **1**–**5**.

### 2.3. Proposed Biosynthetic Pathways of Compounds ***1***–***5***

**Figure 4:**
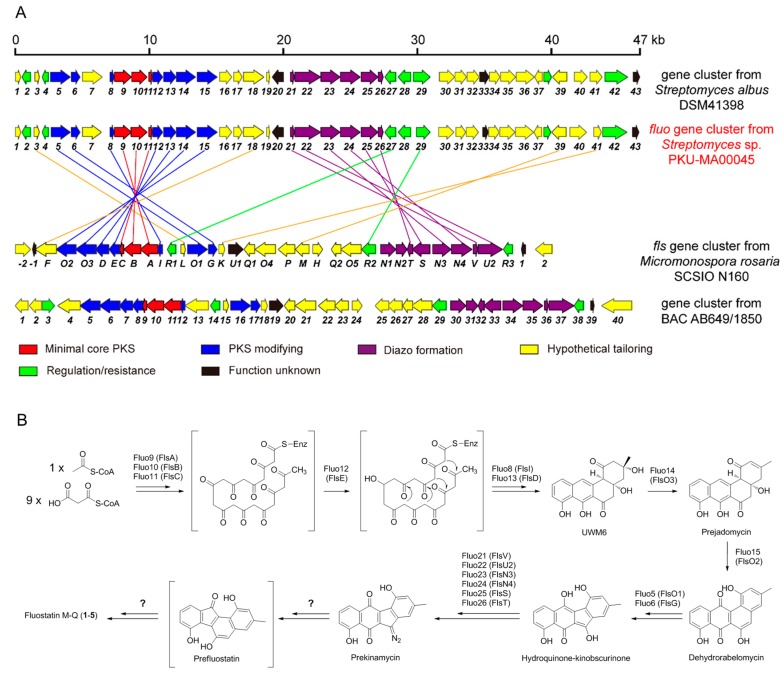
The comparison of fluostatin biosynthetic gene clusters and the proposed biosynthetic pathways of compounds **1**–**5**. (**A**) The comparison of *fluo* gene clusters from *Streptomyces* sp. PKU-MA00045 with other fluostatin biosynthetic gene clusters. The homologous genes between *fluo* and *fls* gene clusters were linked with lines. (**B**) The proposed biosynthetic pathways of compounds **1**–**5**. The protein homologues from the *fls* pathway are shown in parentheses.

Fluostatins, distinct to other angucyclinones, feature a fluorenone chromophore with a unique 6-5-6-6 carbon ring skeleton (Figure 1). The formation of the five-membered C-ring of fluostatins is most fascinating in the biosynthetic research, and several reports have proposed that the 6-5-6-6 ring skeleton of fluostatins were from the rearrangement of diazo-containing 6-6-5-6 ring skeleton [26,28,29], exemplified by prekinamycin and prelomaiviticin in the kinamycin and lomaiviticin biosynthesis, respectively [28,29]. Two fluostatin biosynthetic gene clusters, the *fls* from marine *Micromonospora rosaria* SCSIO N160 [25] and the other from environmental DNAs [27], have been reported. They showed high homology with each other and their gene organizations were almost identical (Figure 4A). The heterologous expression of the *fls* gene cluster and gene inactivations have identified the cluster’s boundary and confirmed the cluster is intact [25]. However, the biosynthesis of the five-membered C-ring of fluostatins still remain elusive. We sequenced the genomic DNA of PKU-MA00045 and identified the putative biosynthetic gene cluster (named as *fluo*, boundary not identified) of **1**–**5**, based on the searching of homologues in the published fluostatin gene clusters. Surprisingly, the *fluo* gene cluster contained different genes and gave different gene organizations compared to the above two published ones. The homologues of genes of *flsU1, flsQ1, flsO4, flsP, flsH, flsQ2, flsO5, flsN1, flsN2* and *flsR3* in the *fls* gene cluster were absent in the *fluo* gene cluster; the minimal core PKS genes, *flsA-C* (encoding KS_α_, KS_β_ and ACP), together with surrounding genes in the *fls* gene cluster showed different transcription directions and different relative positions compared to their homologues in the *fluo* gene cluster; the homologues of six genes *flsV, flsU2, flsN3, flsN4, flsS* and *flsT* in the *fls* gene cluster, which are the conserved genes proposed for the diazo assembly in kinamycin, lomaiviticin and fluostatin biosynthesis [25,28,29], also presented in the *fluo* gene cluster but showed different transcription directions and relative positions (Figure 4A, Table S2). When we searched the homologues of genes in the *fluo* gene cluster by BLAST, we found that many genes showed high sequence identities to homologues from one gene cluster in the genome of *Streptomyces albus* DSM41398. A further comparison revealed that the *fluo* gene cluster contained almost the same genes and gene organizations as the gene cluster from *Streptomyces albus* DSM41398 (Figure 4A), suggesting that *Streptomyces albus* DSM41398 is another potential fluostatin producer. The two published fluostatin gene clusters are from environmental DNAs and *Micromonospora rosaria* SCSIO N160, and the *fluo* gene cluster and its close homology are both from *Streptomyces*, suggesting that the different genera of strains may account for the differences between fluostatin biosynthetic gene clusters.

Based on known biosynthetic research, we proposed the biosynthetic pathways of compounds **1**–**5**. The type II PKS, Fluo9-11, catalyzed the iterative linear polyketide chain elongation using one molecule of acetyl-CoA as the starter unit and nine molecules of malonyl-CoA as the extender units [12,13,25,28]; the ketoacyl reductase Fluo12 and cyclases Fluo8 and Fluo13, catalyzed the reduction and cyclization reactions to generate UWM6, which is a common intermediate in angucyclinone biosynthesis [13,25,28]; the dehydratase Fluo14 converted the UWM6 to prejadomycin, which was oxidized to generate dehydrorabelomycin by the oxygenase Fluo15 [25,28]; the dehydrorabelomycin was converted to hydroquinone-kinobscurinone by the two associated enzymes Fluo5 and Fluo6, whose homologues AlpK and AlpJ have been confirmed to catalyze the same reactions in kinamycin biosynthesis [28,30]; the six associated enzymes Fluo21-26 converted hydroquinone-kinobscurinone to the diazo-containing prekinamycin [30], which underwent unknown rearrangement steps to generate prefluostatin with the unique 6-5-6-6 carbon ring skeleton [25,28,29]; then the prefluostatin underwent multiple tailoring steps including methylation and oxidation to generate **1**–**5** (Figure 4B). In the tailoring steps converting prefluostatin to **1**–**5**, the methyltransferase Fluo39 and hydrolase Fluo34 may participate in the methylation of the 7-hydroxy group and the hydrolyzation of the 2,3-epoxide ring, respectively. In the rearrangement converting prekinamycin to prefluostatin, no candidate could be identified only based on the bioinformatics analysis and comparison between the gene clusters. However, the absence of homologues of *flsU1, flsQ1, flsO4, flsP, flsH, flsQ2, flsO5, flsN1, flsN2* and *flsR3* in the *fluo* gene cluster suggests that these genes may not participate in this key rearrangement. The sequencing of the *fluo* gene cluster and the comparative analyses between the fluostatin biosynthetic gene clusters in this study set the stage for future characterizations of the functions of other genes in the *fls* and *fluo* gene clusters, to fully understand the formation of the 6-5-6-6 carbon ring skeleton and the tailoring modifications.

## 3. Materials and Methods

### 3.1. General Experimental Procedures

Optical rotations were measured on an Autopol III automatic polarimeter (Rudolph Research Analytical, Hackettstown, NJ, USA). UV spectra were collected on a Cary 300 spectrometer (VARIAN, Palo Alto, CA, USA). IR spectra were collected on a Nicolet Nexus 470 FT-IR spectrometer (Thermo Scientific, Waltham, MA, USA). CD spectra were collected on a J-810 spectropolarimeter (Jasco Corporation, Tokyo, Japan). ^1^H and ^13^C-NMR spectra were collected on a Bruker Avance-600 NMR spectrometer (Bruker Corporation, Billerica, MA, USA). HRESIMS spectra were collected on a Waters Xevo G2 Q-TOF spectrometer (Waters, Milford, MA, USA). HPLC analysis was performed on an Agilent 1260 series (Agilent Technologies, Santa Clara, CA, USA) with a C_18_ RP-column (Eclipse XDBC_18_, 150 × 4.6 mm, 5 μm, Agilent Technologies, Santa Clara, CA, USA). Semi-preparative HPLC was performed on a SSI 23201 system (Scientific Systems Inc., State College, PA, USA) with a YMC-Pack ODS-A column (250 × 10 mm, 5 μm, YMC CO., LTD. Shimogyo-ku, Kyoto, Japan). MPLC was performed on a LC3000 series (Beijing Tong Heng Innovation Technology, Beijing, China) with a Claricep^TM^ Flash i-series C_18_ cartridge (20–35 μm, 40 g, Bonna-Agela, Wilmington, DE, USA). Size exclusion chromatography was carried out using a Sephadex LH-20 (GE Healthcare, Chicago, IL, USA) column.

### 3.2. PCR Screening for Potential Producers of Angucyclinones from a Marine Bacteria Collection

We have previously reported the isolation of 180 marine bacteria from 15 sponge samples that were collected from South China Sea [19]. We have extracted all strains’ genomic DNAs and discovered nonribosomal peptides (bacillibactin and bacillomycin D analogues) by using a PCR screening method [19]. In this study, the forward primer (5′-GGCAGCGGITTCGGCGGITTCCAG-3′) and the reverse primer (5′-CGITGTTIACIGCGTAGAACCAGGCG-3′), designed from the conserved sequences of KS_α_ and KS_β_ in the biosynthesis of tetracenomycin, daunorubicin, actinorhodin and fredericamycin [20], were used in the PCR screening for aromatic polyketide producers. A 20 μL PCR system consisting of 10 µL Easy Taq Polymerase (Beijing TransGen Biotech, Beijing, China), 2 µL of forward and reverse primer mixture (each for 10 µM), 1 µL genomic DNA (50–100 ng), and 7 µL sterilized water, were used. The PCR program was performed with an initial denaturation at 95 °C for 5 min, followed by 30 cycles of denaturation at 95 °C for 30 s, annealing at 60 °C for 1 min and extension at 72 °C for 1 min, followed by incubation at 72 °C for 10 min. In total, 167 strains were screened and the PCR products were analyzed by agarose gel electrophoresis, recovered with a gel purification kit (Beijing TransGen Biotech, Beijing, China) and sequenced to afford 12 “positive” strains.

### 3.3. Small-Scale Fermentation and HPLC Analysis

For each of the 12 “positive” strains, 50 µL of spore suspension was inoculated into 50 mL of seed medium M1 (yeast extract 1 g, peptone 5 g, beef extract 1 g, FePO_4_ 0.01 g, and sea salt 33 g in 1.0 L distilled H_2_O, pH 7.4), and incubated with a HYG-C shaker (Suzhou Peiying Laboratory Equipment, Suzhou, China) at 28 °C, 200 rpm for three days. Two milliliters of the resultant seed culture was inoculated into 50 mL of production media, and the fermentation continued at 28 °C, 200 rpm for seven days. Four different production media, M1, M2 (glycerol 6 mL, arginine 1 g, K_2_HPO_4_ 1 g, MgSO_4_ 0.5 g, sea salt 33 g in 1.0 L distilled H_2_O, pH 7.2), M3 (soluble starch 20 g, KNO_3_ 1 g, K_2_HPO_4_ 0.5 g, MgSO_4_·7H_2_O 0.5 g, NaCl 0.5 g, FeSO_4_·7H_2_O 0.01 g, sea salt 33 g in 1.0 L distilled H_2_O, pH 7.2) and M4 (soluble starch 10 g, casein 0.3 g, K_2_HPO_4_ 2 g, KNO_3_ 2 g, MgSO_4_·7H_2_O 0.05 g, NaCl 2 g, FeSO_4_·7H_2_O 0.01 g, CaCO_3_ 0.02 g, sea salt 33 g in 1.0 L distilled H_2_O, pH 7.2), were used in the small-scale fermentation. The Diaion HP20 (2 g/100 mL, Mitsubishi Chemical Corporation, Tokyo, Japan) and Amberlite XAD-16 (2 g/100 mL, Sigma-Aldrich, St. Louis, MO, USA) resins were added 10 h before the fermentation finished. The resins and cell mass were harvested by centrifugation, washed with distilled H_2_O and extracted with MeOH. The MeOH extracts were concentrated and analyzed by HPLC. The HPLC analysis was carried out with a flow rate of 1 mL/min with UV detection at 254 nm, using a gradient elution program from 5% MeOH in H_2_O to 100% MeOH over 45 min.

### 3.4. Phylogenetic Analysis

The phylogenetic analysis of strains PKU-MA00045 (isolated from a sponge *Sinularia* species) was carried out by sequence alignments of its 16S rRNAs (GeneBank accession number MF435987) with different *Streptomyces* homologues. The forward primer (5′-AGAGTTTGATCMTGGCTCAG-3′) and reverse primer (5′-TACGGYTACCTTGTTACGACTT-3′) [31] were used to amplify the 16S rDNA genes. Homologous genes were searched using BLAST on the NCBI website and the phylogenetic tree was generated with Mega 7.0 (Pennsylvania State University, State College, PA, USA) using the Neighbor-Joining algorithm.

### 3.5. Large-Scale Fermentation and Isolation

The large-scale fermentation of *Streptomyces* sp. PKU-MA00045 in medium M4 were carried out with similar procedures used in the small-scale fermentation. Briefly, 50 µL of spore suspension was inoculated into 50 mL of seed medium (medium M1) and incubated with HYG-C shakers at 28 °C, 200 rpm for three days. Eight milliliters of the resultant seed culture was inoculated into 200 mL of medium M4 in 1 L Erlenmeyer flasks, and the fermentation continued at 28 °C, 200 rpm for seven days. The Diaion HP20 (2 g/100 mL), and Amberlite XAD-16 (2 g/100 mL) resins were added 10 h before the fermentation finished. The resins and cell mass were harvested by centrifugation, washed with distilled H_2_O and extracted with MeOH.

A 15 L fermentation of *Streptomyces* sp.PKU-MA00045 gave a 4.5 g of MeOH extract. The MeOH extract was concentrated, resuspended in H_2_O and extracted with EtOAc three times. The EtOAc extract (2.0 g) was loaded onto MPLC with an elution program of step-gradient of MeOH in H_2_O (30%, 65%, 95%) to yield nine fractions (F1–F9), at the flowrate of 17 mL/min and under the detection of 254 nm. Fraction F7 was purified by semi-preparative HPLC with MeOH/H_2_O (49/51, *v*/*v*) as the mobile phase, to afford compounds **1** (2.8 mg), **2** (1.0 mg), **4** (4.8 mg) and **5** (0.7 mg); fraction F8 was purified by semi-preparative HPLC with MeOH/H_2_O (59/41, *v*/*v*) as the mobile phase to yield compound **3** (0.8 mg). All semi-preparative HPLCs were carried out at a flowrate of 2 mL/min and under the detection of 260 nm.

Fluostatin M (**1**): orange solid; ${\left[\alpha \right]}_{D}^{25}$ −9.3 (*c* 0.3, MeOH); UV (MeOH) *λ*_max_ (log *ε*) 269 (3.93), 297 (3.68), 438 (3.16) nm; IR (KBr) *ν*_max_ 3443, 2954, 2928, 1250, 836 cm^−1^; ^1^H and ^13^C-NMR data, see Table 1 and Table 2; HRESIMS *m*/*z* 339.0870 [M − H]**^−^** (calcd. for C_19_H_15_O_6_, 339.0869).

Fluostatin N (**2**): orange solid; ${\left[\alpha \right]}_{D}^{25}$ +24 (*c* 0.1, MeOH); UV (MeOH) *λ*_max_ (log *ε*) 218 (3.97), 246 (3.86), 261 (3.86), 272 (3.79), 340 (3.01), 445 (3.01) nm; IR (KBr) *ν*_max_ 3443, 2918, 1384, 1244, 1051,1033 cm^−1^; ^1^H and ^13^C-NMR data, see Table 1 and Table 2; HRESIMS *m*/*z* 341.1023 [M − H]**^−^** (calcd. for C_19_H_17_O_6_, 341.1025).

Fluostatin O (**3**): orange solid; ${\left[\alpha \right]}_{D}^{20}$ +39 (*c* 0.04, MeOH); UV (MeOH) *λ*_max_ (log *ε*) 217 (3.78), 246 (3.70), 260 (3.72), 272 (3.64), 340 (2.91), 445 (2.68) nm; IR (KBr) *ν*_max_ 3419, 2922, 1692, 1605, 1383, 1245, 1051, 1033, 1017 cm^−1^; ^1^H and ^13^C NMR data, see Table 1 and Table 2; HRESIMS *m*/*z* 325.1084 [M − H]**^−^** (calcd. for C_19_H_17_O_5_, 325.1076).

Fluostatin P (**4**): orange solid; ${\left[\alpha \right]}_{D}^{25}$ +8.9 (*c* 0.3, MeOH); UV (MeOH) *λ*_max_ (log *ε*) 218 (3.89), 246 (3.76), 261 (3.76), 273 (3.72), 340 (3.04), 445 (2.77) nm; IR (KBr) *ν*_max_ 3421, 2930, 1722, 1095 cm^−1^; ^1^H and ^13^C NMR data, see Table 1 and Table 2; HRESIMS *m*/*z* 357.0975 [M − H]**^−^** (calcd. for C_19_H_17_O_7_, 357.0974).

Fluostatin Q (**5**): orange solid; ${\left[\alpha \right]}_{D}^{25}$ +14 (*c* 0.07, MeOH); UV (MeOH) *λ*_max_ (log *ε*) 218 (3.90), 245 (3.79), 261 (3.80), 272 (3.76), 340 (3.01), 449 (2.77) nm; IR (KBr) *ν*_max_ 3429, 2926, 2856, 1662, 1032 cm^−1^; ^1^H and ^13^C-NMR data, see Table 1 and Table 2; HRESIMS *m*/*z* 339.0865 [M − H]**^−^** (calcd. for C_19_H_15_O_6_, 339.0869).

### 3.6. ECD Calculation of ***1***–***5***

Conformational analysis of the enantiomers of **1**–**5** established by ROESY analyses were carried out via Monte Carlo searching with the MMFF94s molecular mechanics force field using the Spartan 10 software (Wavefunction Inc., Irvine, CA, USA). Compounds **1**–**5** gave 3, 5, 4, 4, 3 geometries, respectively, which possessed relative energies within 10 kcal/mol. These geometries were optimized by DFT at the B3LYP/6-31G (d) level (methanol as the solvent) with the Gaussian 09 program (Gaussian Inc., Wallingford, CT, USA). The B3LYP/6-31G(d)-optimized conformers were then optimized at the *w*B97XD/DGDZVP level (methanol as the solvent). ECD computations for all *w*B97XD/DGDZVP-optimized conformers were carried out at the CAM-B3LYP/DGDZVP level (methanol as the solvent). Boltzmann statistics were performed for ECD simulations with a standard deviation of σ 0.3 eV. Then, the ECD spectra were simulated by the Gaussum 2.25 program [32] and generated according to the Boltzmann distribution theory and their relative Gibbs free energy (ΔG).

### 3.7. X-ray Crystallographic Analysis of Compound ***4***

Orange crystals of compound **4** were obtained from the solvent of CHCl_3_:MeOH (9:1). The X-ray diffraction data were collected on a MicroMax-003 HomeLab CCD X-ray single crystal diffractometer (Rigaku Americas Corporation, The Woodlands, TX, USA) at the State Key Laboratory of Natural and Biomimetic Drugs, Peking University, with Cu K*α* radiation (*λ* = 1.54184 Å) used. The structure was solved by direct methods (SHELXT-14) and refined using full-matrix least-squares difference Fourier techniques. Crystallographic data have been deposited in the Cambridge Crystallographic Data Center with the deposition number CCDC 1815221.

## 4. Conclusions

The marine environments characterized by high salinity, high pressure and low temperature hold the promise of producing new natural products structurally or bioactively distinct from those from terrestrial environments. In this study, we discovered five new fluostatin analogues (**1**–**5**) from marine *Streptomyces* sp. PKU-MA00045 with a PCR-based genome mining method. These atypical angucyclinones featured a 6-5-6-6 ring skeleton and high oxidized A-rings, highlighting marine actinomycetes as important producers of new aromatic polyketides. The biosynthetic gene cluster of compounds **1**–**5** showed distinct gene contents and gene organizations with two known fluostatin biosynthetic gene clusters, facilitating the full characterization of the 6-5-6-6 carbon ring skeleton formation and the tailoring modifications. These results have inspired our further effort on new marine natural products discovery and related biosynthetic research.

## Figures and Tables

**Table 1:** The ^1^H-NMR (600 MHz) data (*J* in Hz) of compounds **1**–**5** in DMSO-*d*_6._

Position	1	2	3	4	5
1	5.46, d (3.5) ^a^	5.12, t (4.3)	5.30, m	4.74, dd (6.7, 4.8)	5.82, dd (6.0, 3.1)
2	4.01, dd (3.5, 2.3) ^a^	3.75, td (4.5, 2.3)	1.86, td (13.6, 4.0)	3.59, dd (6.7, 3.8)	3.28, d (3.1)
			1.52, dt (13.6, 2.5)		
3	3.17, qd (6.8, 2.3) ^a^	2.24, m	2.19, m		
4		4.41, dd (7.8, 4.0)	4.30, t (4.0)	4.31, br d (5.2)	4.80, d (8.0)
5	7.40, s	7.00, s	6.93, s	7.09, d (1.0)	7.17, d (1.0)
8	7.45, dd (8.3, 1.4)	7.41, dd (8.2, 1.3)	7.40, dd (8.3, 1.3)	7.40, dd (8.3, 1.0)	7.40, dd (8.3, 1.2)
9	7.47, dd (8.3, 6.6)	7.38, dd (8.2, 6.8)	7.37, dd (8.3, 6.7)	7.37, dd (8.3, 7.0)	7.37, dd (8.3, 6.8)
10	7.36, dd (6.6, 1.4)	7.29, dd (6.8, 1.3)	7.28, dd (6.7, 1.3)	7.29, dd (7.0, 1.0)	7.27, dd (6.8, 1.2)
12	1.17, d (6.9) ^a^	1.04, d (7.1)	0.98, d (7.0)	0.85, s	1.47, s
13	4.11, s	4.10, s	4.10, s	4.08, s	4.09, s
1-OH	5.46, overlap	4.96, d (4.4)	4.53, d (3.7)	5.08, d (4.8)	5.31, d (6.1)
2-OH	5.31, d (3.5)	5.19, d (4.8)		4.77, br s	
3-OH				5.10, br s	
4-OH		4.81, d (8.0)	4.94, d (5.8)	5.44, d (5.8)	5.93, d (8.2)
6-OH	9.54, s	9.32, s	9.30, s	9.33, s	9.33, s

^a^ The splitting style and coupling constants were listed based on the ^1^H-NMR spectrum with D_2_O added.

**Table 2:** The ^13^C-NMR (150 MHz) data of compounds **1**−**5** in DMSO-*d*_6._

Position	1	2	3	4	5
	*δ*c, Type	*δ*c, Type	*δ*c*,* Type	*δ*c, Type	*δ*c, Type
1	63.6, CH	66.2, CH	60.4, CH	70.2, CH	59.9, CH
2	74.8, CH	74.0, CH	33.0, CH_2_	77.4, CH	59.2, CH
3	41.6, CH	33.3, CH	28.1, CH	74.2, C	58.2, C
4	197.7, C	69.5, CH	68.7, CH	73.5, CH	67.7, CH
4a	134.0, C	143.9, C	144.2, C	143.6, C	142.6, C
5	119.8, CH	124.4, CH	125.1, CH	122.5, CH	121.6, CH
6	150.9, C	150.2, C	150.0, C	150.4, C	150.5, C
6a	132.5, C	126.7, C	126.2, C	126.2,C	125.1, C
6b	127.9, C	128.9, C	128.9, C	129.1, C	128.9, C
7	151.8, C	151.0, C	151.0, C	151.1, C	151.0, C
8	120.5, CH	119.6, CH	119.4, CH	120.0, CH	119.5, CH
9	131.8, CH	130.6, CH	130.5, CH	130.7, CH	130.5, CH
10	117.7, CH	117.7, CH	117.6, CH	118.0, CH	117.6, CH
10a	135.3, C	134.7, C	134.8, C,	134.5, C	134.7, C
11	192.1, C	193.4, C	193.2, C	194.4, C	193.0, C
11a	131.6, C	132.0, C	131.1, C	130.8, C	130.6, C
11b	134.7, C	129.8, C	131.5, C	131.7, C	128.3, C
12	11.3, CH_3_	12.2, CH_3_	17.2, CH_3_	15.4, CH_3_	18.8, CH_3_
13	57.5, CH_3_	57.4, CH_3_	57.4, CH_3_	57.4, CH_3_	57.4, CH_3_

## Supplementary Materials

The following are available online at http://www.mdpi.com/1660-3397/16/3/87/s1, Table S1: The 12 “positive” strains and the homologues of their PCR products, Table S2: The deduced functions of genes from the *fluo* gene cluster and their homologues from the *fls* gene cluster, Figure S1: The representative new aromatic polyketides discovered from marine actinomycetes, Figure S2: The agarose gel electrophoresis analysis of the 12 “positive” PCR products, Figure S3: the phylogenetic analysis of strain PKU-MA00045, Figures S4–S13: The NMR, MS and IR spectra of compound **1**, Figures S14–S21: the NMR, MS and IR spectra of compound **2**, Figures S22–S29: the NMR, MS and IR spectra of compound **3**, Figures S30–S37: the NMR, MS and IR spectra of compound **4**, Figures S38–S45: the NMR, MS and IR spectra of compound **5**.
