# Supplementary material for: Fluostatins M–Q Featuring a 6-5-6-6 Ring Skeleton and High Oxidized A-Rings from Marine Streptomyces sp. PKU-MA00045
Source: Mar Drugs. 2018 Mar 9;16(3):87. doi: 10.3390/md16030087 (PMC5867631; doi:10.3390/md16030087)

Fluostatins M-Q Featuring a 6-5-6-6 Ring Skeleton and High Oxidized A-rings from Marine *Streptomyces* sp. PKU-MA00045

Jing Jin [1], Xiaoyan Yang [1], Tan Liu [1], Hua Xiao [1], Guiyang Wang [1], Mengjie Zhou [1], Fawang Liu [1], Yingtao Zhang [1], Dong Liu [1], Minghua Chen [2], Wei Cheng [1], Donghui Yang [1,*] and Ming Ma [1,*]

[1] State Key Laboratory of Natural and Biomimetic Drugs, Department of Natural Medicines, School of Pharmaceutical Sciences, Peking University, 38 Xueyuan Road, Haidian District, Beijing 100191, China;

[2] Institute of Medicinal Biotechnology, Chinese Academy of Medical Sciences and Peking Union Medical College, 1# Tiantanxili, Beijing 100050, China

Jing Jin, Xiaoyan Yang and Tan Liu contributed equally

*Correspondence: ydhui@bjmu.edu.cn; mma@bjmu.edu.cn

Supplementary Materials

Table S1. The 12 "positive" strains and the homologues of their PCR products. S3

Table S2. The deduced functions of genes from the *fluo* gene cluster and their homologues from the *fls* gene cluster. S4-5

Figure S1. The representative new aromatic polyketides discovered from marine actinomycetes. S6

Figure S2. The agarose gel electrophoresis analysis of the 12 "positive" PCR products. S7

Figure S3. The phylogenetic analysis of strain PKU-MA00045. S8

Figure S4-13. The ^{1}H NMR, ^{1}H NMR (D_2O added), APT, COSY, HSQC, HMBC, ROESY, ROESY (D_2O added), MS, and IR spectra of compound **1**. S9-18

Figure S14-21. The ^{1}H NMR, ^{13}C NMR, COSY, HSQC, HMBC, ROESY, MS, and IR spectra of compound **2**. S19-26

Figure S22-29. The ^{1}H NMR, ^{13}C NMR, COSY, HSQC, HMBC, ROESY, MS, and IR spectra of compound **3**. S27-34

Figure S30-37. The ^{1}H NMR, APT, COSY, HSQC, HMBC, ROESY, MS, and IR spectra of compound **4**. S35-42

Figure S38-45. The ^{1}H NMR, ^{13}C NMR, COSY, HSQC, HMBC, ROESY, MS, and IR spectra of compound **5**. S43-50

Table S1. The 12 "positive" strains and the homologues of their PCR products. The right two columns show the identities and accession numbers of the highest homologues of the PCR products.

Positive strains	Homologues of PCR products	Identities	Accession numbers of the homologues
PKU-MA00045	beta-ketoacyl synthase	97%	WP_040253445
PKU-MA00046	polyketide beta-ketoacyl synthase	98%	WP_037760056
PKU-MA00115	beta-ketoacyl synthase	84%	WP_030349268
PKU-MA00146	polyketide beta-ketoacyl synthase	85%	WP_017237281
PKU-MA00159	type II polyketide synthase KSII	70%	AAZ42180
PKU-MA00174	beta-ketoacyl synthase	74%	WP_026237418
PKU-MA00175	beta-ketoacyl synthase	84%	WP_030349268
PKU-MA00181	polyketide beta-ketoacyl synthase	97%	WP_013155268
PKU-MA00192	actinorhodinpolyketide beta-ketoacylsynthase	97%	WP_018806194
PKU-MA00208	beta-ketoacyl synthase	66%	WP_030028306
PKU-MA00218	beta-ketoacyl synthase	84%	WP_030349268
PKU-MA00219	beta-ketoacyl synthase	100%	WP_029127731

Tabel S2. The deduced functions of genes from the *fluo* gene cluster and their homologues from the *fls* gene cluster.

Genes	Annotation based on BLAST				Homologues from *fls* gene cluster	
	Numbers of amino acids	Proposed functions	Accession numbers of closest homologues	Identities with homologues	Homologues	Identities
fluo1	155	ATP-binding protein	WP_052482552.1	100%		
fluo2	202	TetR/AcrR family transcriptional regulator	WP_052482553.1	100%		
fluo3	138	TIGR03618 family F420-dependent PPOX class oxidoreductase	WP_040258142.1	100%	*flsL*	61.59%
fluo4	149	nuclear transport factor 2 family protein	WP_040253434.1	99%		
fluo5	471	monooxygenase	WP_040253436.1	99%	*flsO1*	61.17%
fluo6	229	Anthrone monooxygenase	WP_040253438.1	99%	*flsG*	54.01%
fluo7	495	bilirubin oxidase	WP_054238927.1	60%		
fluo8	107	TcmI family type II polyketide cyclase	WP_040253440.1	99%	*flsI*	85.32%
fluo9	423	ketosynthase	BAA92280.1	100%	*flsA*	81.56%
fluo10	404	ketosynthase chain-length factor	WP_040253445.1	100%	*flsB*	67.48%
fluo11	89	acyl carrier protein	WP_040253447.1	100%	*flsC*	56.04%
fluo12	264	KR domain-containing protein	WP_040253449.1	100%	*flsE*	78.41%
fluo13	313	cyclase	WP_040253451.1	100%	*flsD*	64.15%
fluo14	485	oxidoreductase	WP_040253452.1	99%	*flsO3*	58.76%
fluo15	506	monooxygenase	WP_040253455.1	99%	*flsO2*	65.75%
fluo16	313	thioesterase	WP_052482554.1	99%		
fluo17	224	JadM phosphopantetheinyl transferase-like protein	AJE85506.1	99%		
fluo18	524	acyl-CoA carboxylase subunit beta	WP_052482556.1	99%	*flsF*	78.82%
fluo19	96	acyl-CoA carboxylase subunit epsilon	WP_052482557.1	95%		

fluo20	261	hypothetical protein SLNHY_5121	AOU79812.1	100%		
fluo21	111	4Fe-4S ferredoxin	WP_040253458.1	100%	*flsV*	54.69%
fluo22	639	hypothetical protein	WP_040253460.1	99%	*flsU2*	57.43%
fluo23	479	amidase	WP_078845181.1	99%	*flsN3*	63.05%
fluo24	503	glutamine synthetase	WP_040253462.1	99%	*flsN4*	63.35%
fluo25	428	adenylosuccinate lyase	WP_040253465.1	99%	*flsS*	73.60%
fluo26	134	N-acetyltransferase	WP_040253467.1	99%	*flsT*	64.93%
fluo27	261	DNA-binding response regulator	WP_078845182.1	100%	*flsR1*	51.52%
fluo28	298	DNA-directed RNA polymerase sigma-70 factor	WP_040253470.1	99%		
fluo29	339	transcriptional regulator protein	AJE85519.1	99%	*flsR2*	41.55%
fluo30	388	myo-inositol-1-phosphate synthase	WP_052482559.1	99%		
fluo31	256	prenyltransferase UbiA	WP_040253473.1	99%		
fluo32	315	xylose isomerase	WP_078845184.1	99%		
fluo33	139	hypothetical protein	WP_052483226.1	100%		
fluo34	282	hydrolase TatD	WP_040253475.1	99%		
fluo35	389	xylose isomerase	WP_040253477.1	99%		
fluo36	466	alkaline phosphatase family protein	WP_040253479.1	99%		
fluo37	184	GNAT family N-acetyltransferase	WP_040253481.1	98%		
fluo38	195	TetR family transcriptional regulator	WP_052482560.1	98%		
fluo39	362	hydroxyneurosporene methyltransferase	WP_078845185.1	99%	*flsM*	53.59%
fluo40	433	ScyD/ScyE family protein	WP_040253483.1	99%		
fluo41	194	cupin domain-containing protein	WP_078845186.1	100%	*flsK*	61.34%
fluo42	620	drug resistance transporter EmrB/QacA subfamily	AJE85534.1	99%		
fluo43	161	hypothetical protein	WP_040253488.1	100%		

Figure S1. The representative new aromatic polyketides discovered from marine actinomycetes.

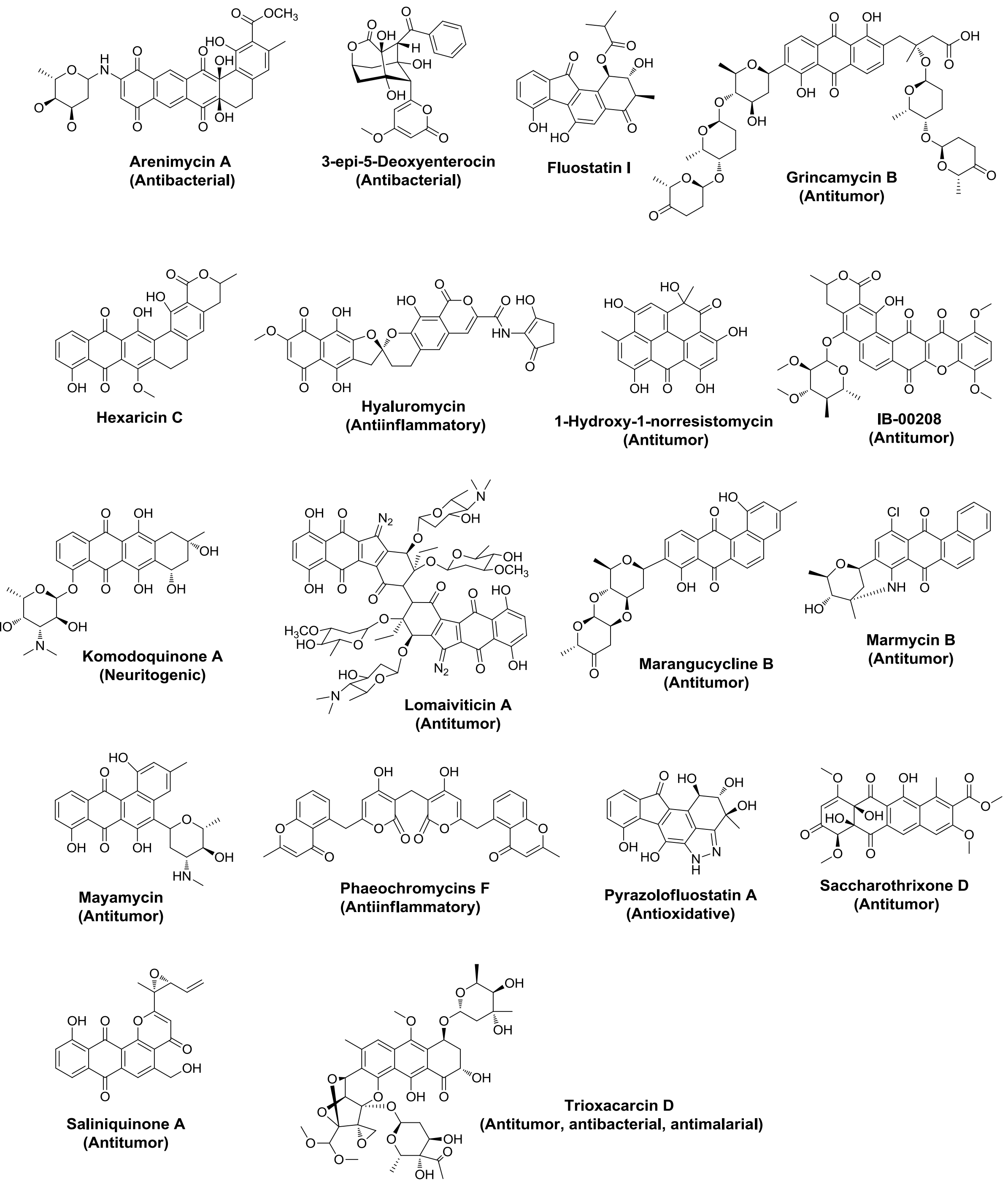

Figure S2. The agarose gel electrophoresis analysis of the 12 “positive” PCR products. The bands for expected PCR products were highlighted with a red box.

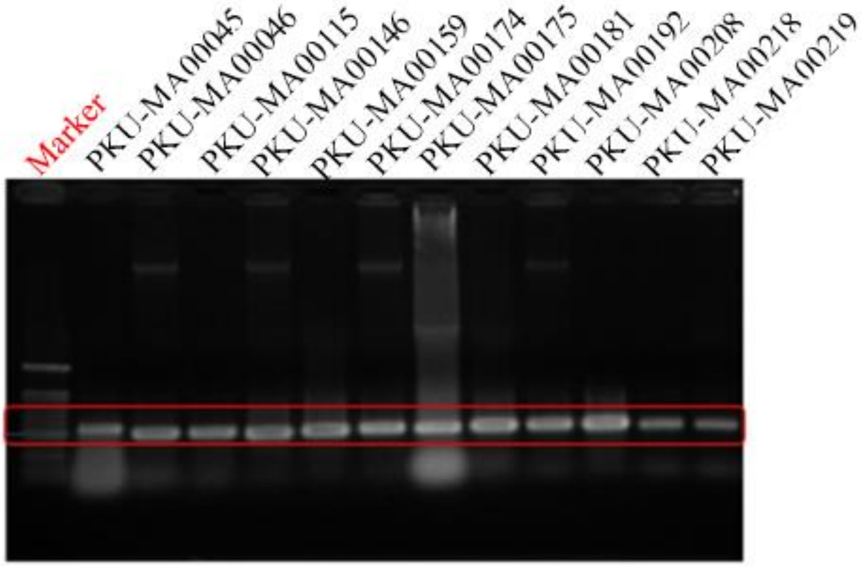

Figure S3. The phylogenetic analysis of strain PKU-MA00045 (labeled in red) based on comparison of 16S rRNA sequences. The sequence of 16S rRNA of *Streptacidiphilus hamsterleyensis* HSCA14 was used as an outgroup. The GenBank accession numbers are shown in parentheses.

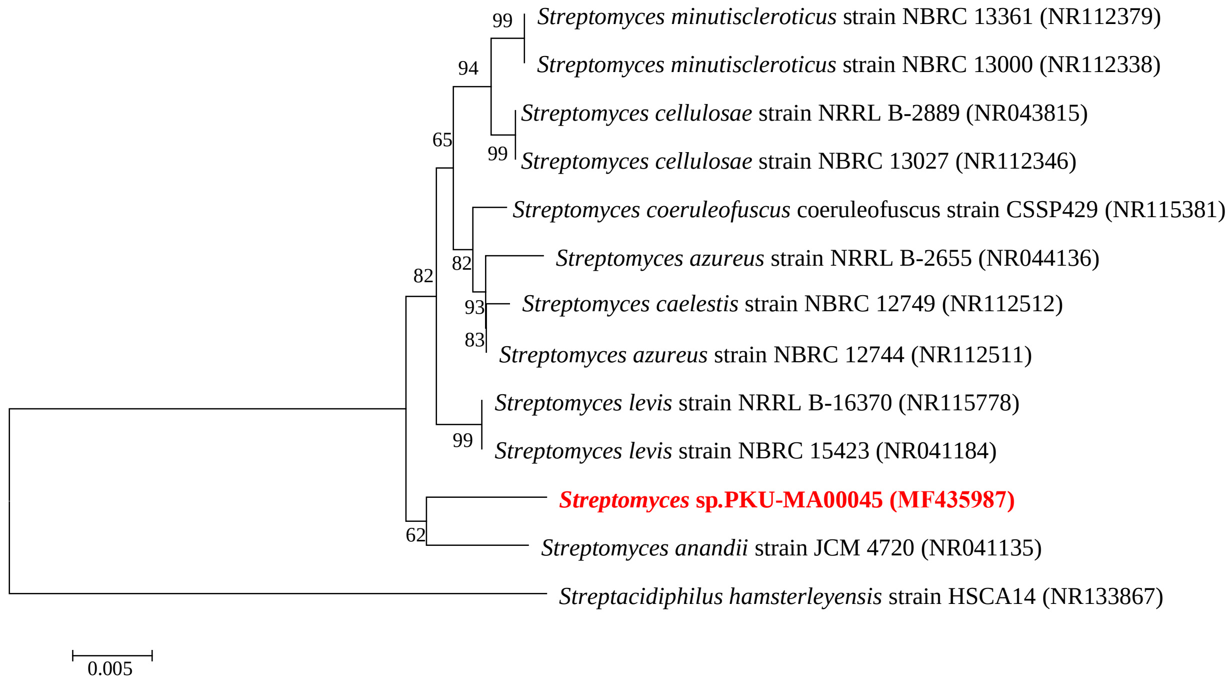

Figure S4. The ^{1}H NMR (600 MHz) spectrum of compound **1** in DMSO-d_6

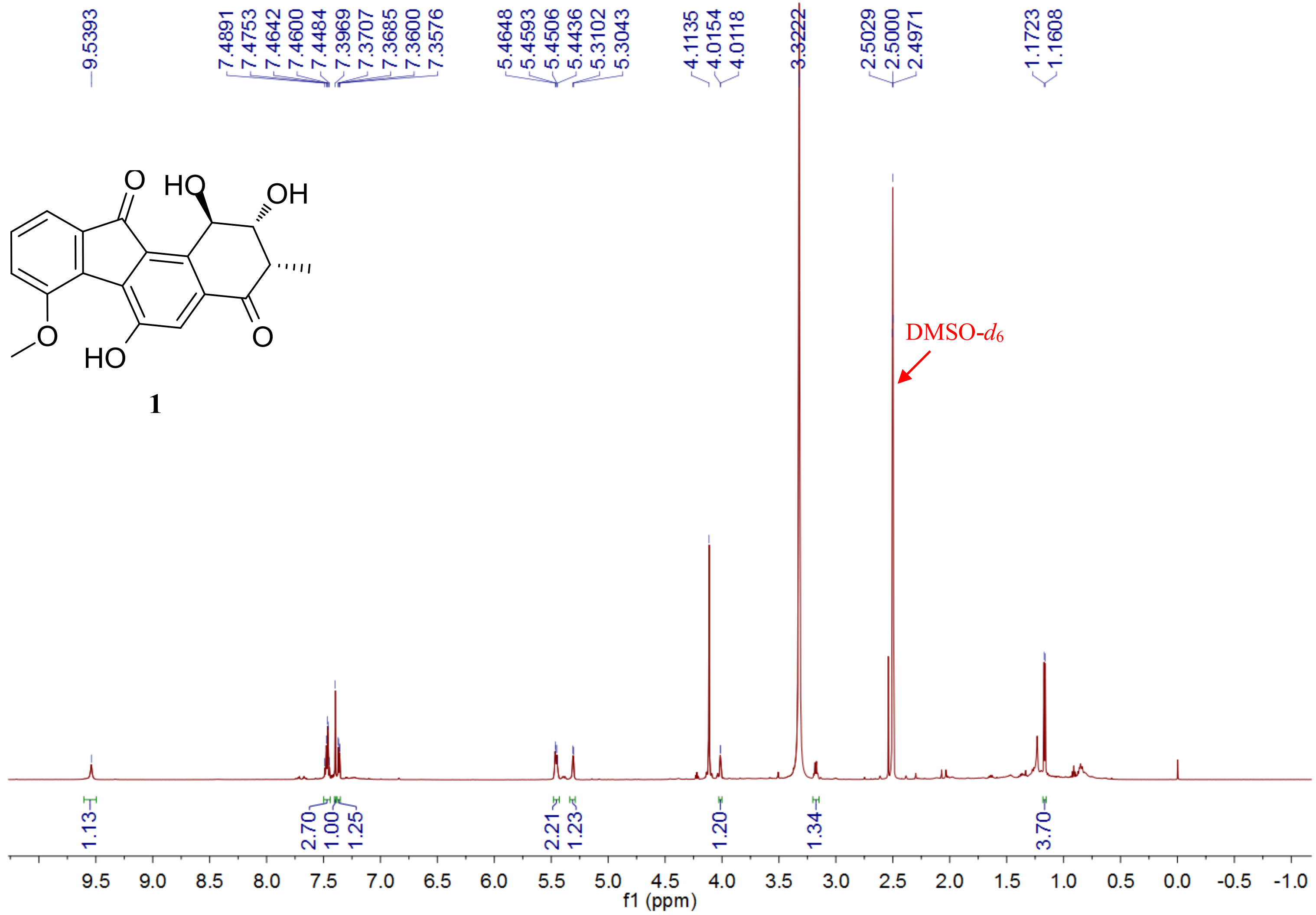

Figure S5. The ^{1}H NMR (600 MHz) spectrum of compound **1** in DMSO-d_6 (D_2O added)

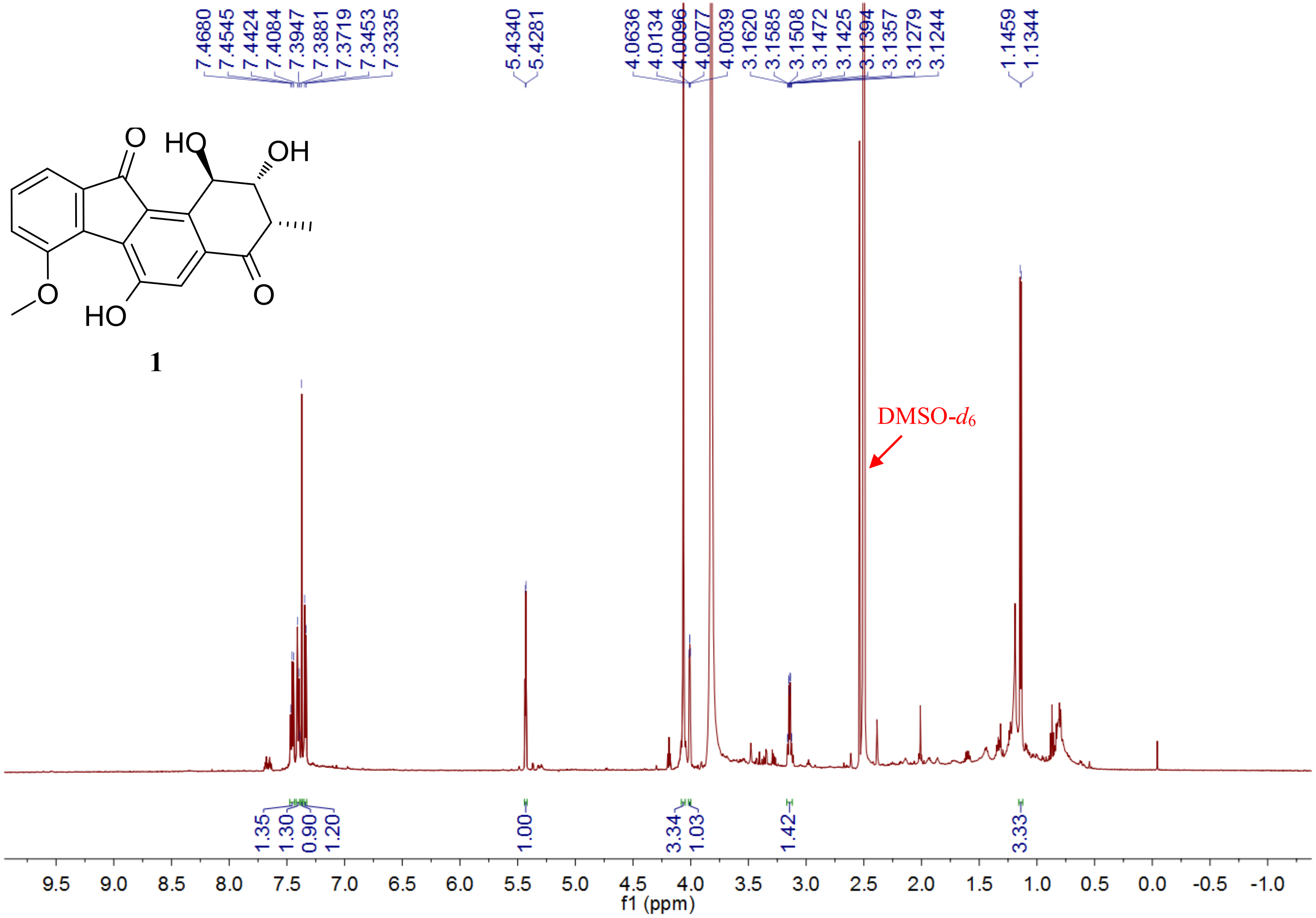

Figure S6. The APT (150 MHz) spectrum of compound **1** in DMSO-d_6

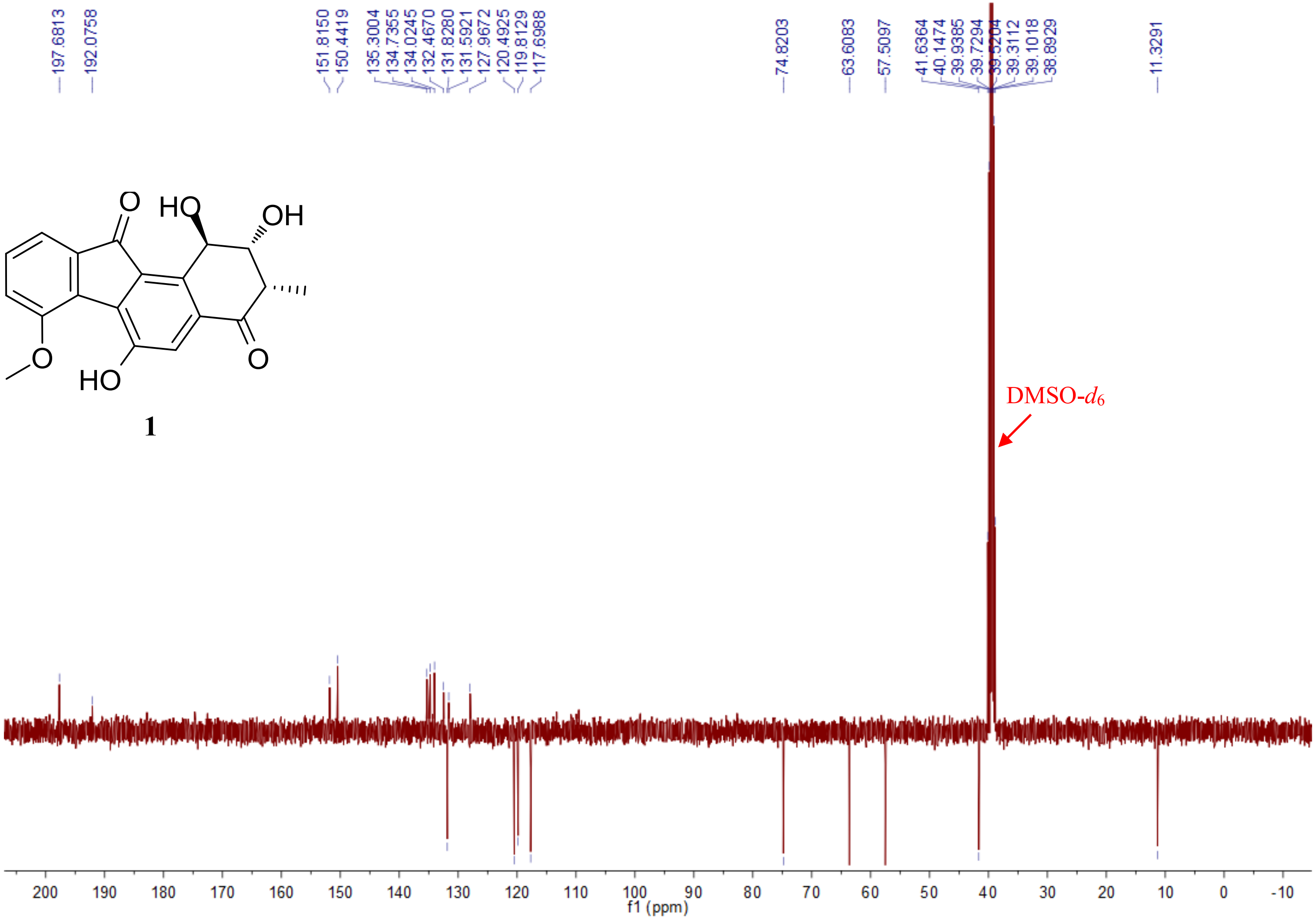

Figure S7. The COSY spectrum of compound **1** in DMSO-d_6

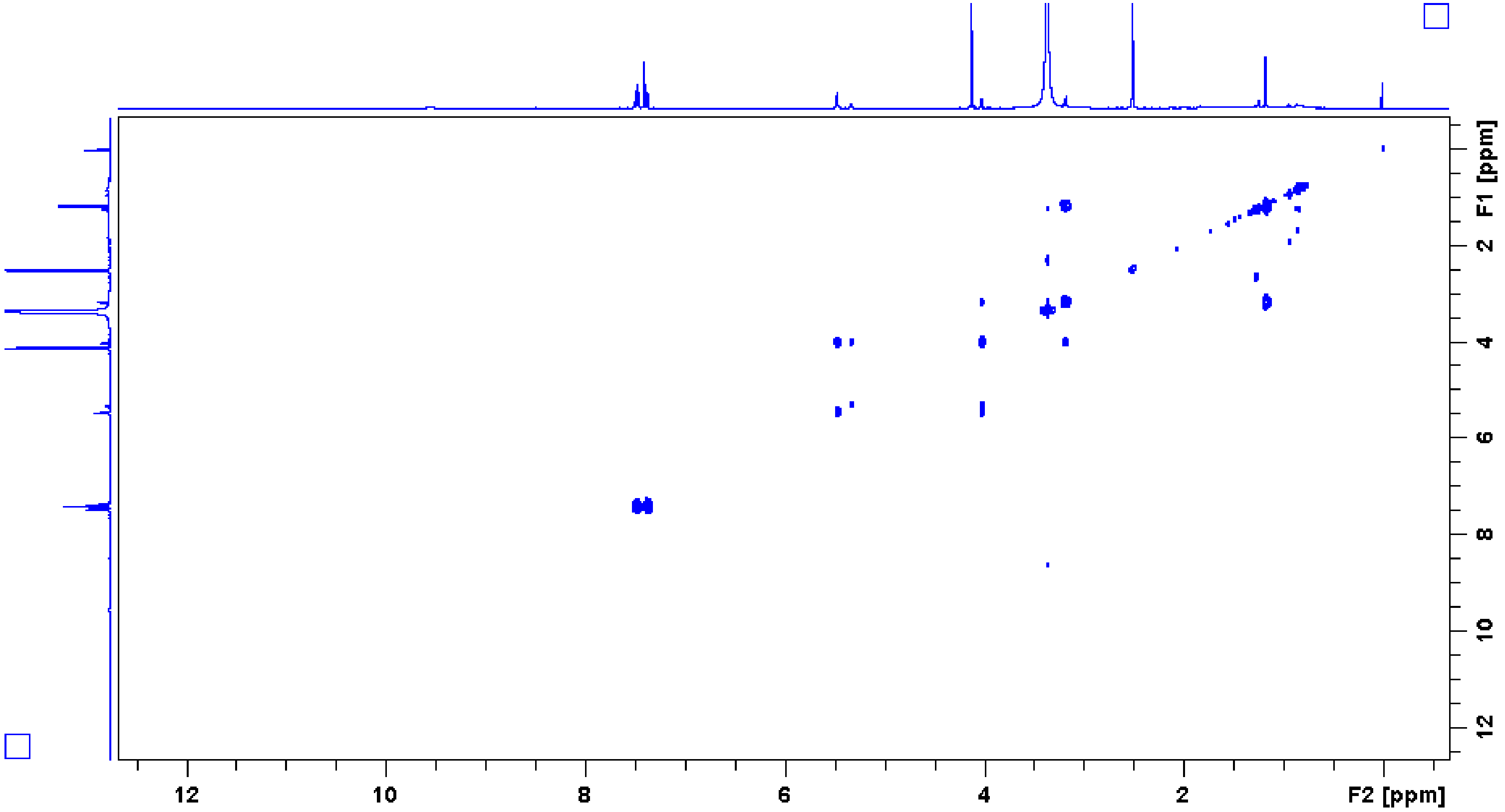

Figure S8. The HSQC spectrum of compound **1** in DMSO-d_6

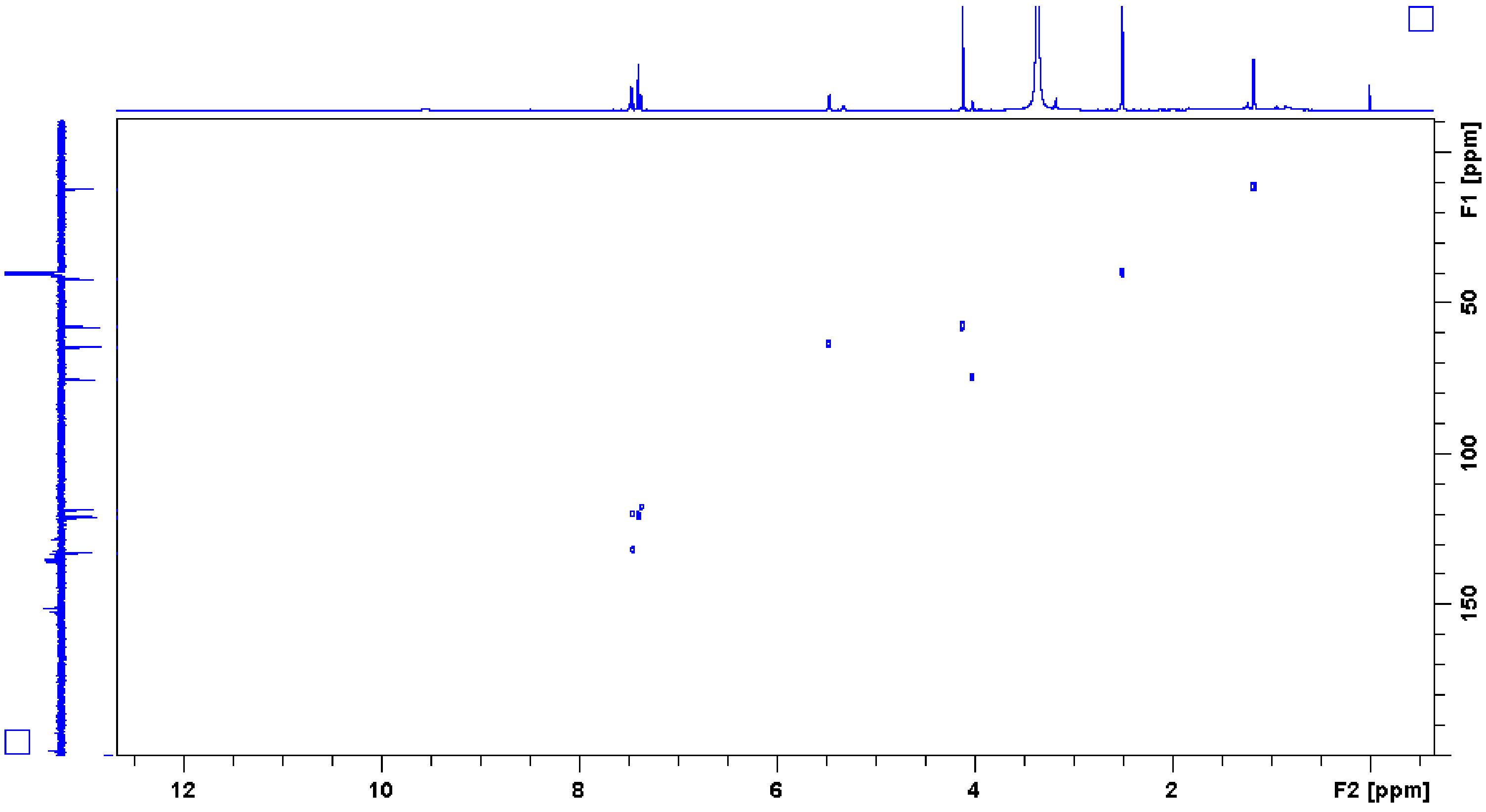

Figure S9. The HMBC spectrum of compound **1** in DMSO-d_6

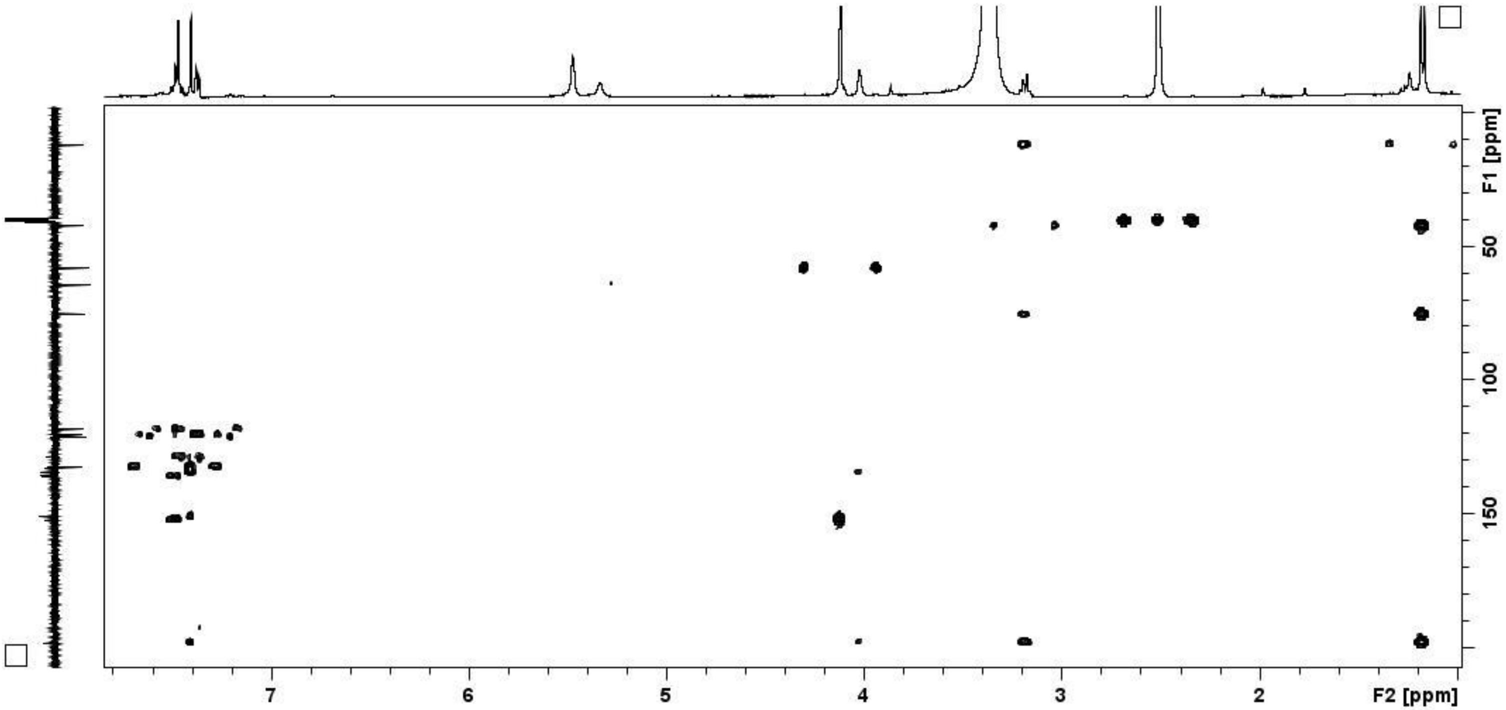

Figure S10. The ROESY spectrum of compound **1** in DMSO-d_6

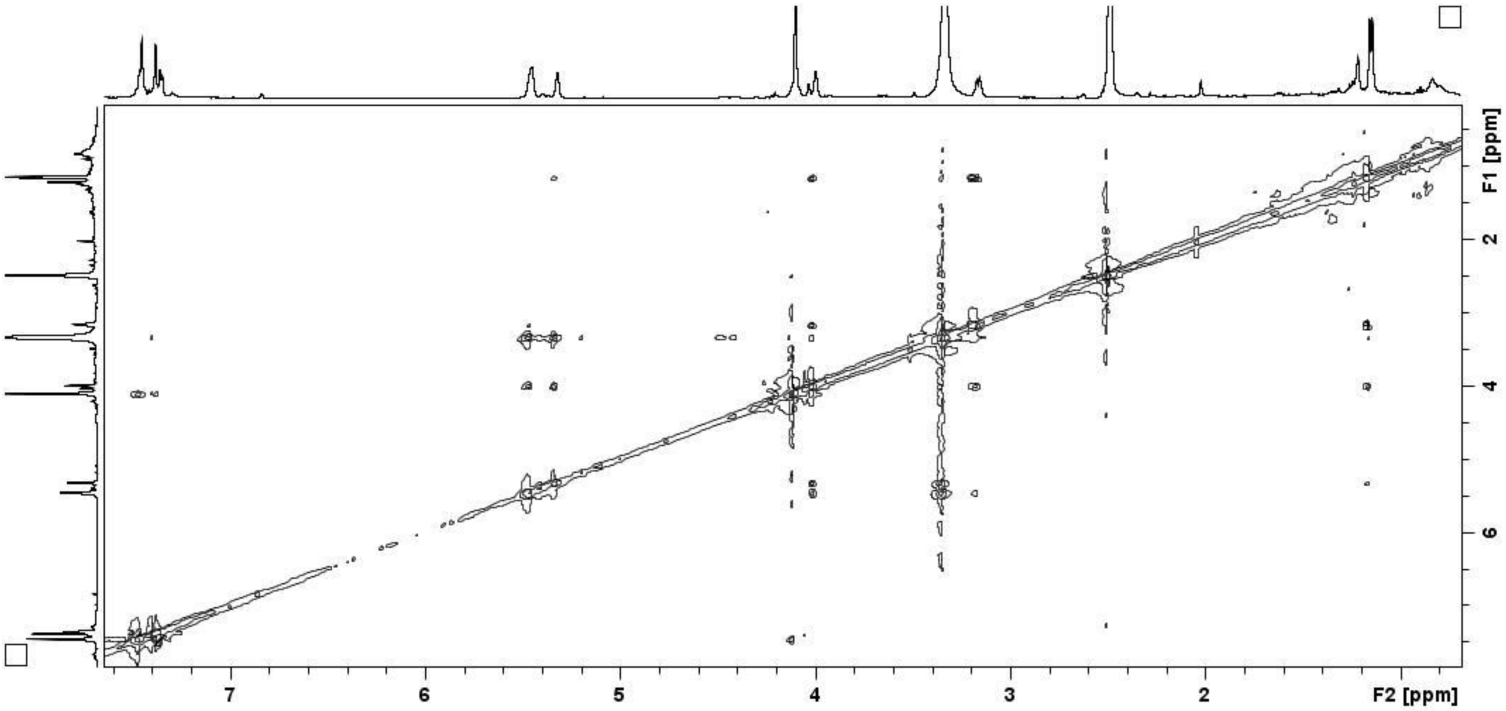

Figure S11. The ROESY spectrum of compound **1** in DMSO-d_6 (D_2O added)

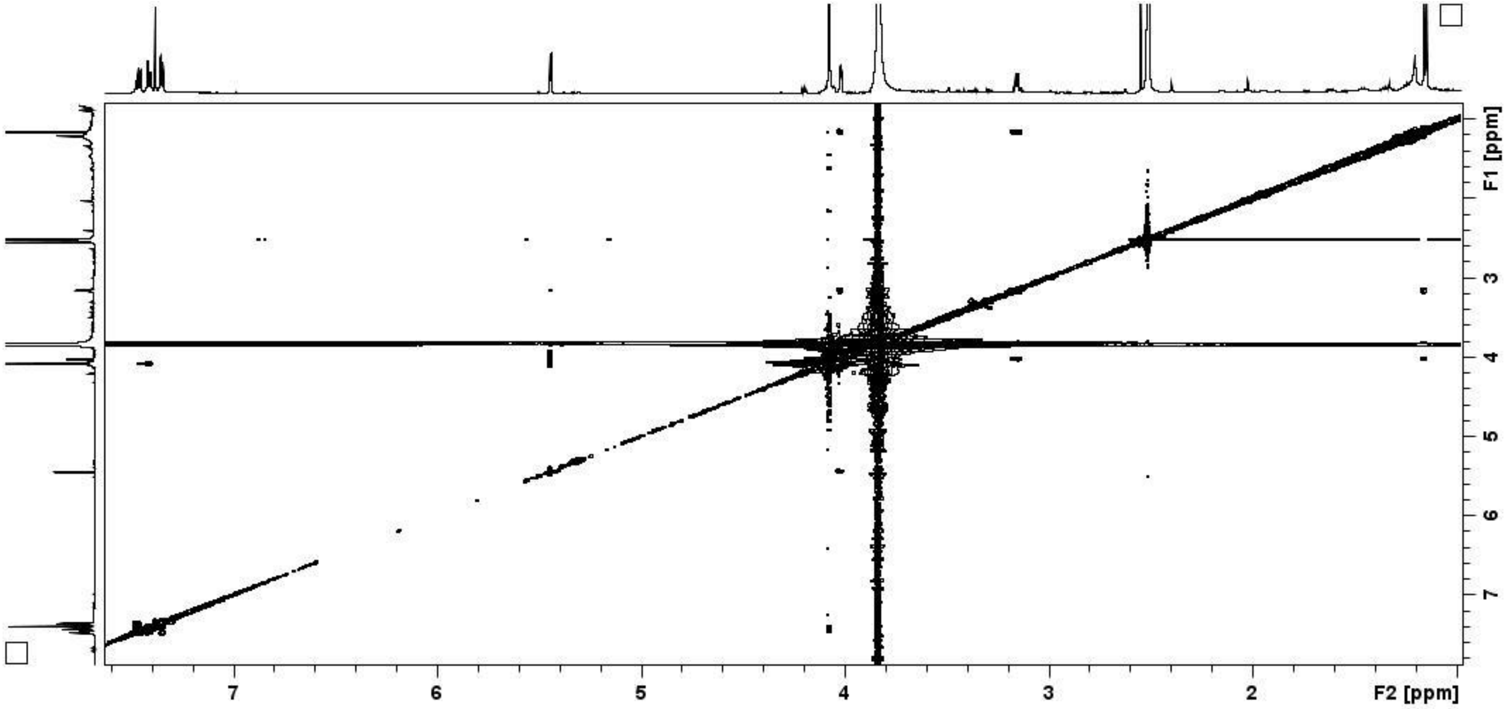

Figure S12. The HRESIMS spectrum of compound **1**

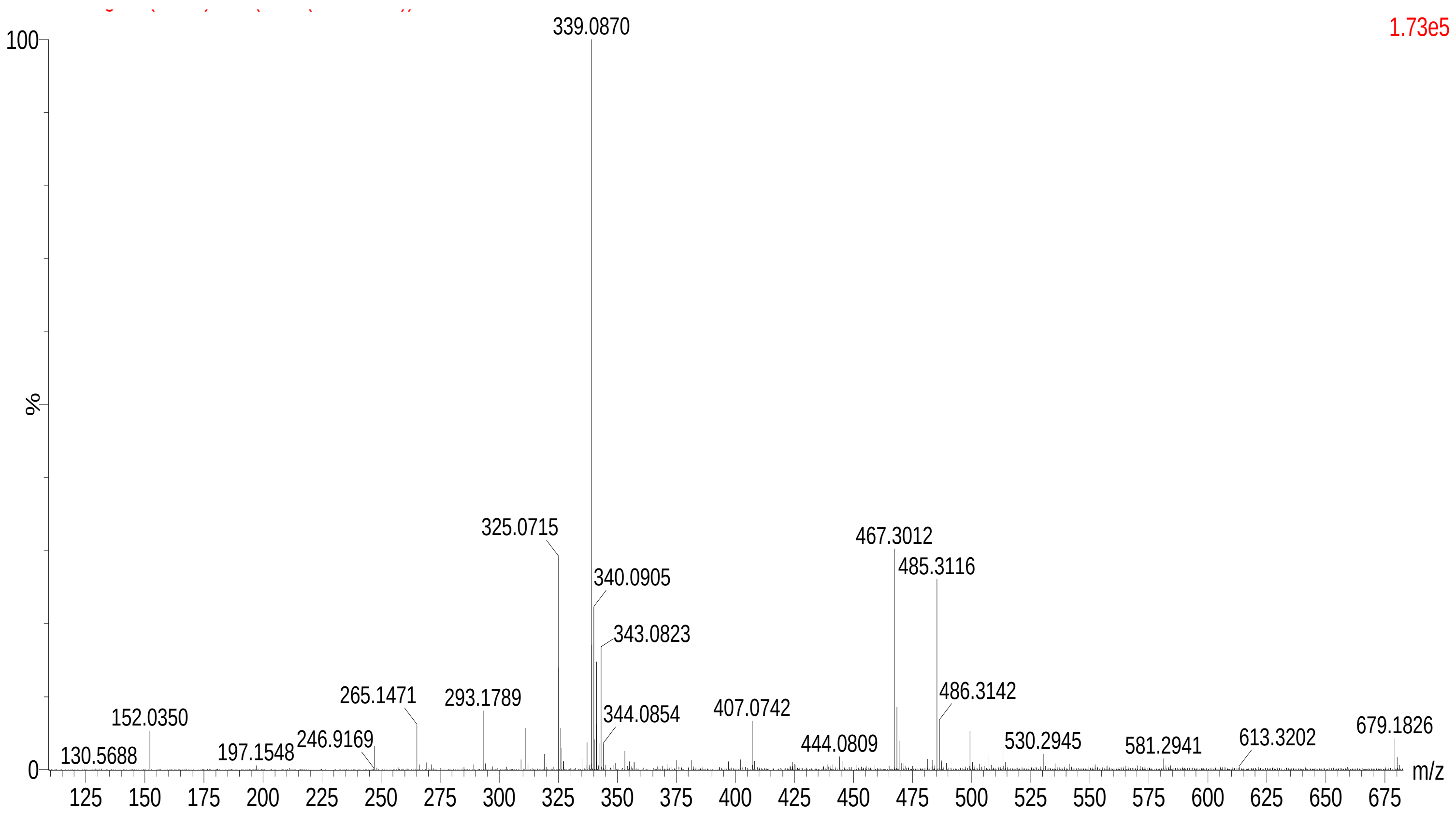

Figure S13. The IR spectrum of compound **1**

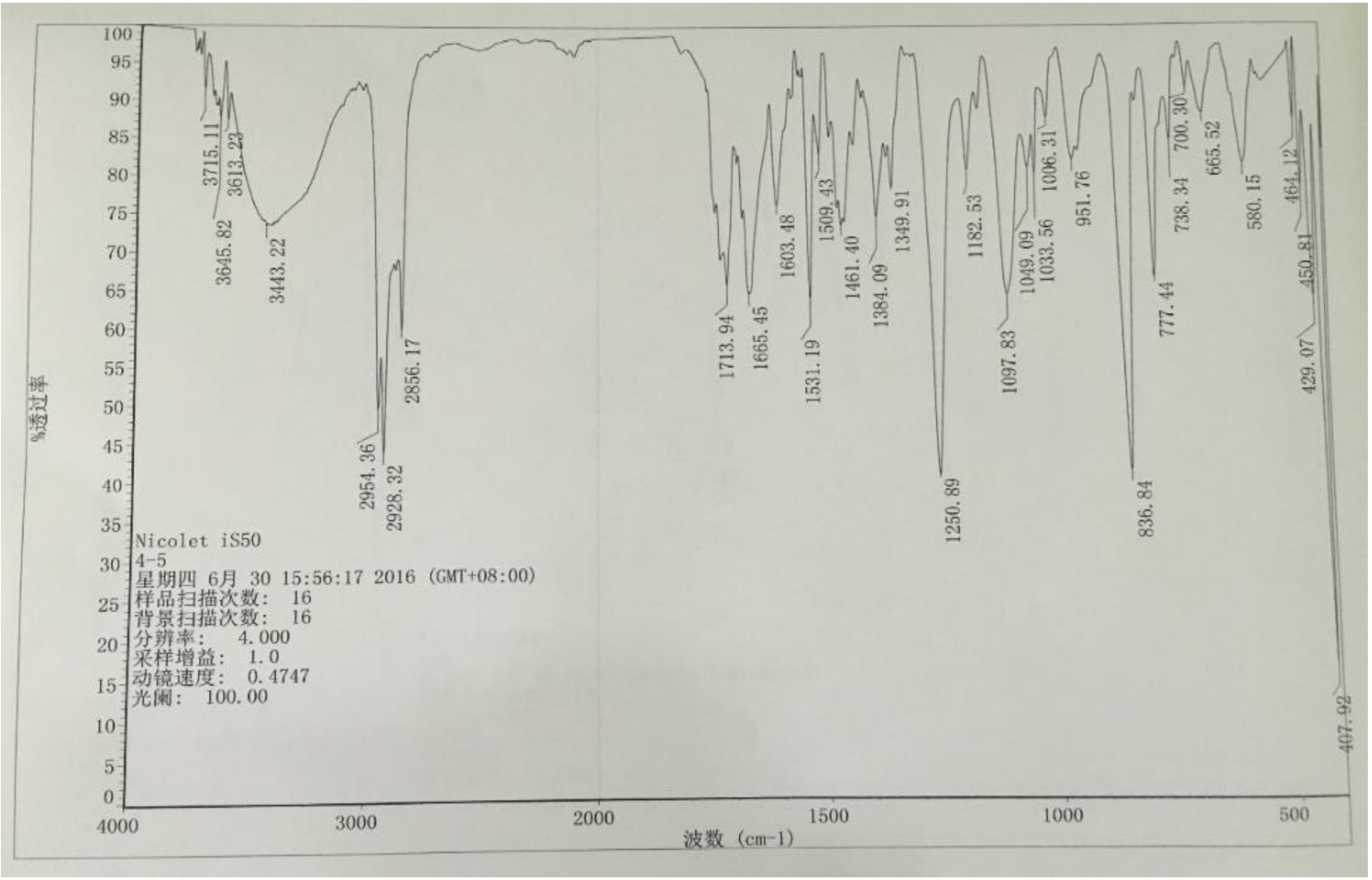

Figure S14. The ^{1}H NMR (600 MHz) spectrum of compound **2** in DMSO-d_6

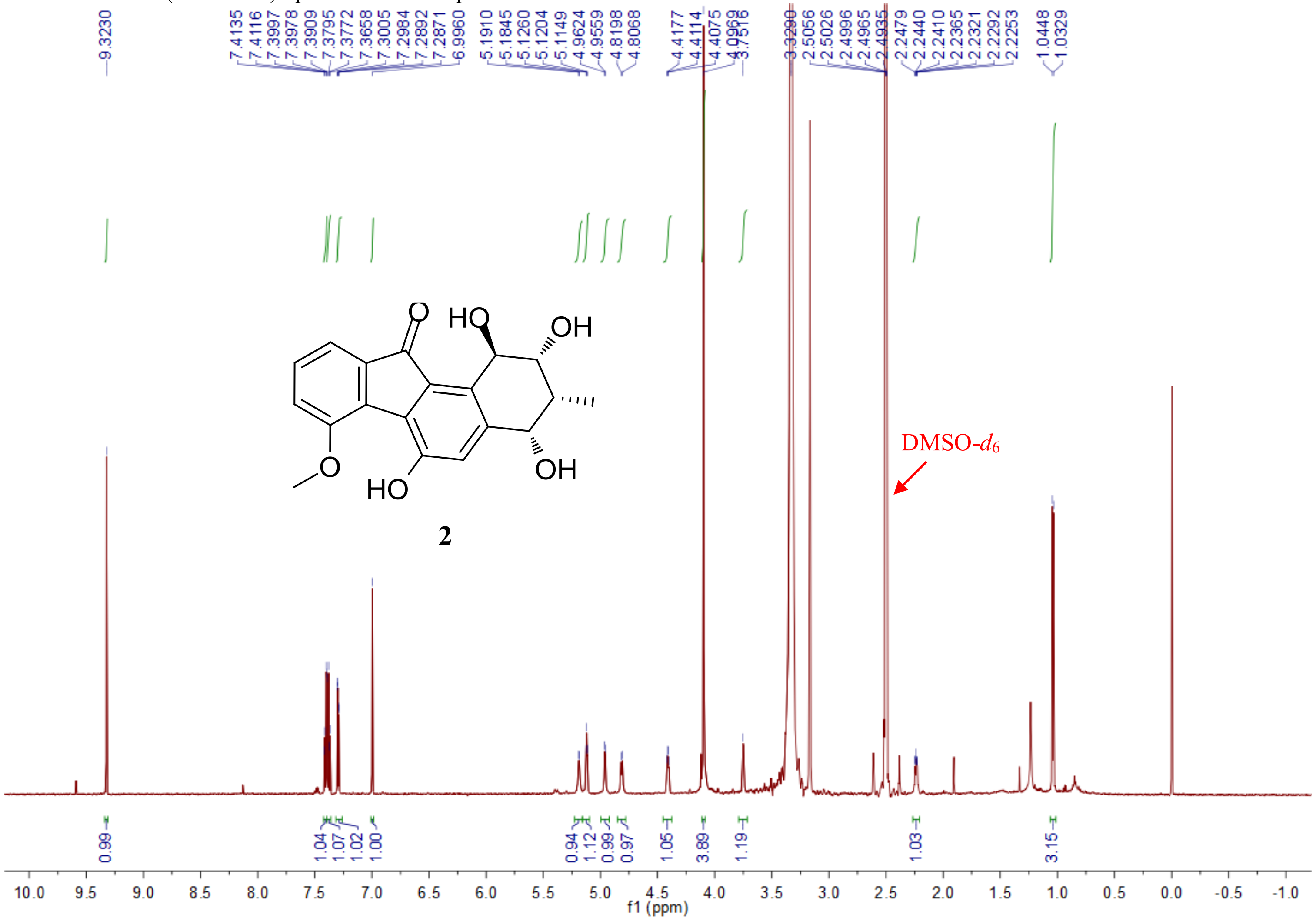

Figure S15. The ^{13}C NMR (150 MHz) spectrum of compound **2** in DMSO-d_6

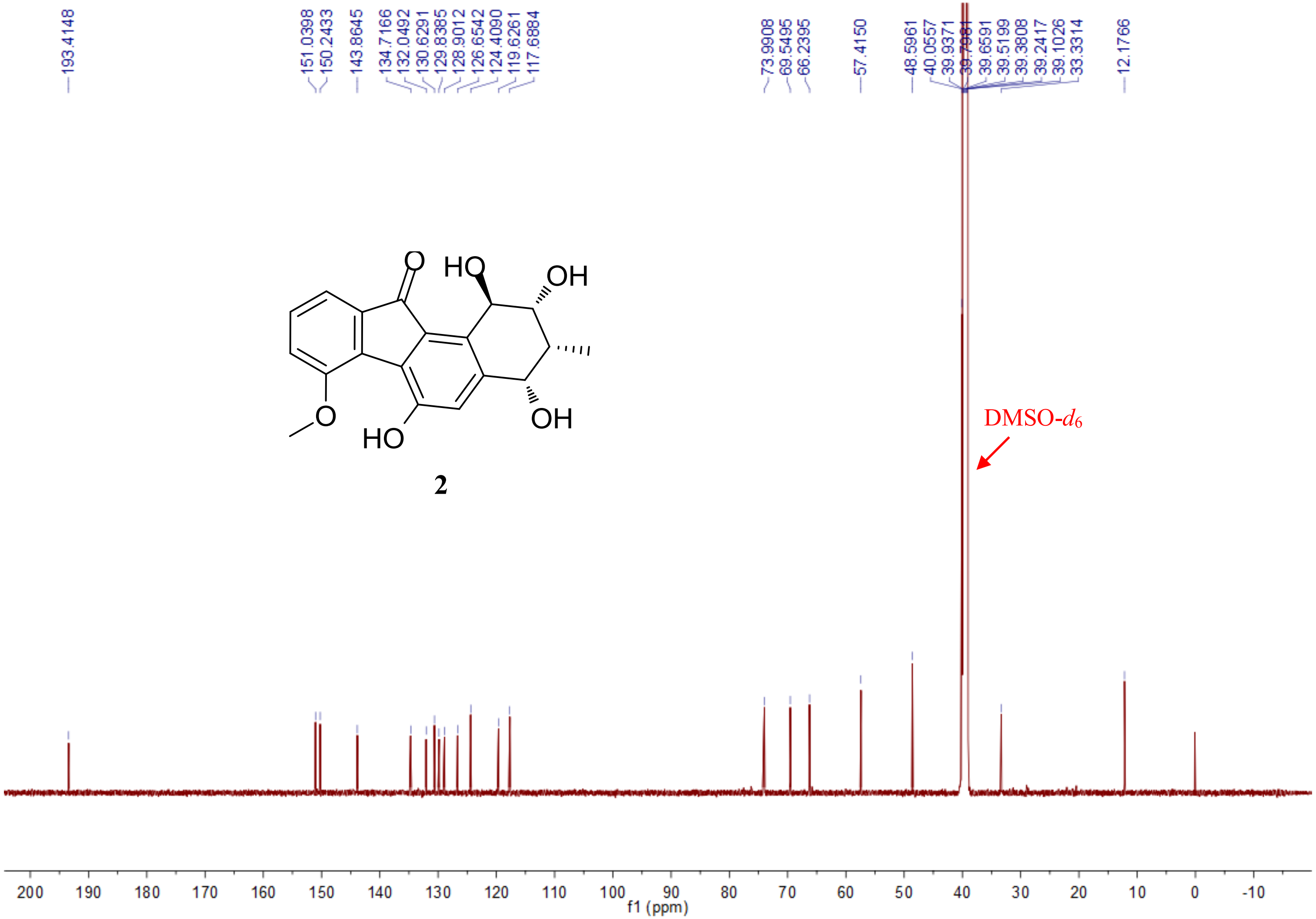

Figure S16. The COSY spectrum of compound **2** in DMSO-d_6

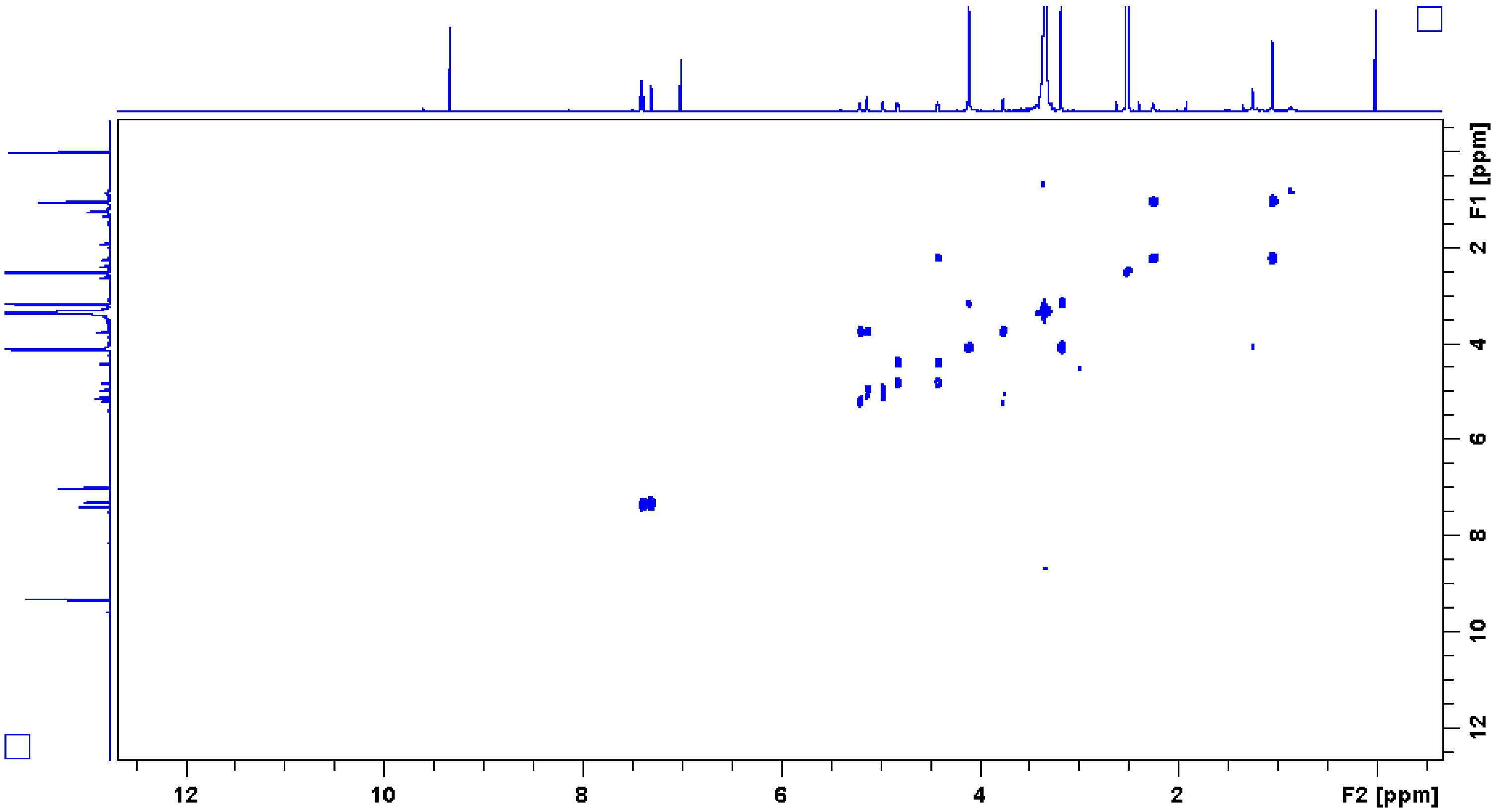

Figure S17. The HSQC spectrum of compound **2** in DMSO-d_6

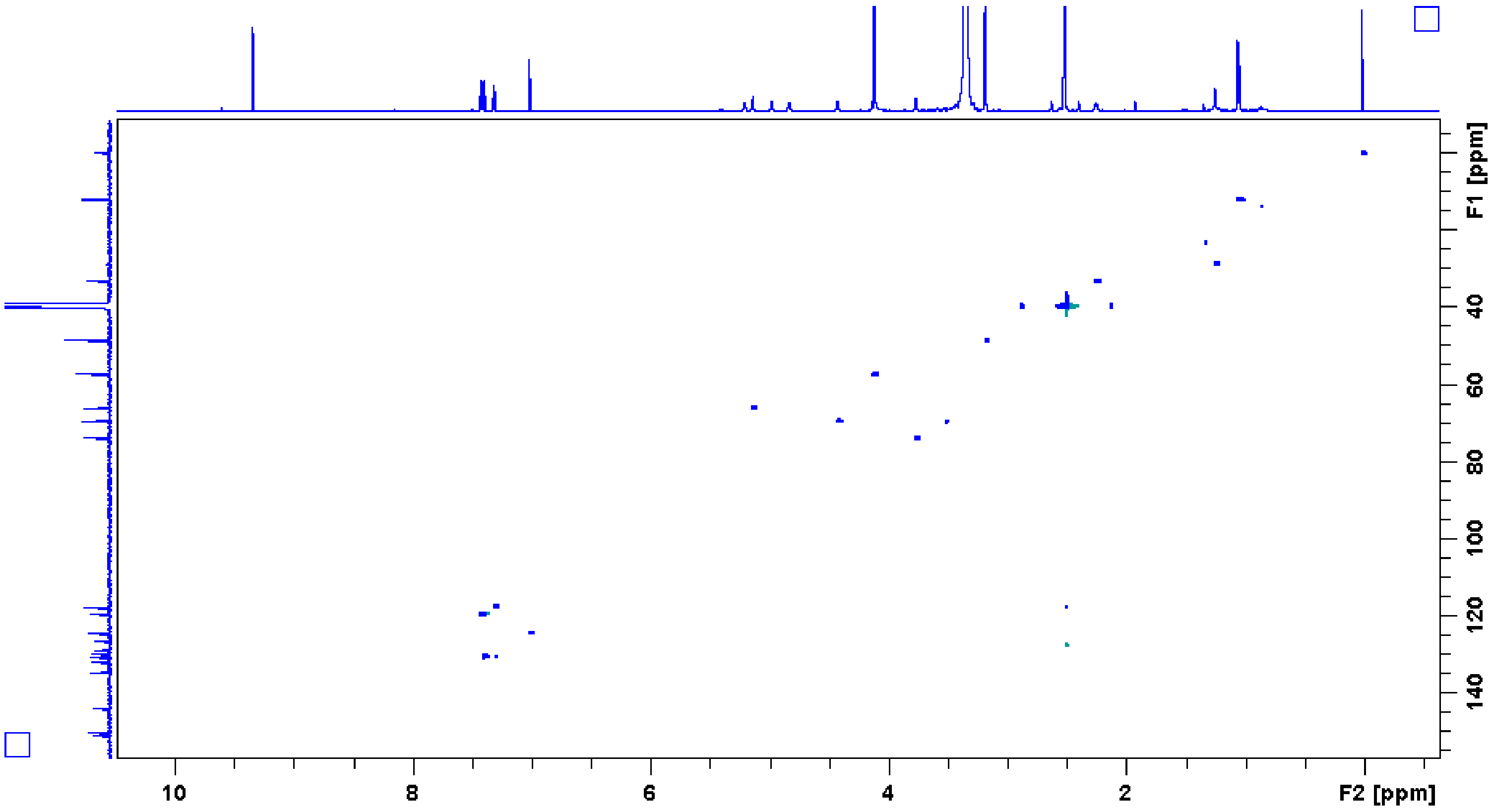

Figure S18. The HMBC spectrum of compound **2** in DMSO-d_6

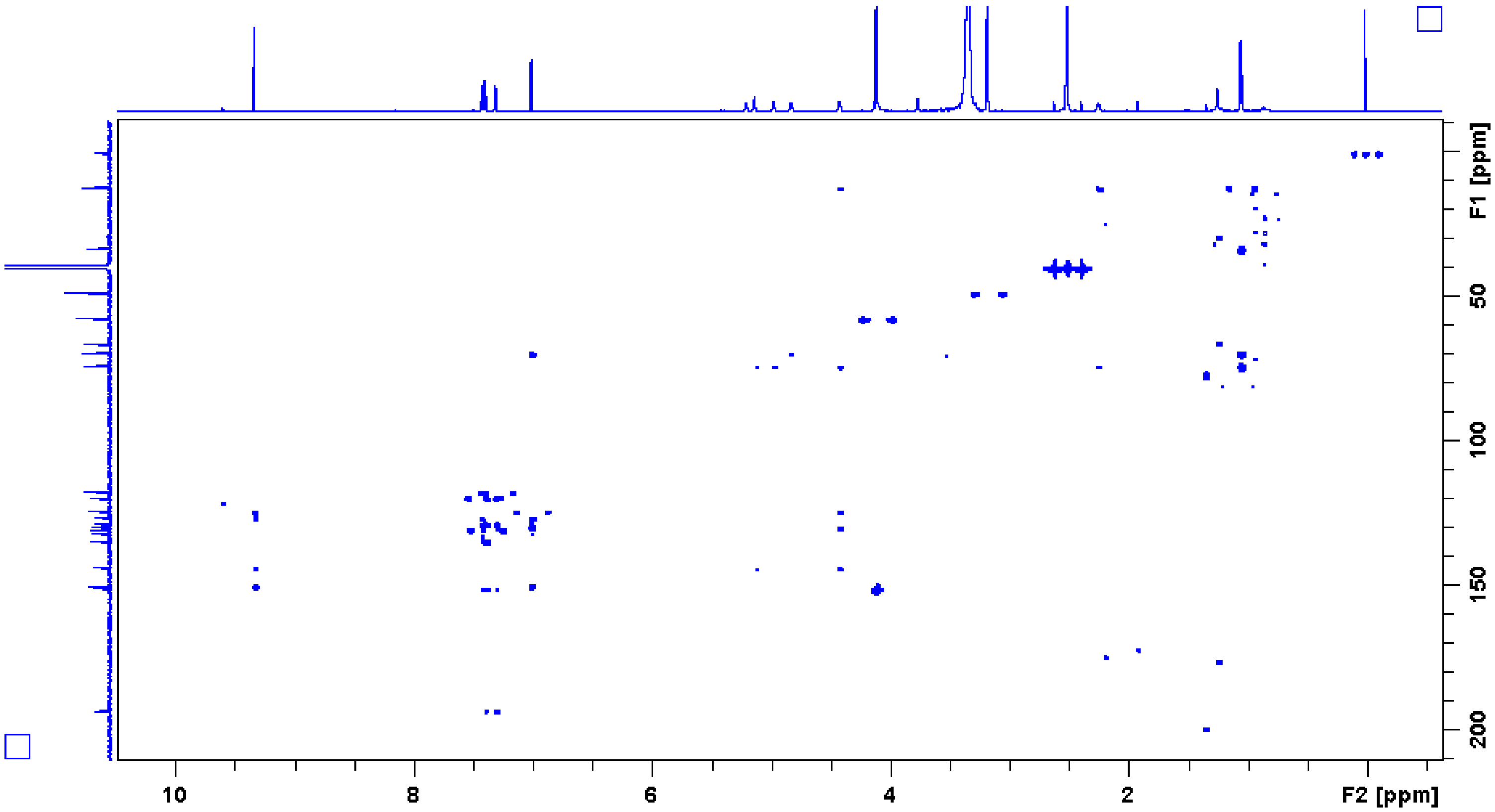

Figure S19. The ROESY spectrum of compound **2** in DMSO-d_6

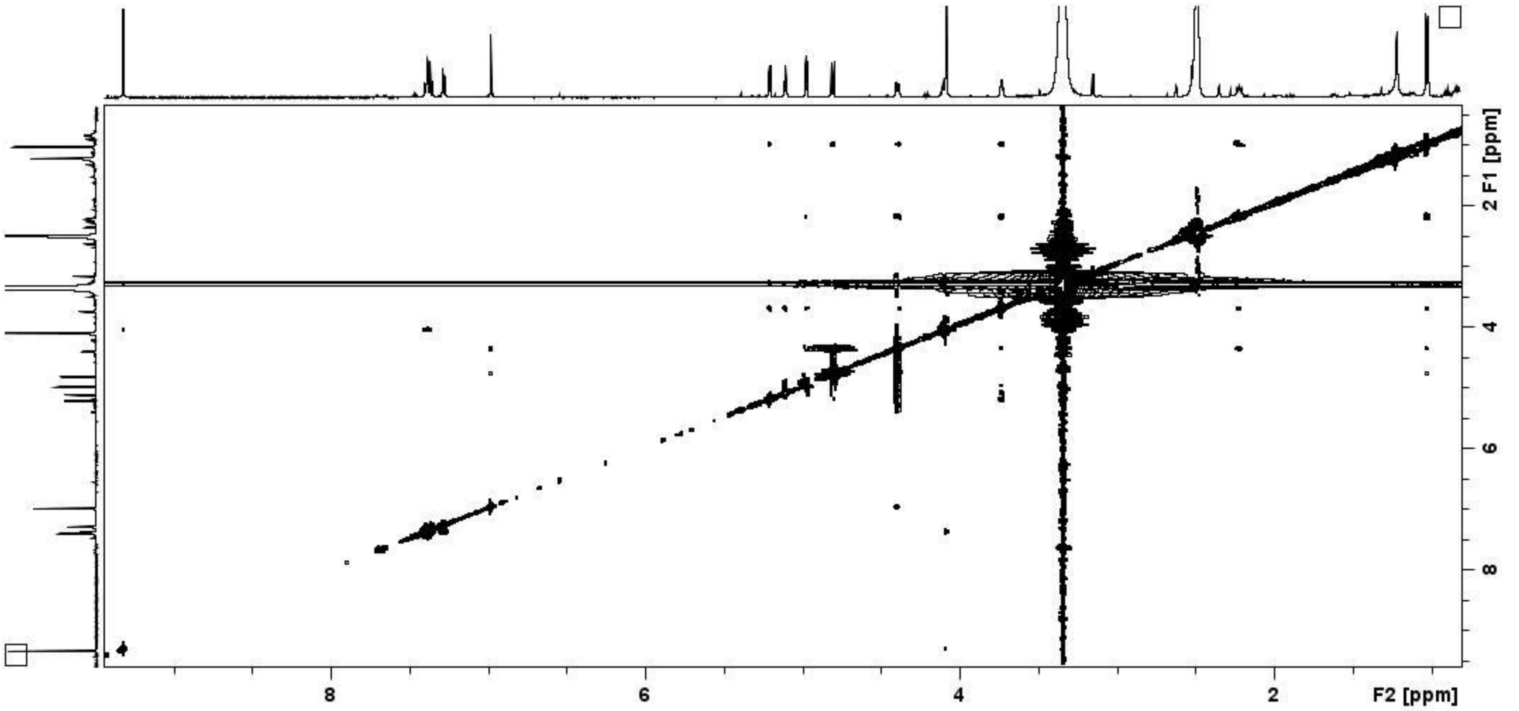

Figure S20. The HRESIMS spectrum of compound **2**

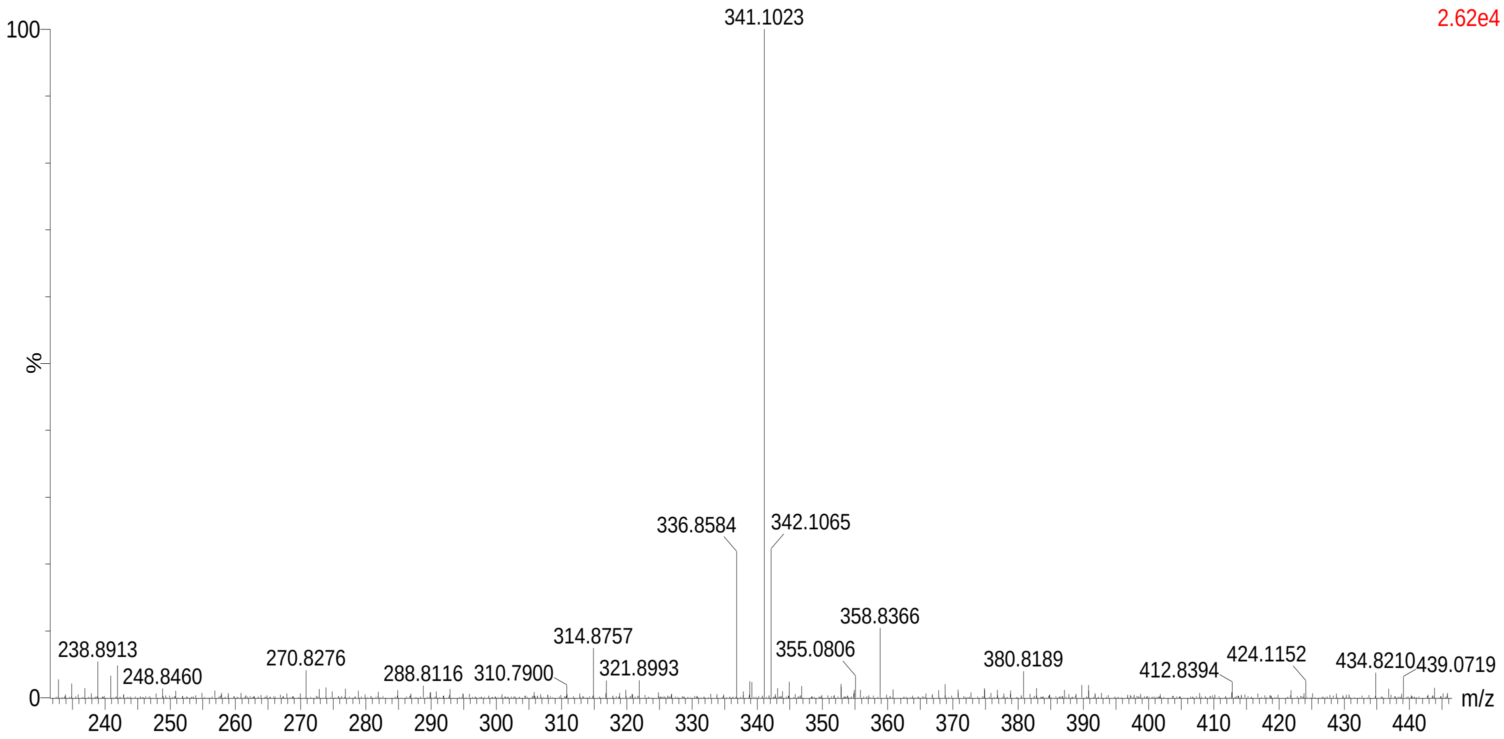

Figure S21. The IR spectrum of compound **2**

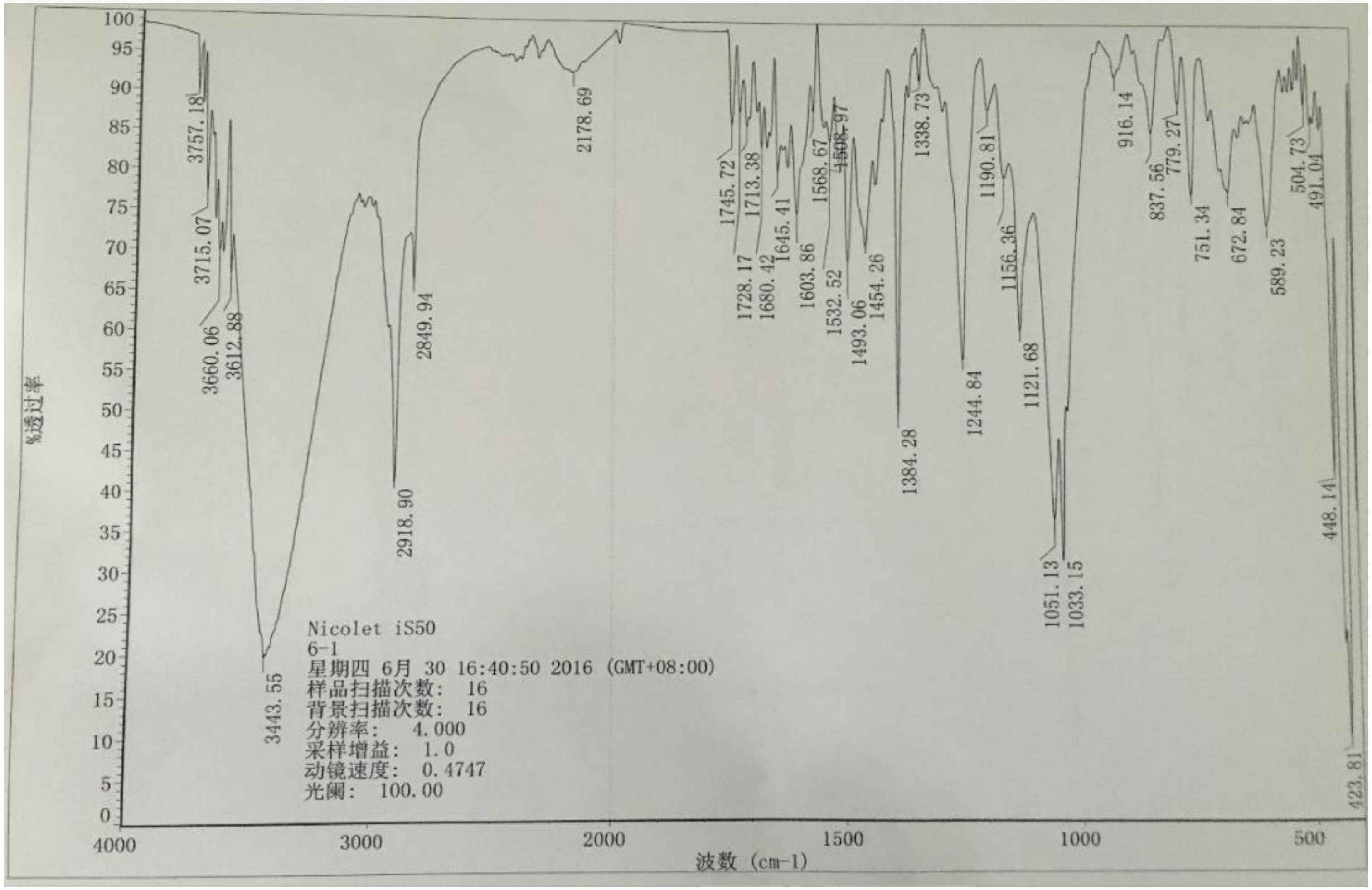

Figure S22. The ^{1}H NMR (600 MHz) spectrum of compound **3** in DMSO-d_6

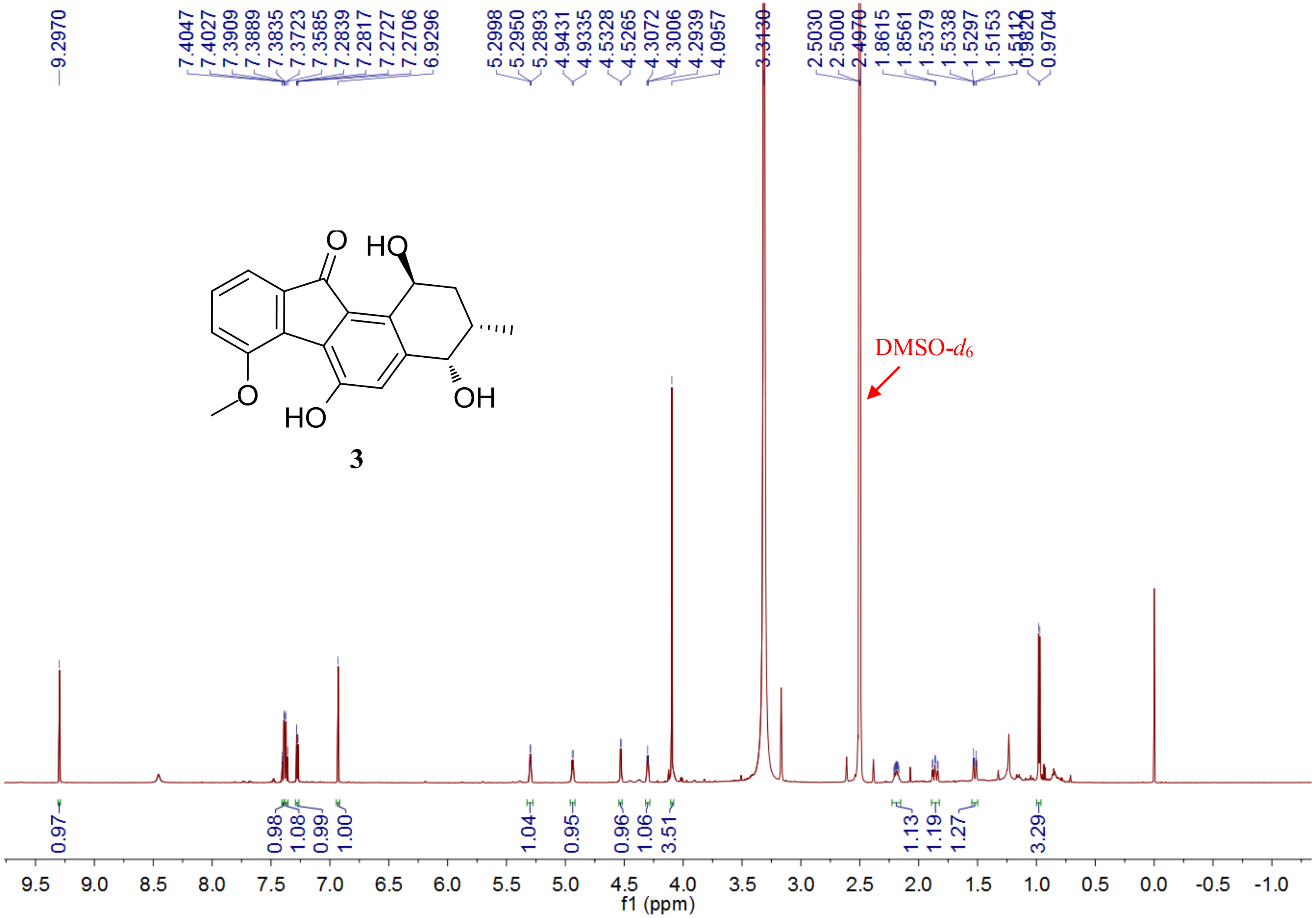

Figure S23. The ^{13}C NMR (150 MHz) spectrum of compound **3** in DMSO-d_6

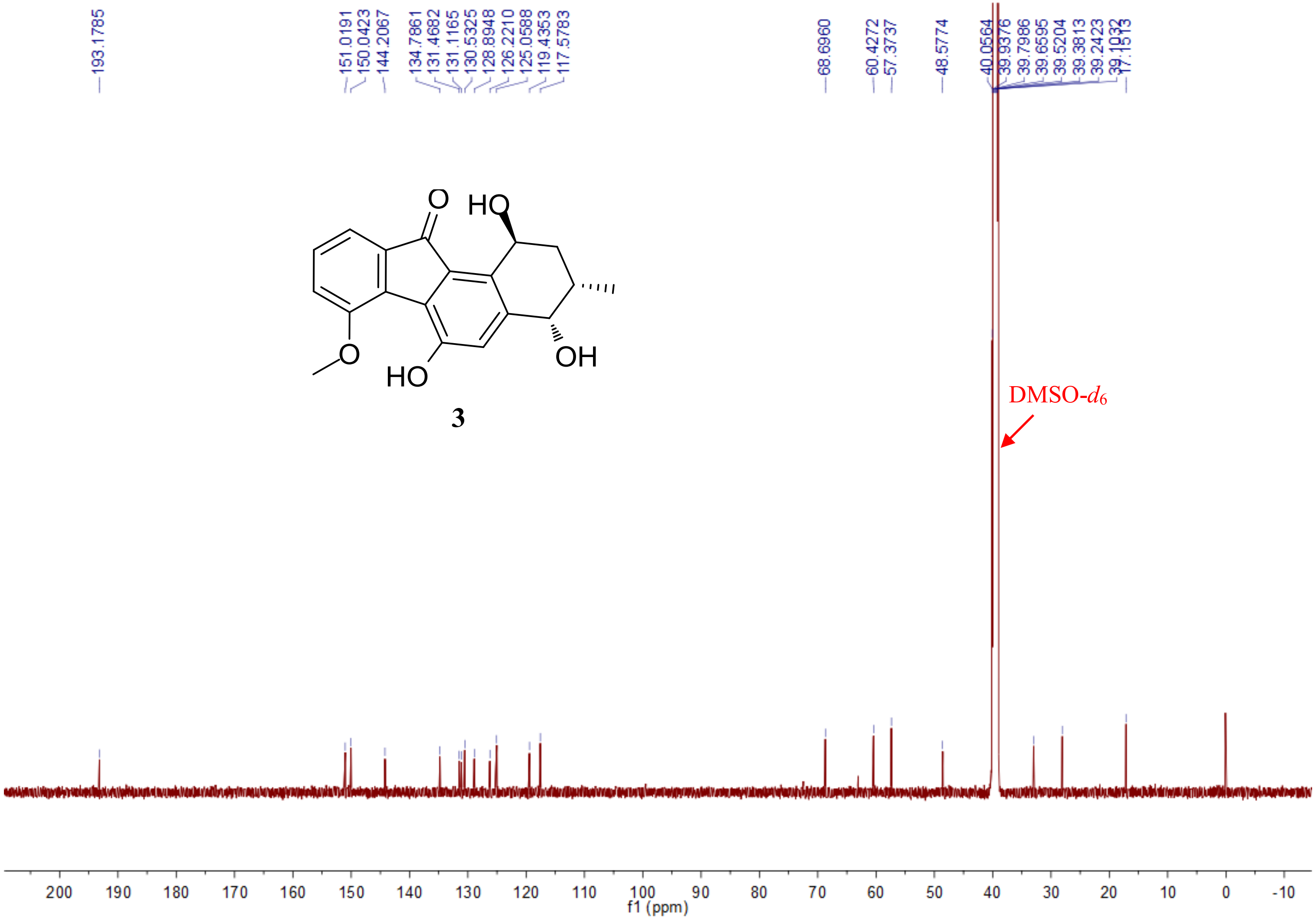

Figure S24. The COSY spectrum of compound **3** in DMSO-d_6

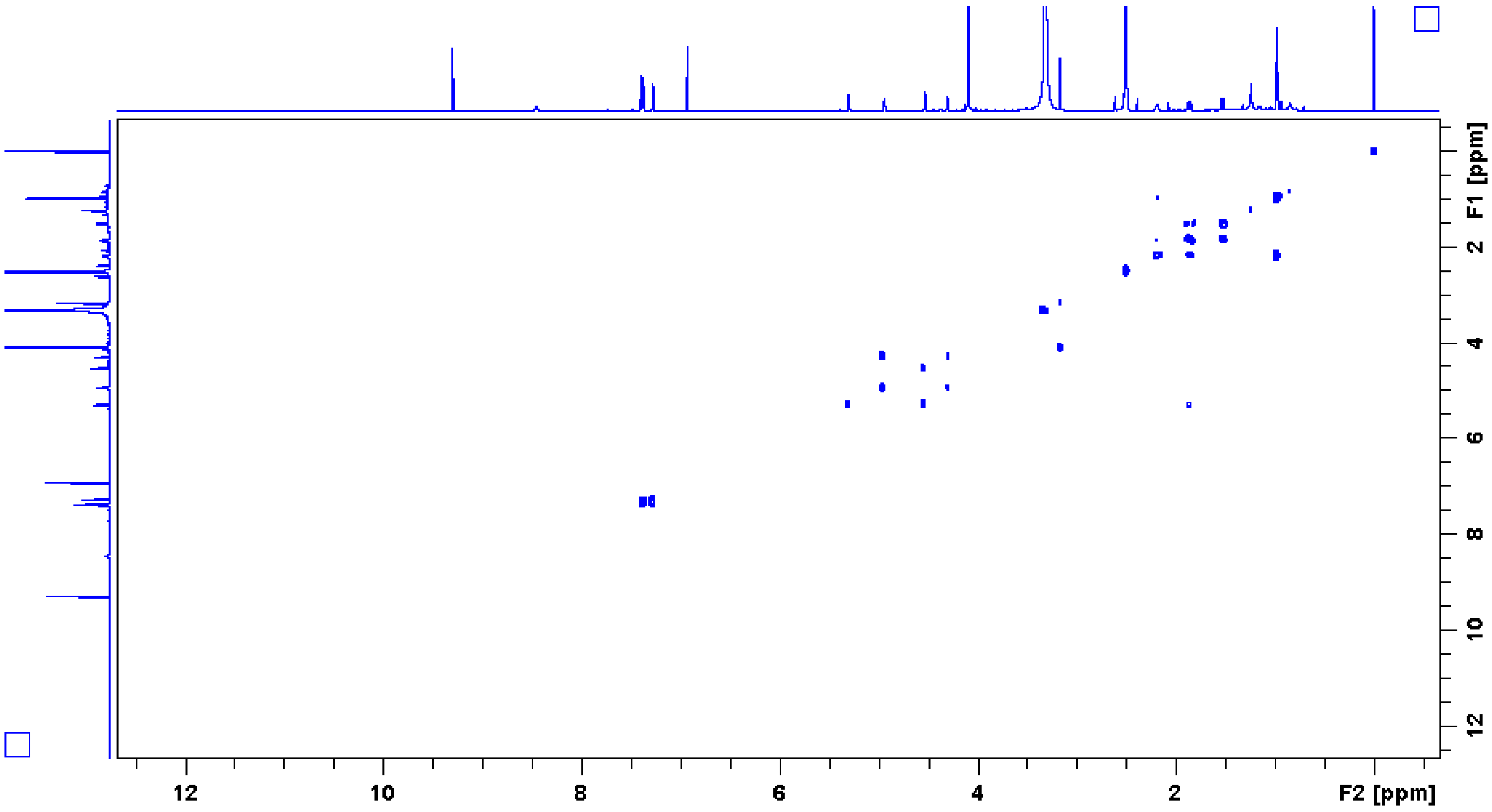

Figure S25. The HSQC spectrum of compound **3** in DMSO-d_6

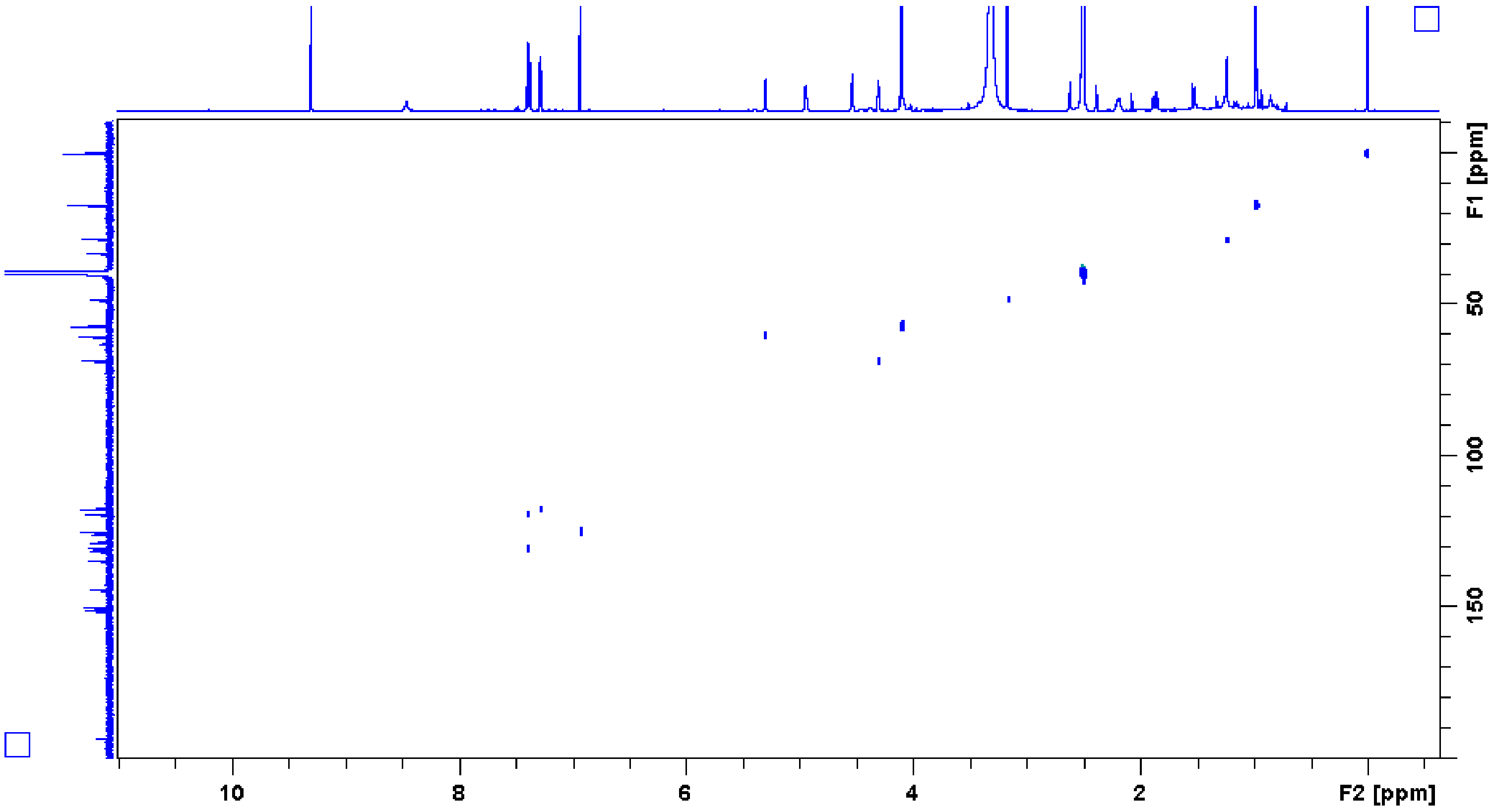

Figure S26. The HMBC spectrum of compound **3** in DMSO-d_6

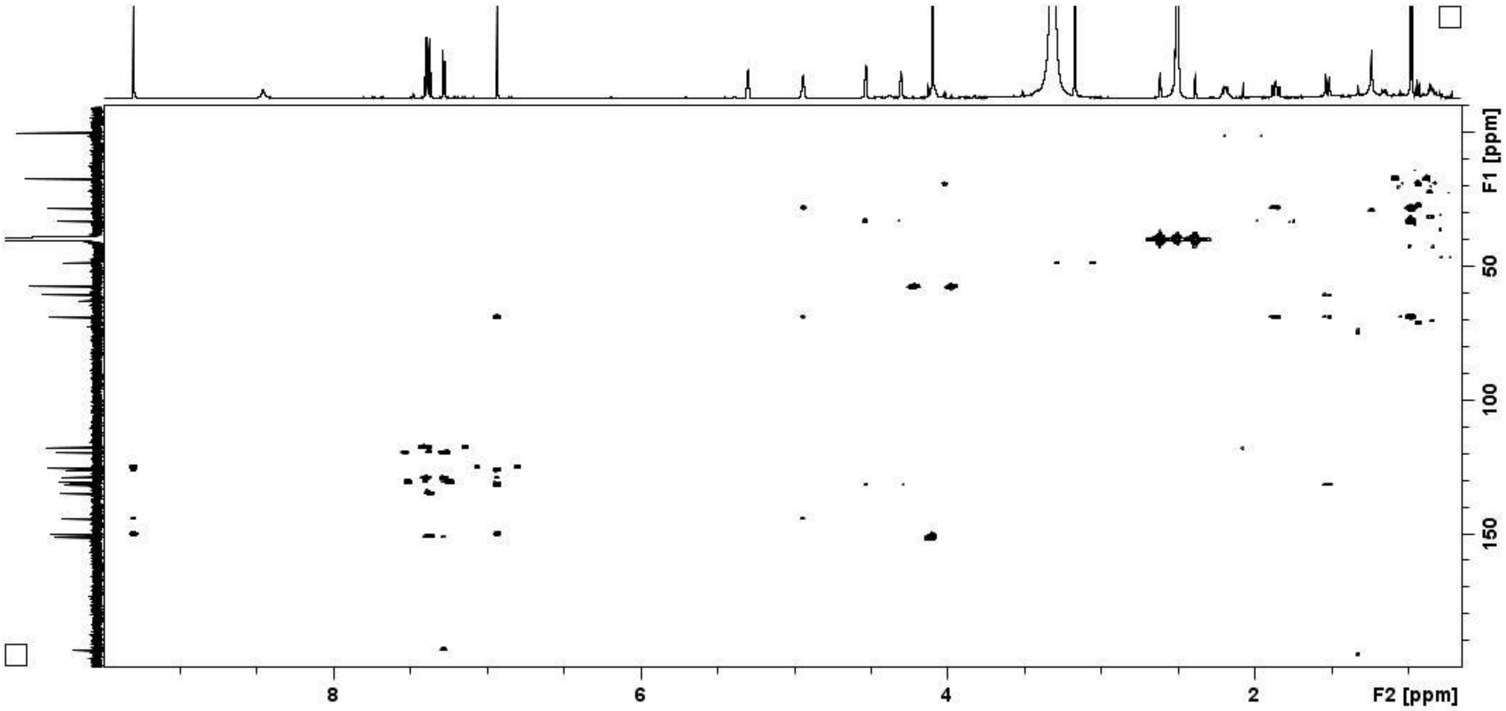

Figure S27. The ROESY spectrum of compound **3** in DMSO-d_6

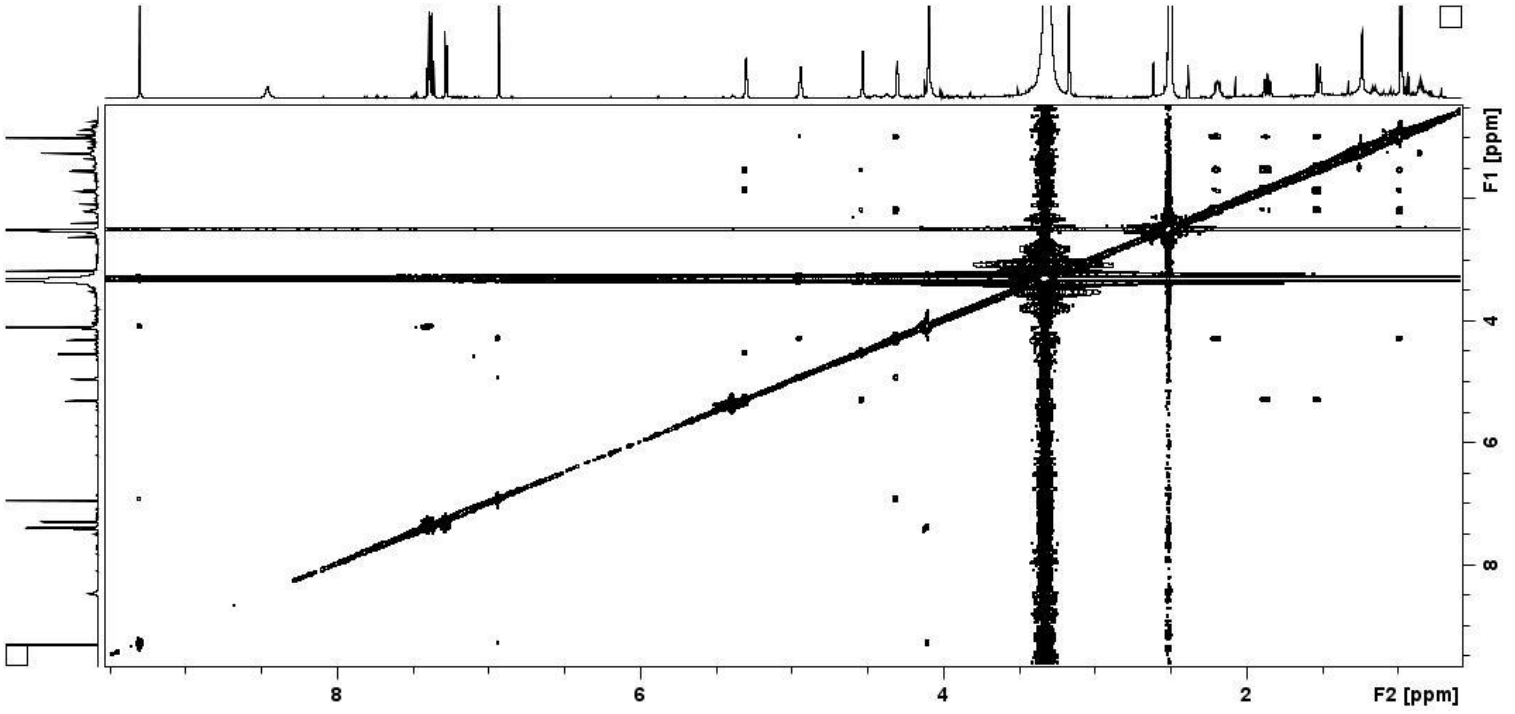

Figure S28. The HRESIMS spectrum of compound **3**

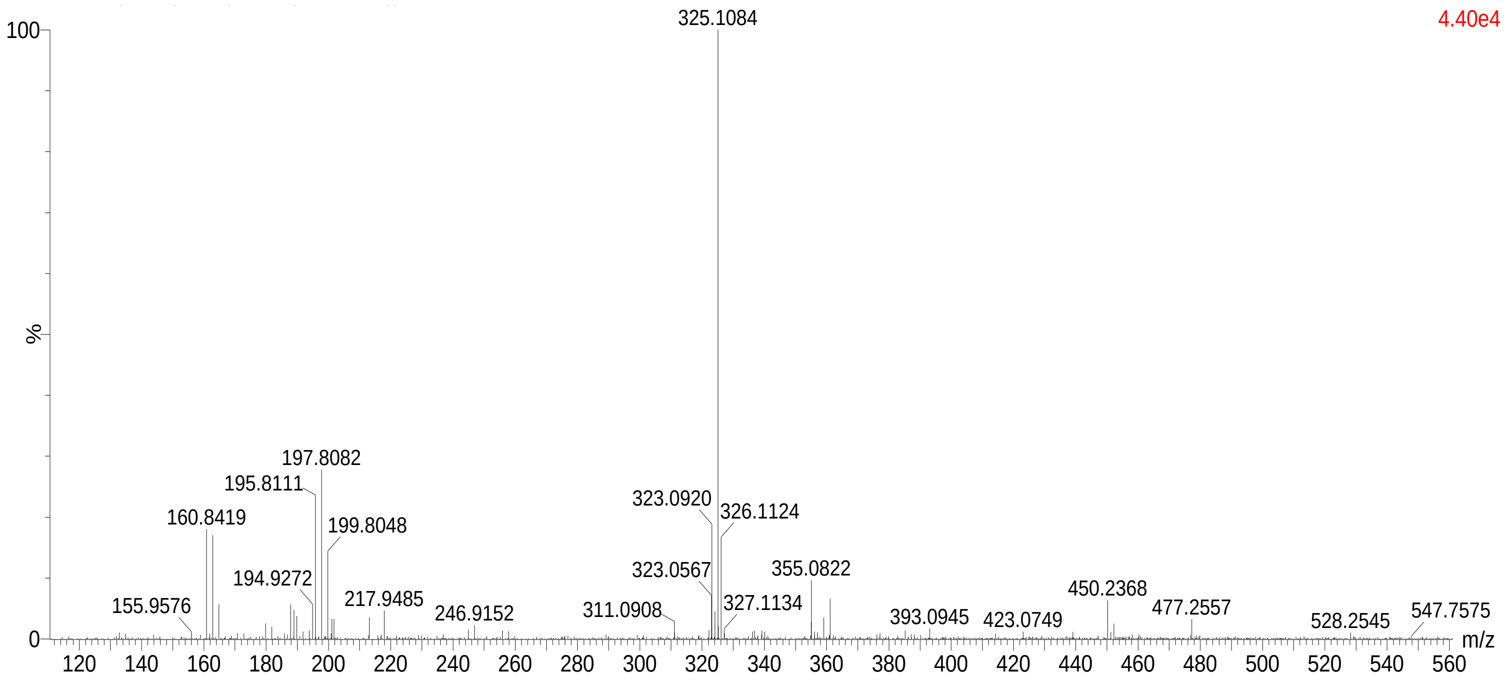

Figure S29. The IR spectrum of compound **3**

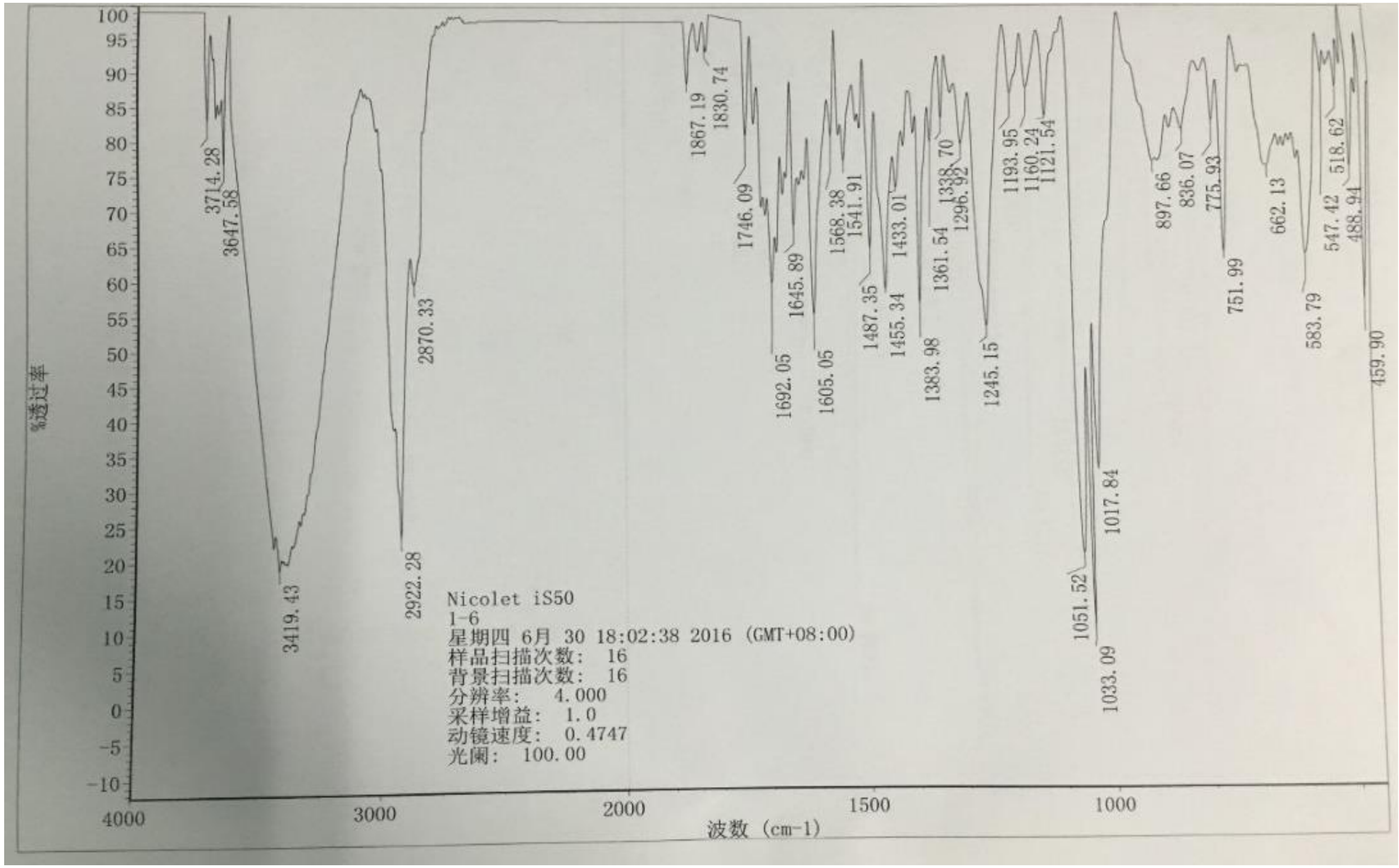

Figure S30. The ^{1}H NMR (600 MHz) spectrum of compound **4** in DMSO-d_6

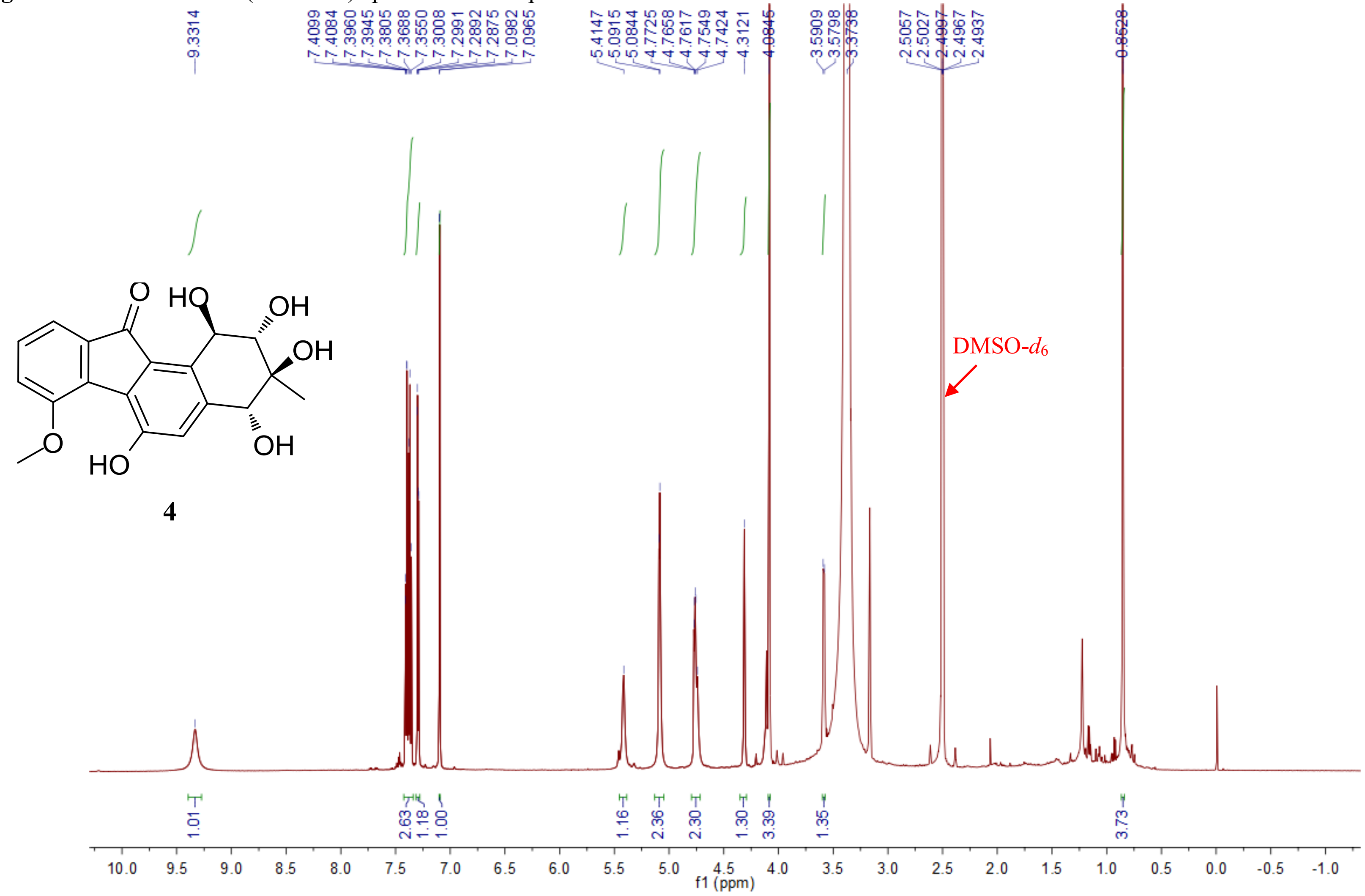

Figure S31. The APT (150 MHz) spectrum of compound **4** in DMSO-d_6

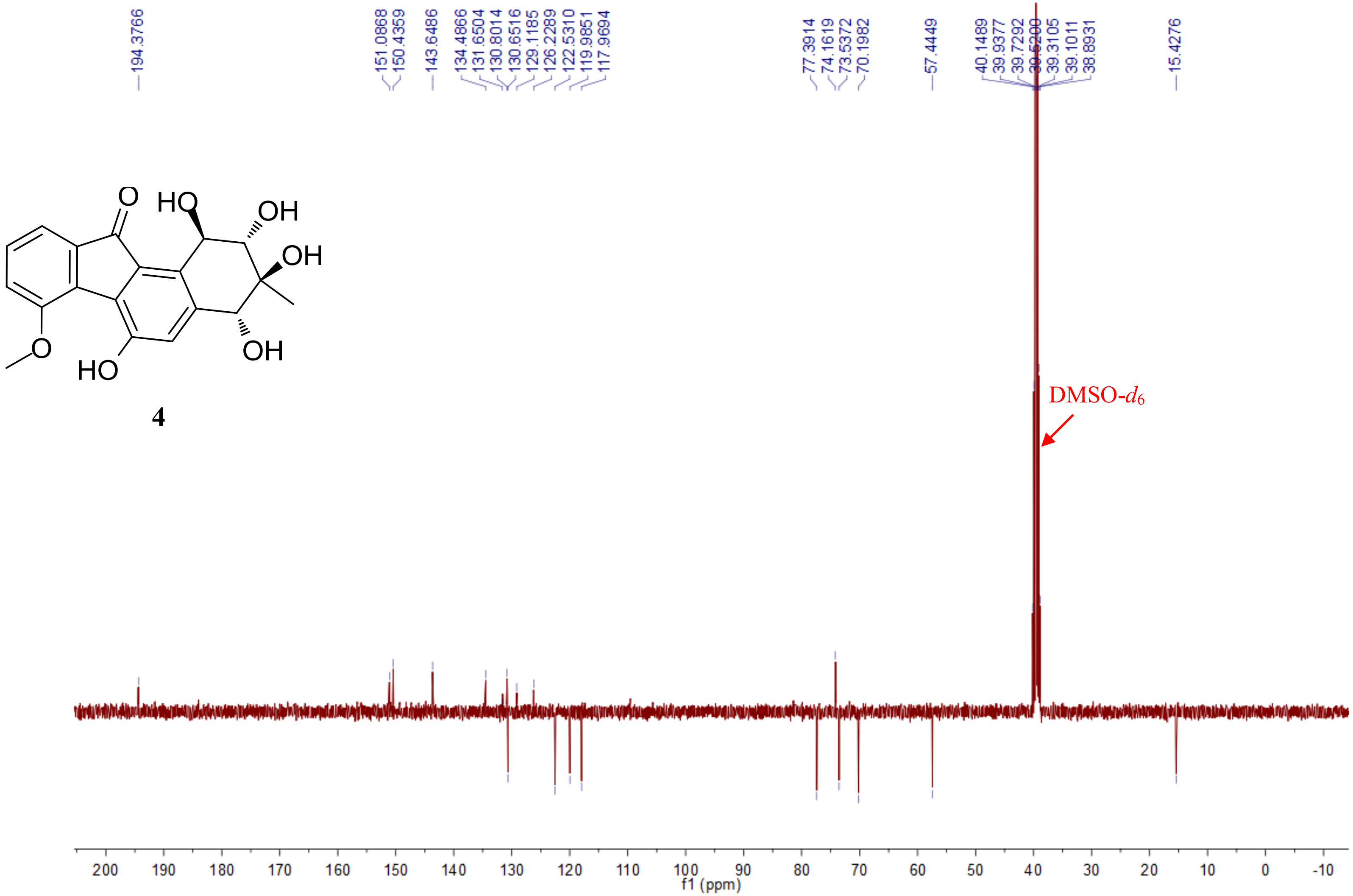

Figure S32. The COSY spectrum of compound **4** in DMSO-d_6

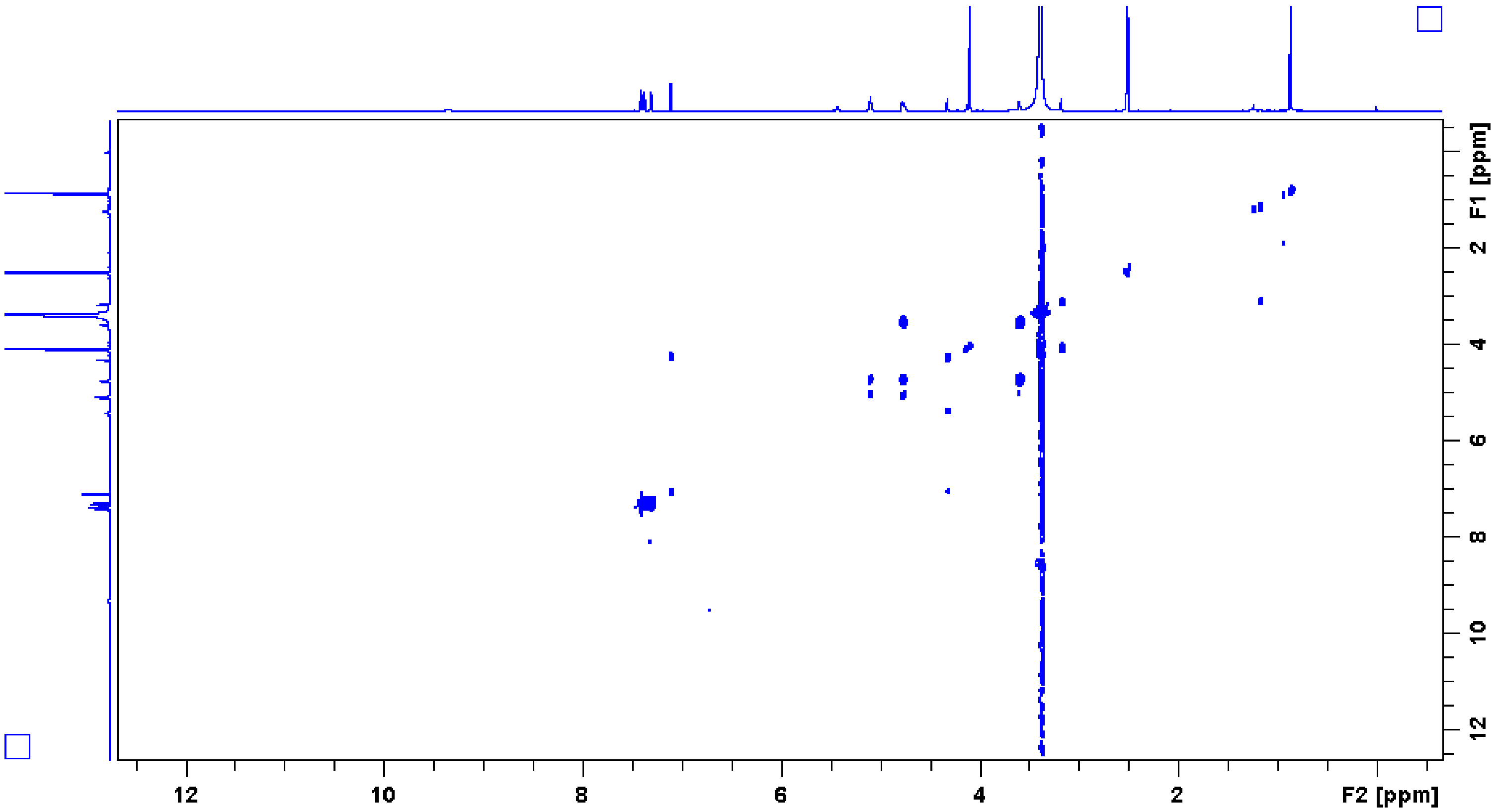

Figure S33. The HSQC spectrum of compound **4** in DMSO-d_6

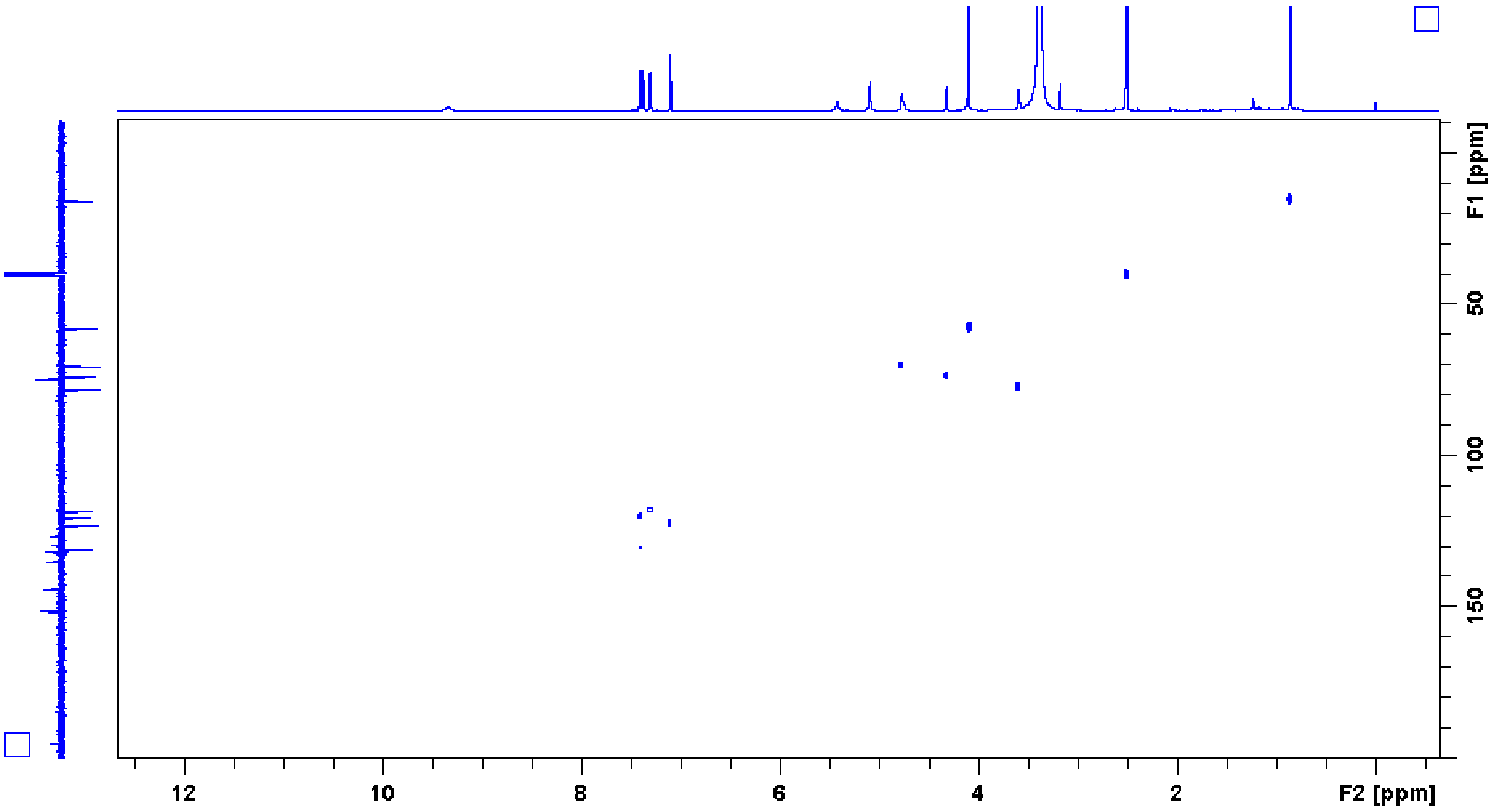

Figure S34. The HMBC spectrum of compound **4** in DMSO-d_6

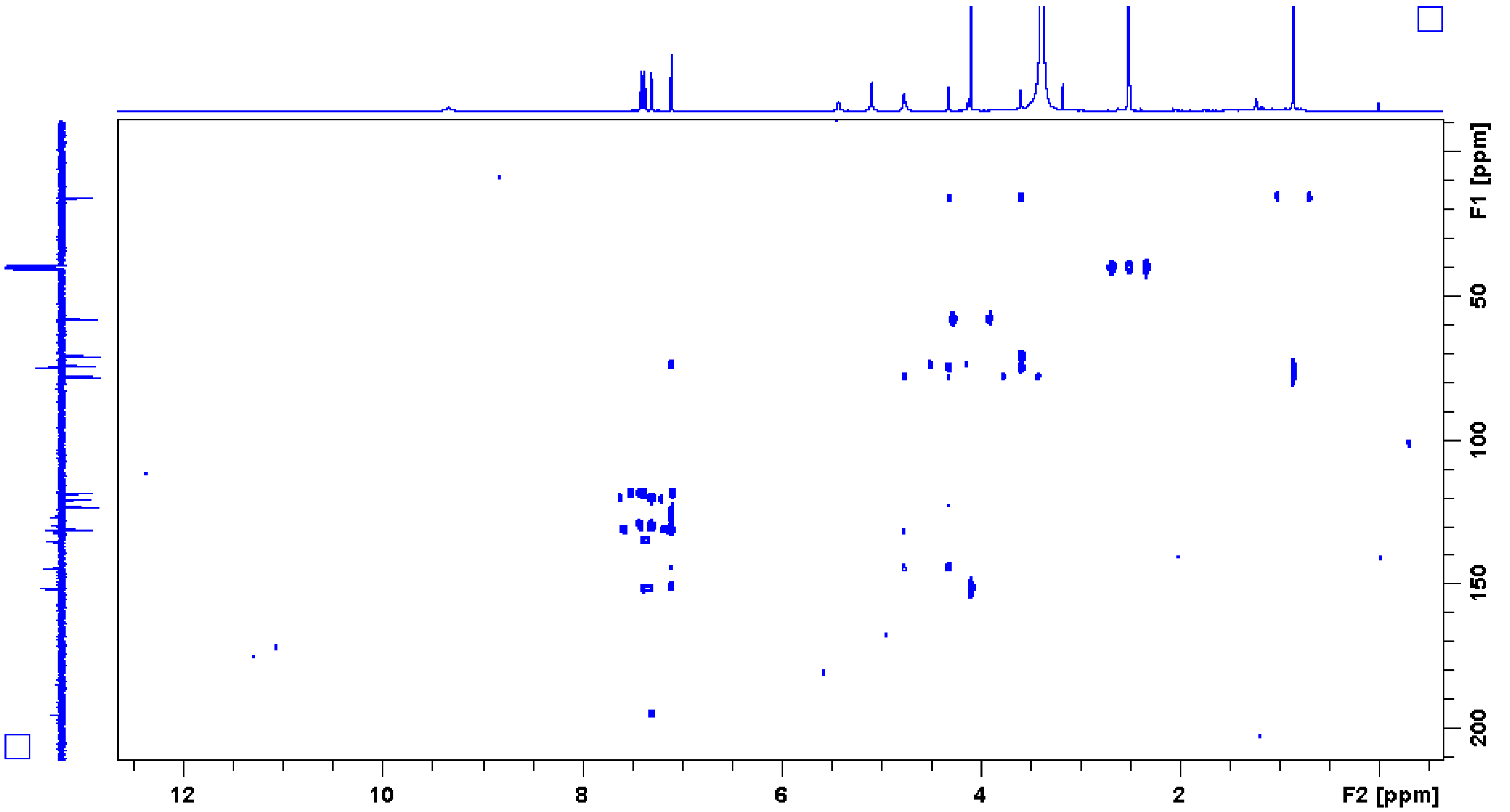

Figure S35. The ROESY spectrum of compound **4** in DMSO-d_6

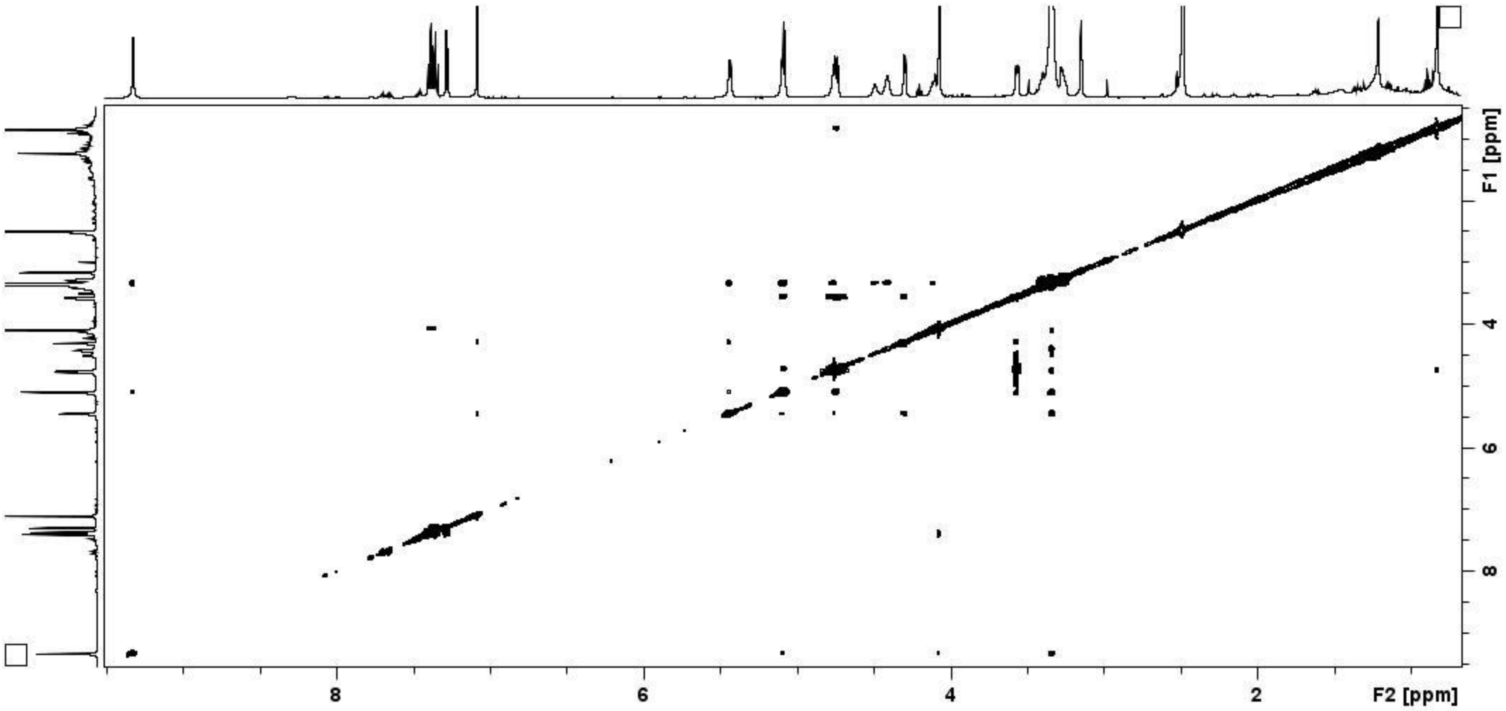

Figure S36. The HRESIMS spectrum of compound **4**

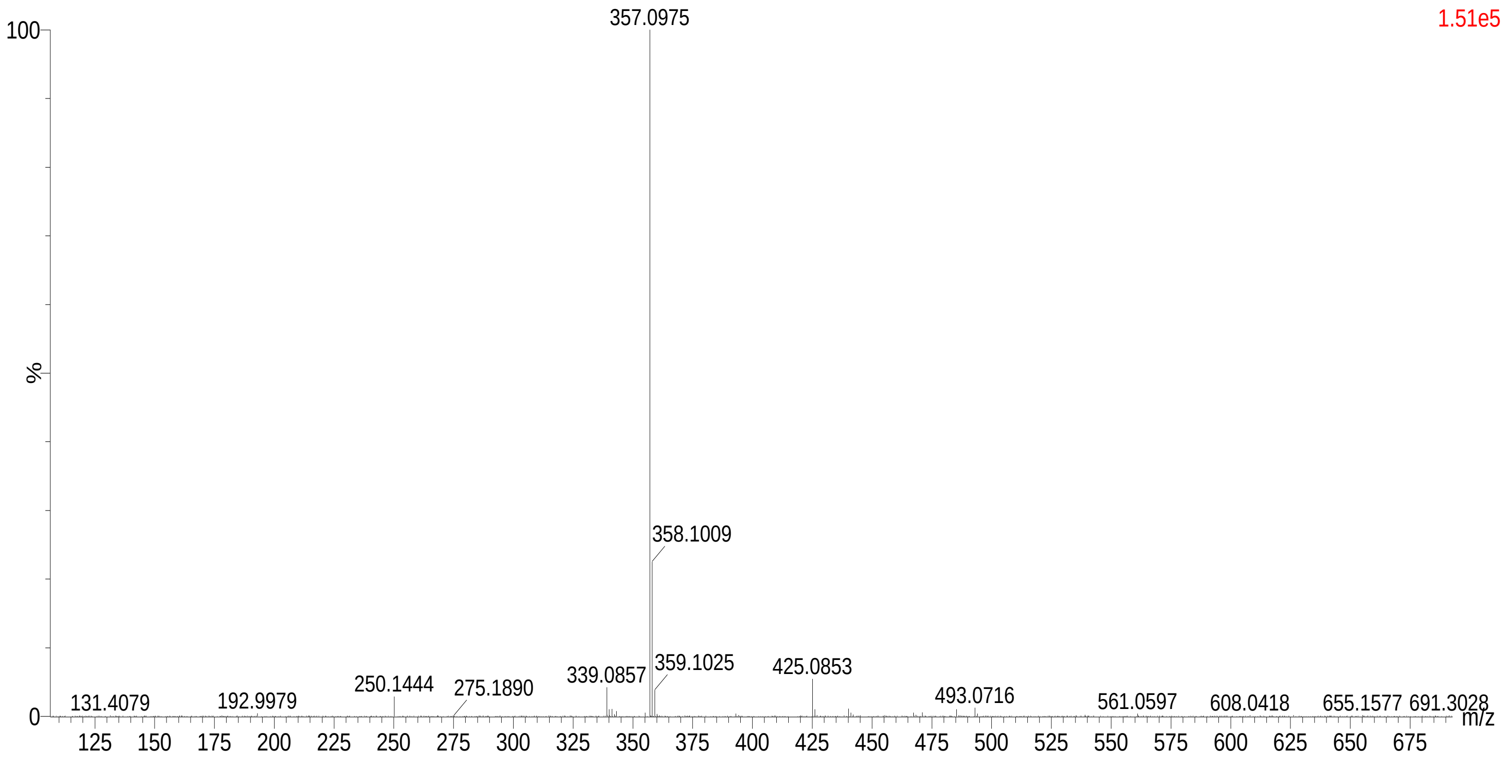

Figure S37. The IR spectrum of compound **4**

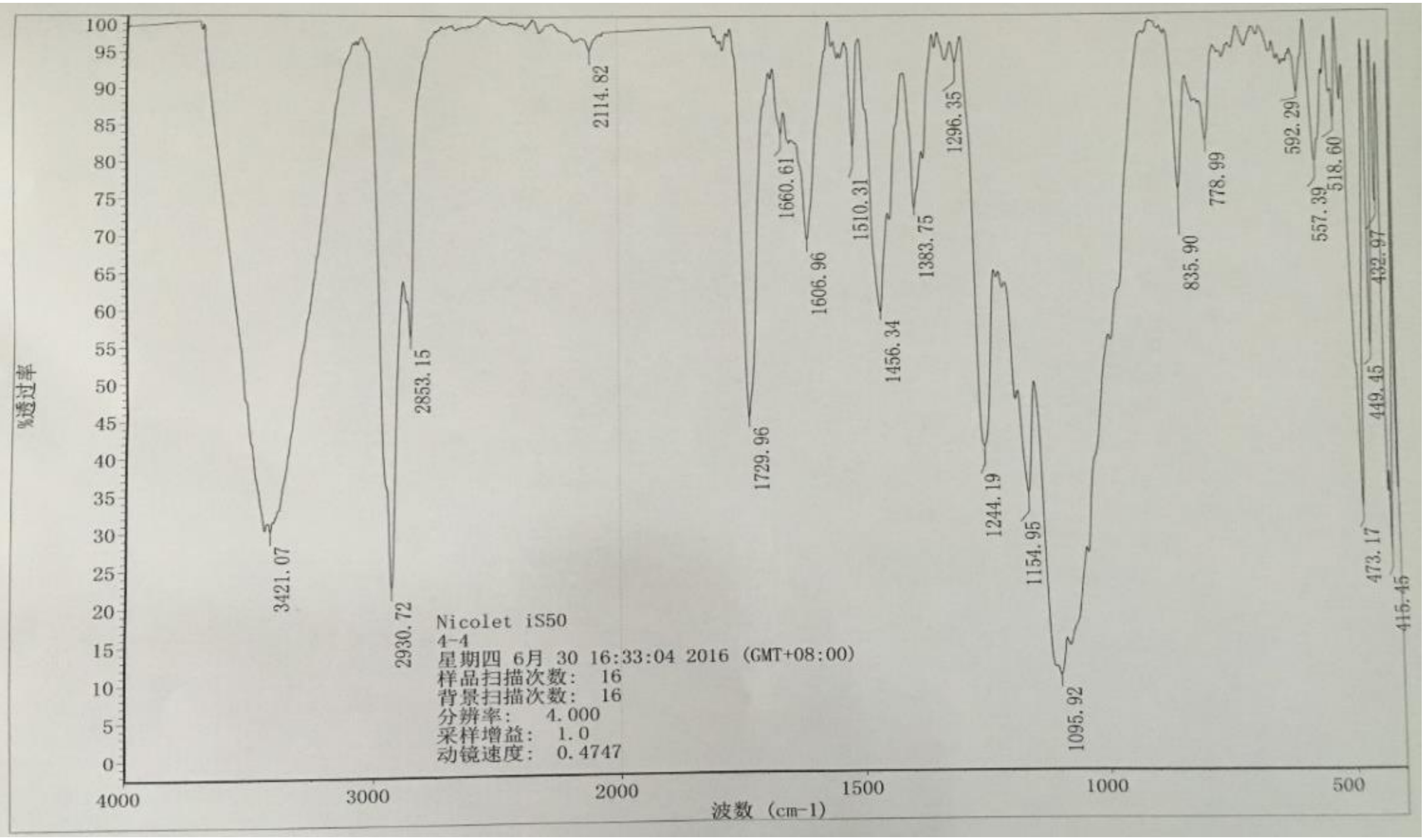

Figure S38. The ^{1}H NMR (600 MHz) spectrum of compound **5** in DMSO-d_6

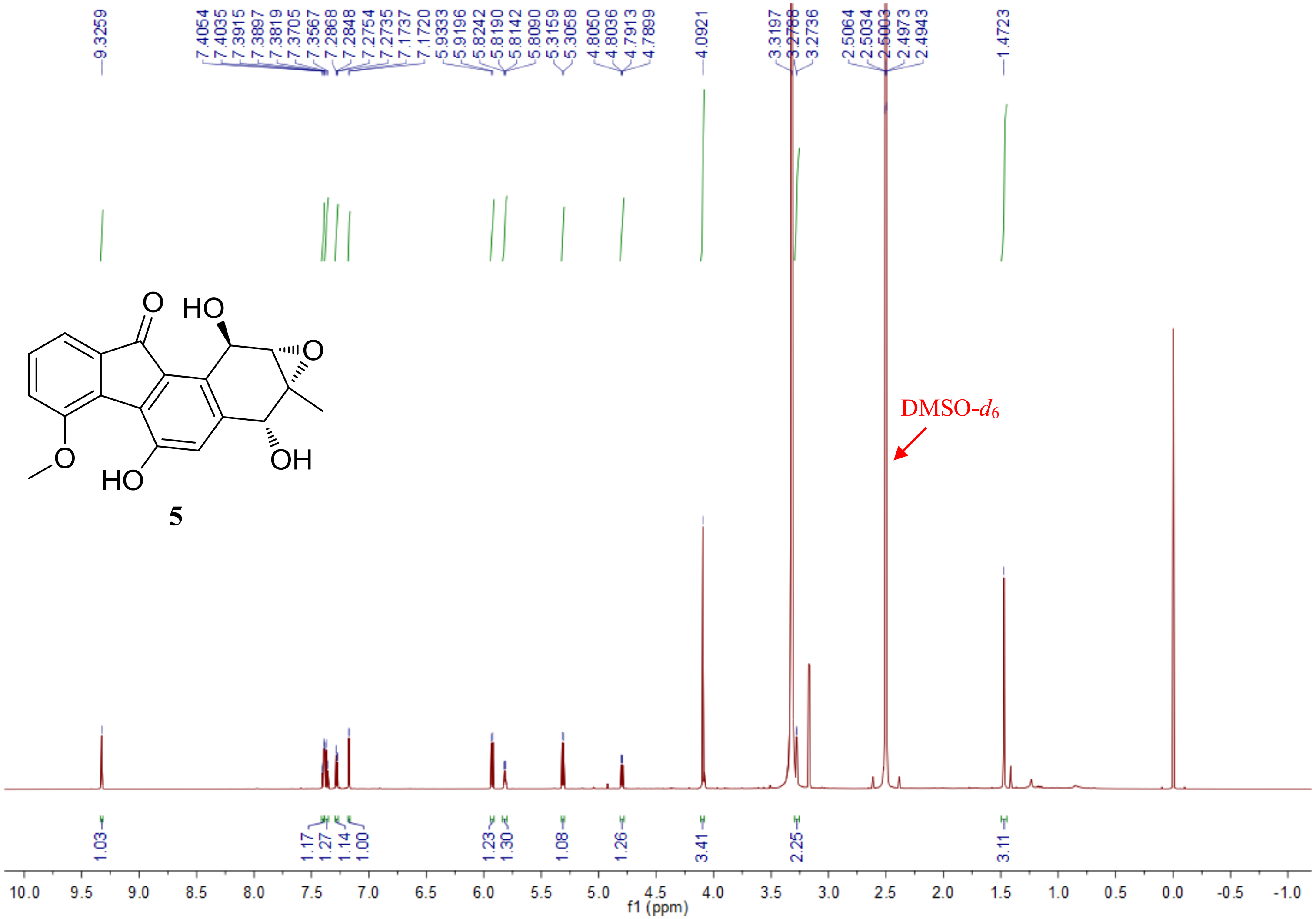

Figure S39. The ^{13}C NMR (150 MHz) spectrum of compound **5** in DMSO-d_6

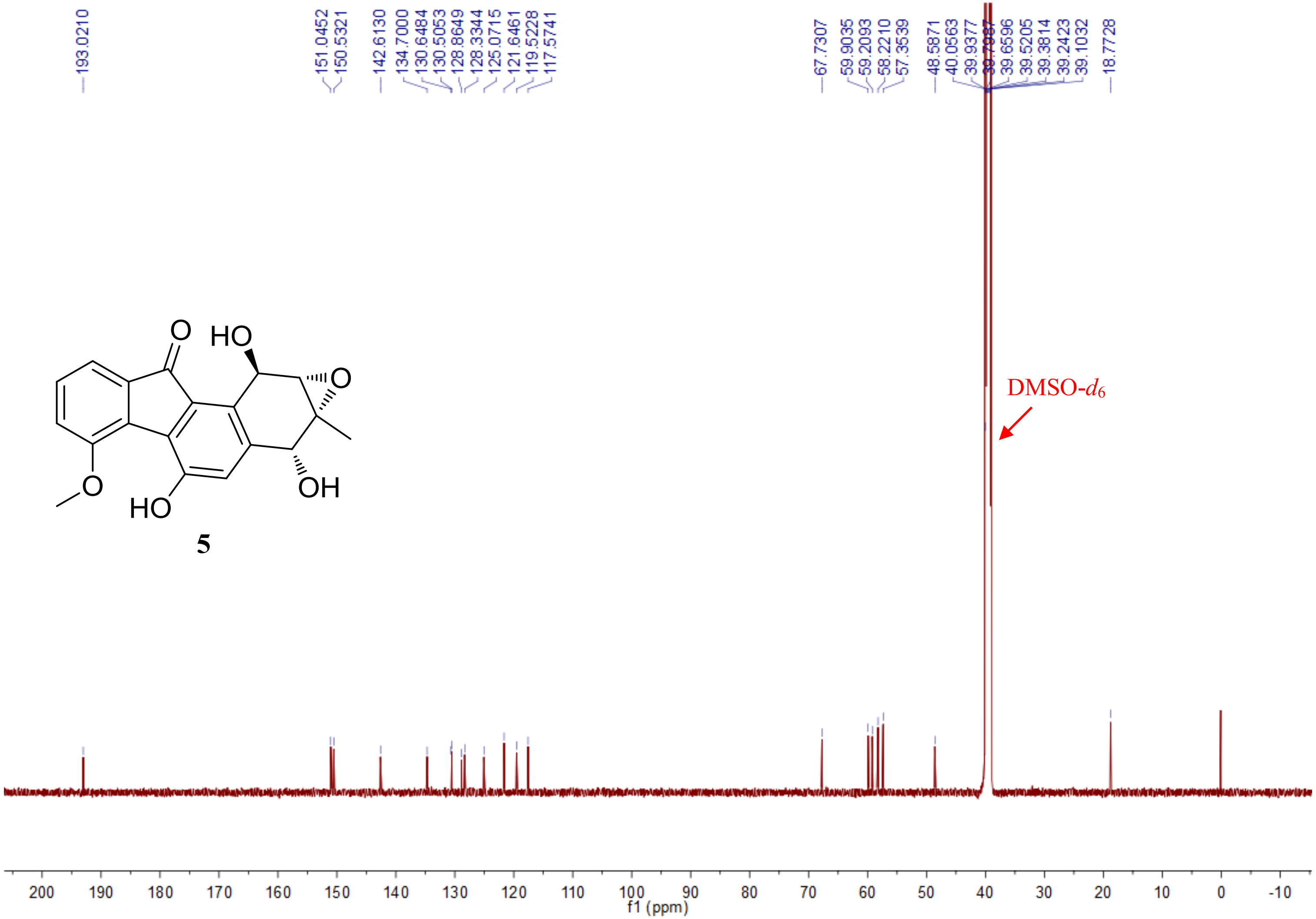

Figure S40. The COSY spectrum of compound **5** in DMSO-d_6

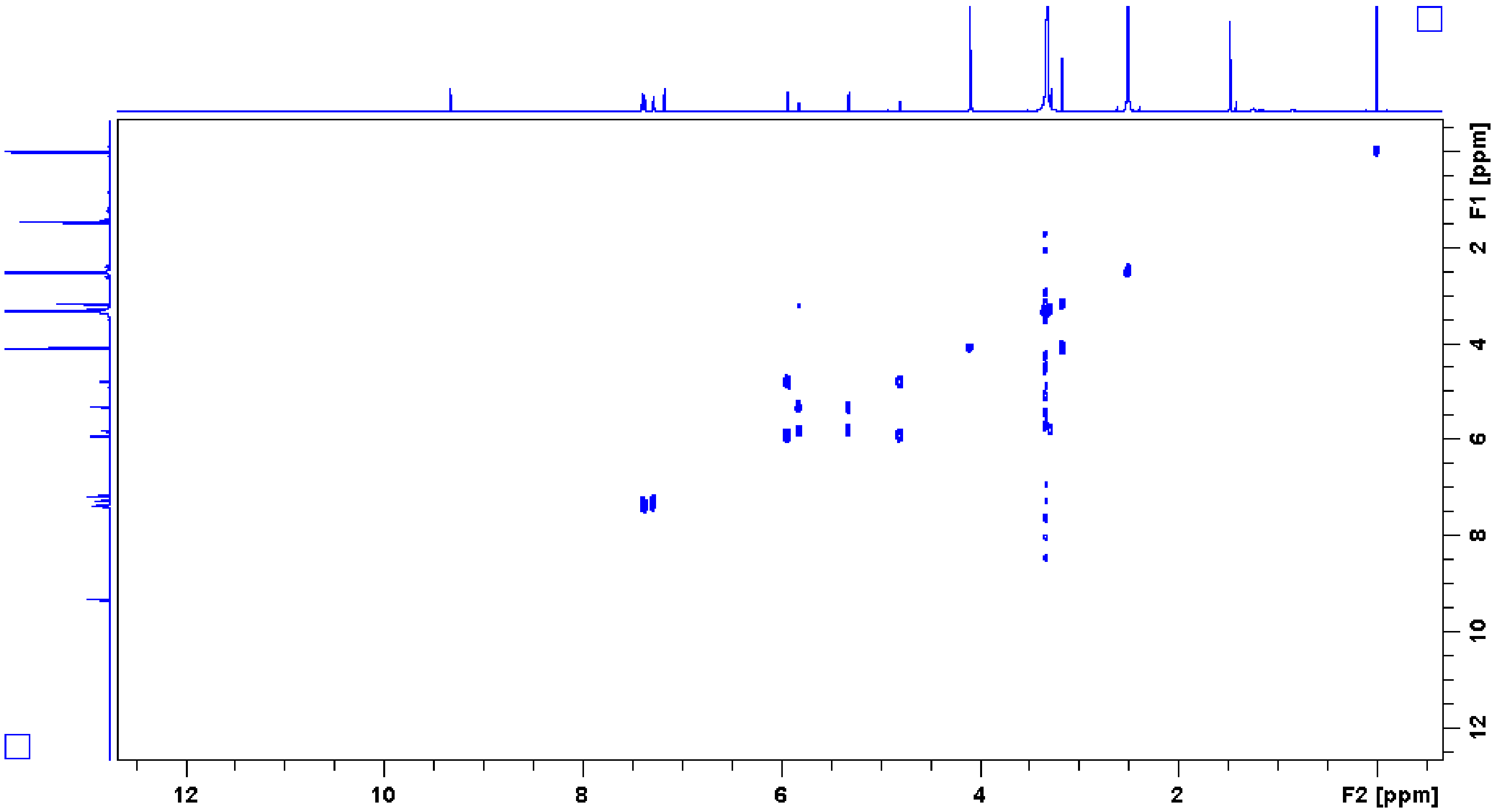

Figure S41. The HSQC spectrum of compound **5** in DMSO-d_6

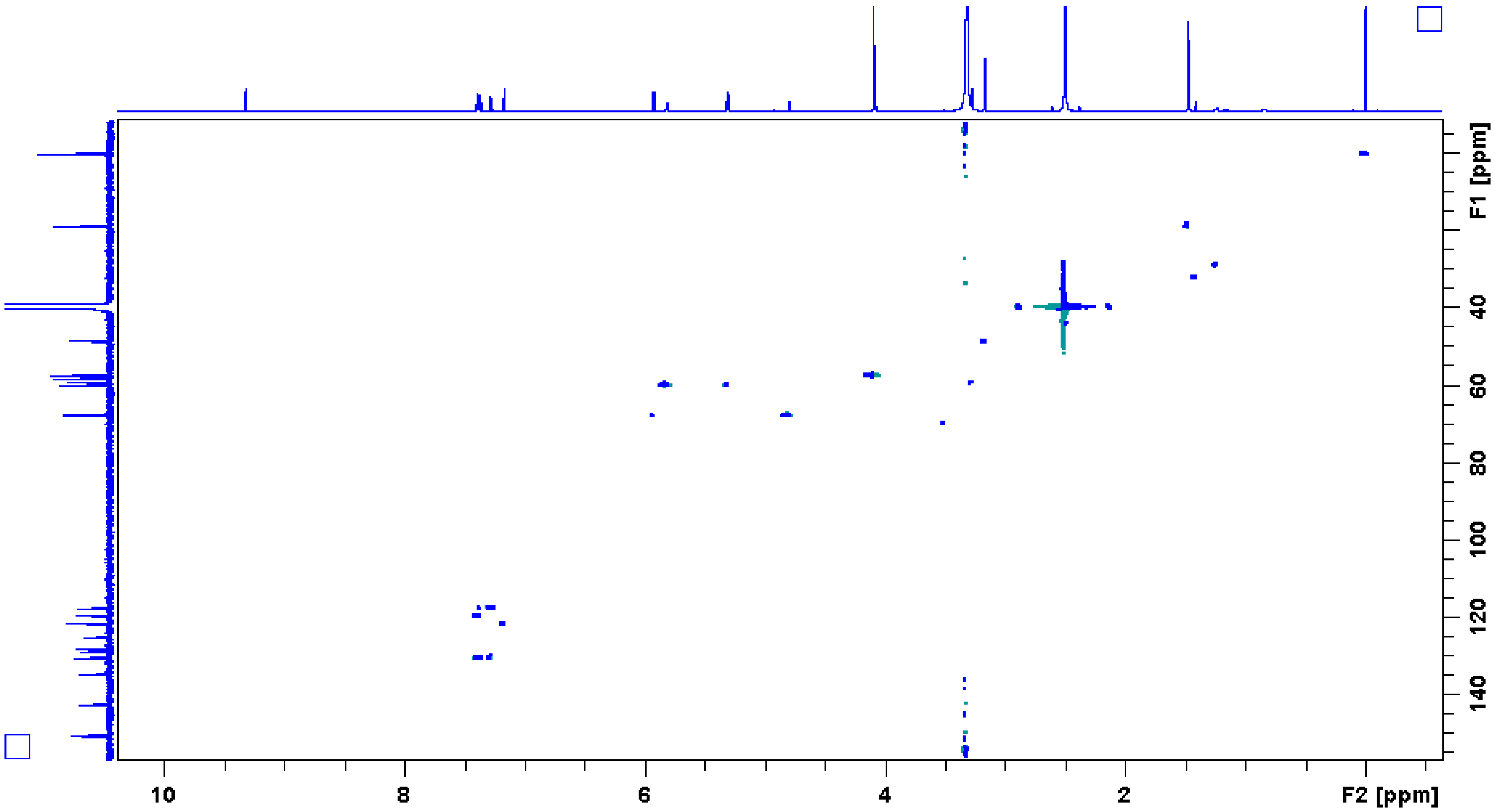

Figure S42. The HMBC spectrum of compound **5** in DMSO-d_6

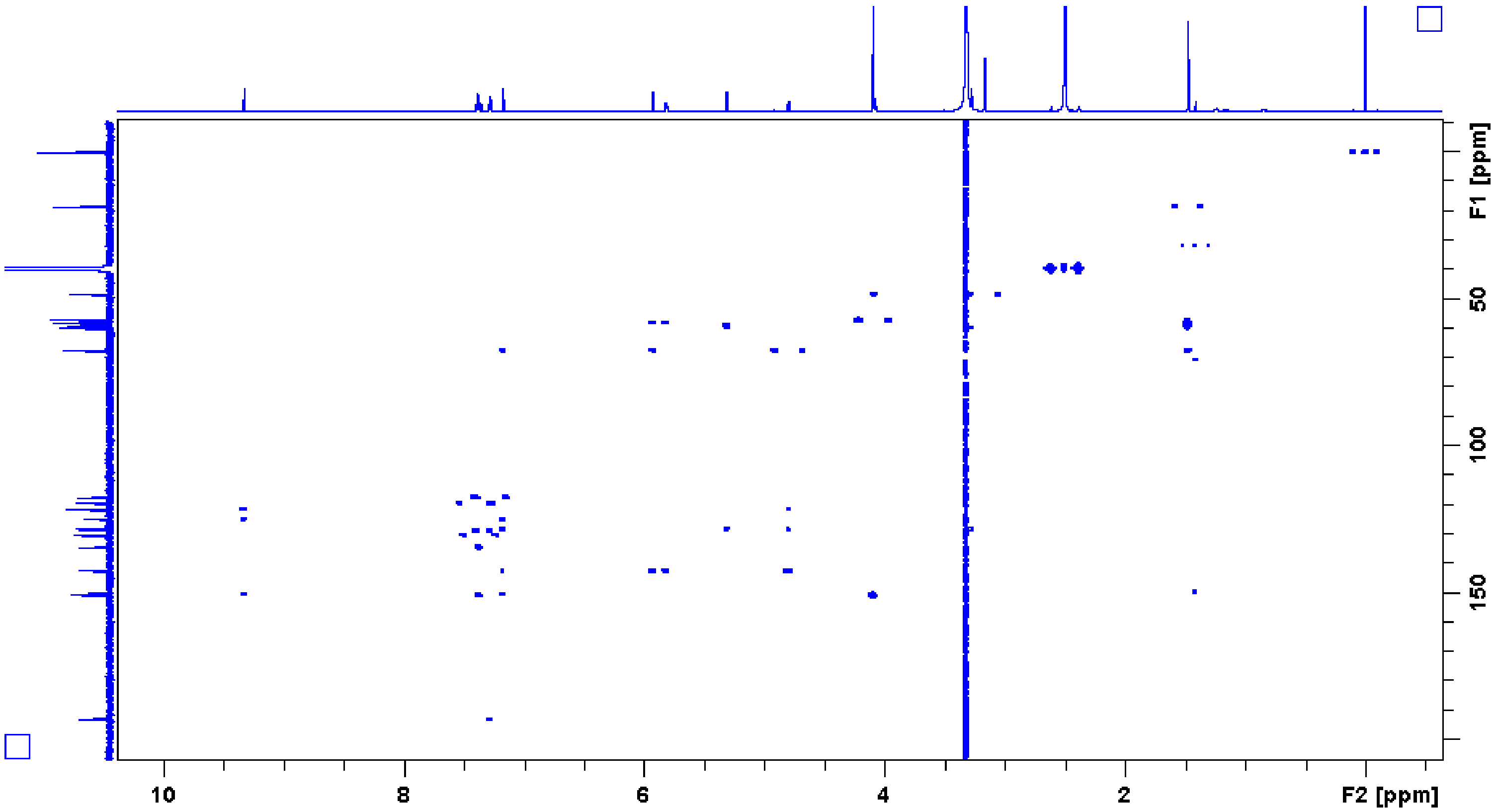

Figure S43. The ROESY spectrum of compound **5** in DMSO-d_6

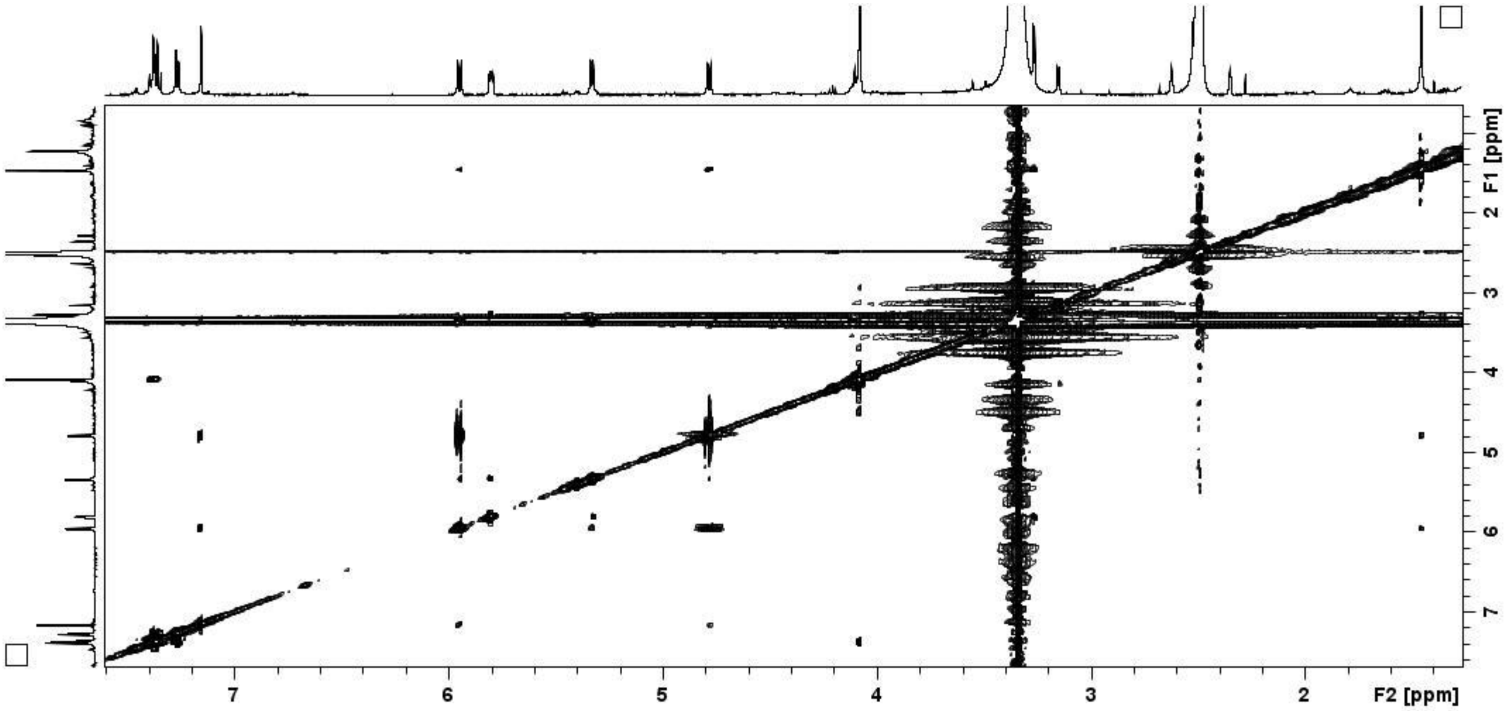

Figure S44. The HRESIMS spectrum of compound **5**

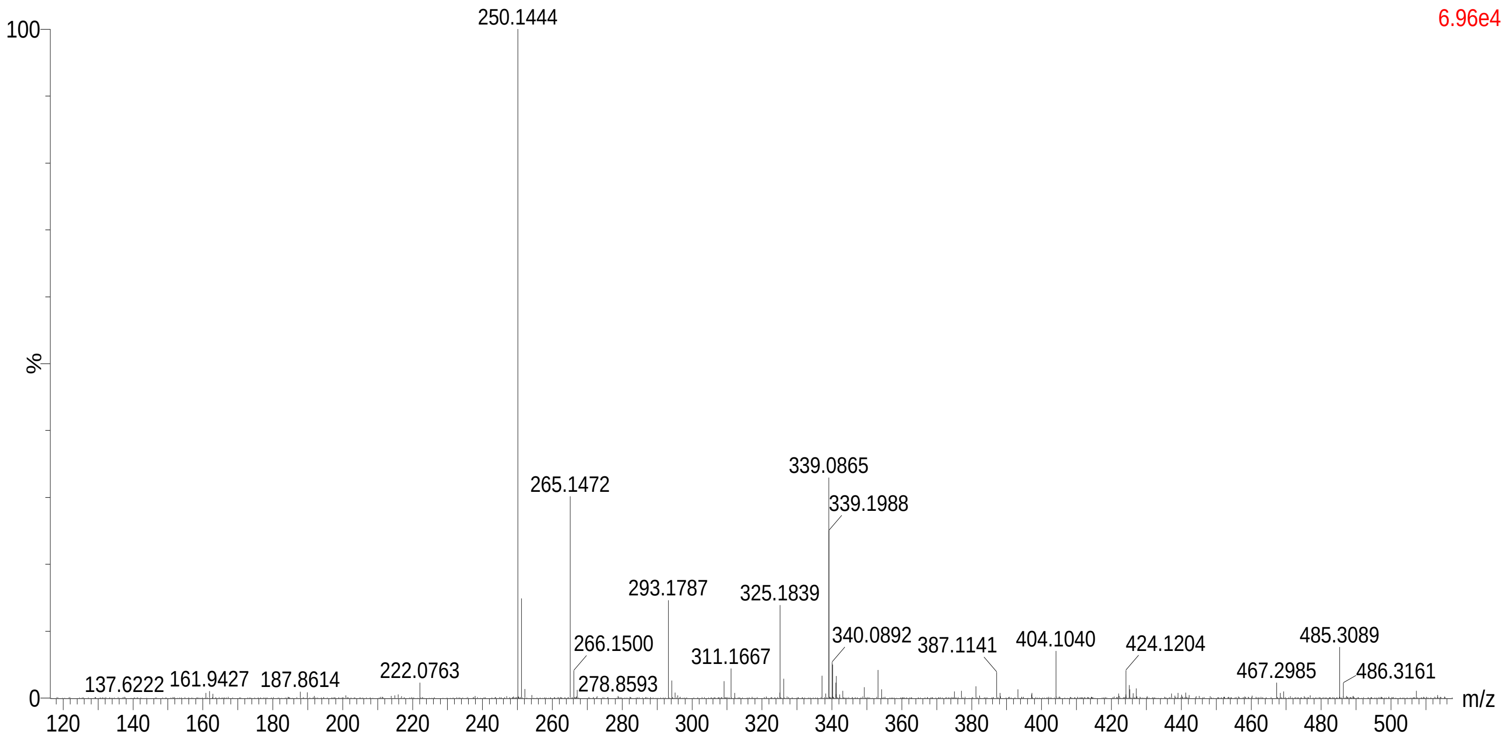

Figure S45. The IR spectrum of compound **5**

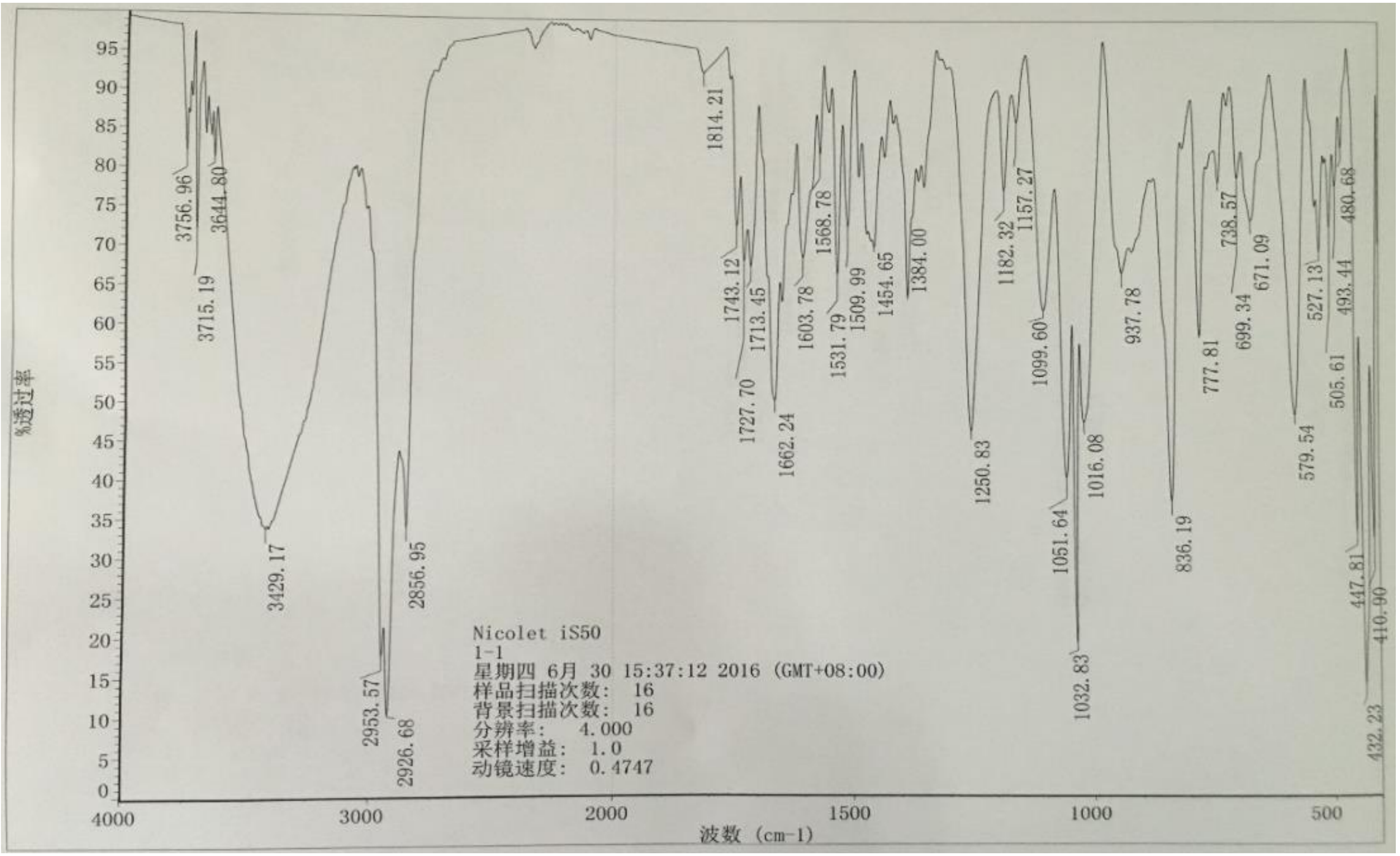

Supplement: Supplementary file 1 [file marinedrugs-16-00087-s001.pdf]
